# Utilization of Plant Architecture Genes in Soybean to Positively Impact Adaptation to High Yield Environments

**DOI:** 10.3389/fpls.2022.891587

**Published:** 2022-05-24

**Authors:** Jeong-Hwa Kim, Andrew Scaboo, Vincent Pantalone, Zenglu Li, Kristin Bilyeu

**Affiliations:** ^1^Division of Plant Science and Technology, University of Missouri, Columbia, MO, United States; ^2^Department of Plant Sciences, University of Tennessee, Knoxville, TN, United States; ^3^Department of Crop and Soil Sciences, University of Georgia, Athens, GA, United States; ^4^Plant Genetics Research Unit, United States Department of Agriculture-Agricultural Research Service, University of Missouri, Columbia, MO, United States

**Keywords:** soybean, plant architecture, stem termination types, agronomic traits, southern environments

## Abstract

Optimization of plant architecture by modifying stem termination and timing of flowering and maturity of soybean is a promising strategy to improve its adaptability to specific production environments. Therefore, it is important to choose a proper stem termination type and to understand morphological differences between each stem termination type under various environmental conditions. Variations in abruptness of stem termination have been generally classified into three classical genetic types, indeterminate (*Dt1*), determinate (*dt1*), and semi-determinate (*Dt2*). However, an additional stem termination type, termed tall determinate, and its genetic symbol, *dt1-t*, were introduced about 25 years ago. The tall determinate soybean lines show delayed cessation of apical stem growth and about 50% taller plant heights than the typical determinate soybeans, even though the genetic control of the tall determinate phenotype was found to be allelic to *dt1*. Despite the potential agronomic merits of the alternative stem termination type, knowledge about the tall determinate soybean remains limited. We clarified the molecular basis of the tall determinate stem termination type and examined potential agronomic merits of the alternative stem type under three different production environments in the US. Sequence analysis of the classical tall determinate soybean lines revealed that the *dt1-t* allele responsible for tall determinate stem architecture is caused by two of the identified independent missense alleles of *dt1, dt1-t1* (R130K), and *dt1-t2* (R62S). Also, from the comparison among soybean accessions belonging to each of the genotype categories for stem termination types, soybean accessions with tall determinate alleles were found to have a high discrepancy rate in phenotyping. Newly developed tall determinate late-maturing soybean germplasm lines had taller plant heights and a greater number of nodes with a similar stem diameter and similar pod density at the apical stem compared to typical determinate soybeans having *dt1* (R166W) alleles in Southern environments in the US. The phenotype of increased pod-bearing nodes with lodging resistance has the potential to improve yield, especially grown in high yield environments. This study suggests an alternative strategy to remodel the shape of soybean plants, which can possibly lead to yield improvement through the modification of soybean plant architecture.

## Introduction

Soybean [*Glycine max* (L.) Merrill] is an important crop worldwide, and improving its yield has always been a major concern. Long-term efforts for improving yield have led to world soybean production, increasing ~13-fold from 1961 to 2017 (FAO, 2017). Global yield increase was mainly due to an increase in the planting area; the yield per unit area of soybean. Genetic gain for soybean in the US has been ~34 kg/ha^.^yr (0.5 bu/ac^.^yr) (Rincker et al., 2014; Boehm et al., 2019). Unlike other major crops, which increased yields per unit area by increasing planting densities with the modification of plant architecture, improving yields per unit area in soybean is not that simple due to its unique plant architecture. Since soybean is a typical legume, which has leaves, inflorescences, and pods growing at each node, several agronomic traits, such as plant height, number of nodes, flowering time, and maturity, need to be considered simultaneously to optimize soybean plant architectures for improved yield potential.

Soybean plant architecture can be modified by adjusting stem termination and timing of flowering and maturity. Soybeans show a wide range in the abruptness of stem termination, and the variations primarily result from the differences in timing at which soybeans terminate their apical stem growth (Bernard, 1972). Based on the timing, most soybean cultivars are generally classified into three categories: indeterminate, determinate, and semi-determinate. The indeterminate soybean cultivars continue vegetative growth on apical meristems at the stem and branch apices after flowering until the beginning of seed fill (Bernard, 1972). Since the terminal growth continues as long as lateral growth, indeterminate cultivars feature a tapered stem with little or no secondary lateral growth near the stem tip (Tian et al., 2010). In contrast, determinate cultivars abruptly halt vegetative growth at or soon after flowering begins, producing a thick stem due to the continuous lateral growth even after the cessation of apical stem growth (Ping et al., 2014). In semi-determinate cultivars, the stem tip ceases vegetative growth several days earlier than the indeterminate ones, resulting in fewer main-stem nodes and somewhat shorter stem length than the indeterminate cultivars (Hartung et al., 1981; Ping et al., 2014). Classical genetic analyses identified two genes regulating the stem growth habit in soybean, *Dt1* and *Dt2* (Bernard, 1972). In the *Dt1* genetic background, *Dt2* genotypes have semi-determinate phenotypes, while the recessive allele of the *dt2* gene produces indeterminate cultivars. However, in the *dt1* genetic background, the phenotype is determinate regardless of the alleles at the *Dt2* locus due to an epistatic effect of the recessive *dt1* allele with the *Dt2* alleles.

While the three categories of stem termination types were generally accepted, Thompson et al. (1997) introduced an additional type, termed tall determinate, and its genetic symbol, *dt1-t*, to describe a distinct phenotype they found. The tall determinate phenotype was observed from near-isogenic lines (hereafter referred to as isolines) of an indeterminate soybean cultivar “Clark” with either “Soysota” or “Peking” as a donor parent of L91-8052 and L91-8060, respectively. The authors found that the tall determinate (*dt1-t*) isolines significantly delayed the timing of stem termination compared to typical determinate (*dt1*) isolines and almost identically terminated their stem growth with semi-determinate (*Dt1Dt2*) isolines, even though the genetic control of the tall determinate phenotype was later found to be allelic to *dt1* and independent of *Dt2* (Thompson et al., 1997). The tall determinate isolines showed similar plant height to the semi-determinate isolines, which were ~70% of the mature plant height of indeterminate Clark; however, the tall determinate isolines had significantly different numbers of final stem nodes and terminal leaflet areas compared to the semi-determinates. When considering the leaf and stem characteristics at the top of the plant, tall determinate isolines were similar with determinate isolines. Given that the stem termination type affects other important agronomic traits, such as flowering time, node formation, plant height, lodging resistance, and, ultimately, yield of soybeans, more specific research about the distinct stem characteristics of the tall determinate type is required in order to characterize and utilize its potential agronomic merits, but no further articles have been published to date (Heatherly and Smith, 2004; Liu et al., 2010; Cao et al., 2017).

The molecular basis of the typical stem growth habits in soybean was elucidated. *Dt1* (*GmTfl1*; Glyma.19g194300), the major gene affecting stem termination, was found to be a functionally conserved ortholog of *Arabidopsis thaliana TERMINAL FLOWER1* (*TFL1*), where the functional gene participates in forming indeterminate stems (Liu et al., 2010; Tian et al., 2010). Six independent missense mutations were identified while searching for allelic variations of the *Dt1* genic region (Liu et al., 2010; Tian et al., 2010; Yue et al., 2021). While four out of the six identified non-synonymous mutations were suggested to cause the transition from indeterminate to determinate phenotypes, the specific functions of each of the resulting alleles and possible differences in phenotypes conferred from each of these mutations have not been defined. *Dt2*, the second soybean gene regulating stem growth, was characterized as a dominant MADS domain factor gene classified into the *APETALA1/SQUAMOSA* (*AP1/SQUA*) subfamily (Ping et al., 2014). There were quantitative differences in the expression level between dominant *Dt2* and recessive *dt2*, and the increased expression of *Dt2* was found to downregulate functional *Dt1* in the shoot apical meristems (SAMs) to promote early conversion of the SAMs into reproductive inflorescences (Ping et al., 2014). Along with its function as a direct repressor of *Dt1, Dt2* was found to activate the putative floral integrator/identity genes, such as *GmSOC1, GmAP1*, and *GmFUL*, thereby promoting flowering in soybeans (Zhang et al., 2019). A total of 37 SNPs in the non-coding region of *Dt2* were identified when it was cloned, but further efforts are still required to pinpoint causative mutations and elucidate molecular mechanisms responsible for the *Dt2* activity (Ping et al., 2014).

The genes regulating stem termination contribute diversity in phenotypes of soybean plant architecture, and each of these morphological characteristics has distinct advantages depending on diverse production environments. For instance, nearly all soybean cultivars grown commercially in the northern US and in Canada (MG IV and earlier) are indeterminate types due to their better adaptation to the shorter growing season at high latitudes, while most soybean cultivars commercially grown in the southern US (MG V and later) are determinate, although, recently, a shift to indeterminate types has been occurring in some southern environments (Bernard, 1972; Hartung et al., 1981; Heatherly and Smith, 2004). However, depending on environments, there would be agronomic merits that can be achieved by choosing an alternate stem termination type for soybeans. In separate studies in the southern US, depending on the specific environmental conditions, indeterminate or determinate soybean lines had higher yields, and there were significant growth habit x environment effects (Ouattara and Weaver, 1994; Kilgore-Norquest and Sneller, 2000). Moreover, semi-determinate soybean cultivars have been developed in the past decade to be particularly used in high-yield, lodging prone-irrigated northern environments due to improved productivity in those environments (Ping et al., 2014). Given that the distinct stem characteristics each soybean has are associated with its production, a precise classification of stem termination types is required in order to optimize plant architecture suitable for the production environment (Liu et al., 2010, 2020).

Germplasm collections, which contain phenotype data of stem growth habits, as well as various other associated traits, such as plant height, lodging, flowering and maturity dates, and yield, are publicly accessible from the USDA National Plant Germplasm System and Germplasm Resources Information Network (GRIN) database collection (www.ars-grin.gov/). Soybean accessions having a wide range in the abruptness of stem termination were evaluated by two separate descriptors, termed stem termination score and stem termination type. In terms of the stem termination score, the evaluations were made at maturity, with a number ranging from 1 (very determinate) to 5 (very indeterminate). Stem termination scores <2 and ≥2.5 are considered determinate and indeterminate, respectively, and a score in between 2 and 2.5 is considered semi-determinate (Heatherly and Smith, 2004). With regard to the stem termination type, soybean accessions were coded based on characteristics at the top of the stem with three categories: D (determinate, stems abruptly terminating), I (indeterminate, stems tapering gradually toward tips), and S (semi-determinate, intermediate between determinate and indeterminate). Considering the measurements are somewhat subjective and possibly influenced by environmental factors, these simple classifications may lead to discrepancies between the designated stem termination type and the actual underlying genotype of the evaluated soybean accessions.

Alteration of plant architecture by modifying genes that affect stem termination and timing of flowering and maturity to balance development of pod bearing nodes with lodging resistance is one strategy to improve yield potential of soybeans. However, soybeans show a wide range in the abruptness of stem termination, and the variations are even further complicated to definitively categorize because of other genetic and environmental factors, particularly flowering time and day length. Therefore, a precise classification of stem termination types, as well as a broad understanding of their responses under various environments in combination with other genes, is required to choose a proper plant architecture suitable for the production environment (Liu et al., 2010, 2020). In particular, the potential agronomic merits of the new stem termination type, tall determinate, which has not been widely studied, need to be examined for its broader use in breeding programs. In this study, we clarified the molecular basis of the *dt1-t* alleles controlling the tall determinate stem termination type and examined the morphological characteristics and possible agronomic merits of the tall determinate stem termination type in three different latitudinal environments, ranging from MG III to VII in the US.

## Materials and Methods

### Sequencing Analysis at *Dt1* Locus for the Classical Tall Determinate Soybean Lines

A total of four classical tall determinate soybean lines, Peking (PI 548402), Soysota (PI 548417), L91-8060 (PI 591488), and L91-8052 (PI 591487), obtained from the USDA National Plant Germplasm System, were used in the sequence analysis to clarify the molecular basis of the *dt1-t* alleles controlling the tall determinate stem termination type. Peking and Soysota are soybean cultivars, and L91-8060 and L91-8052 are isolines, with the soybean cultivar Clark as a recurrent parent and each of Peking and Soysota as a donor parent, respectively. Details of the development of the two isolines were described in Thompson et al. (1997). Since the tall determinate allele was found to be allelic to *dt1* (Thompson et al., 1997), the nucleotide polymorphisms in the coding region of *Dt1* (Glyma.19g194300; 45183357 – 45185175 Wm82.a2.v1) were examined (Table 1).

**Table 1:** Description of alleles and genes controlling stem termination type and maturity used in this study.

**Gene**	**Gene identifier**	**Allele**	**Polymorphism**	**Position**	**Protein change**	**References**
*Dt1* (*GmTFL1*)	Glyma.19g194300	*dt1-t2*	SNP: C/A	45184804	R62S	Tian et al., 2010
		dt1*	SNP: G/A	45183859	P113L	Tian et al., 2010
		*dt1-t1*	SNP: C/T	45183808	R130K	Tian et al., 2010
		*dt1*	SNP: T/A	45183701	R166W	Tian et al., 2010
*Dt2*	Glyma.18g273600	*dt2*	SNP: G/A	55642486		Ping et al., 2014
*E1*	Glyma.06g207800	*e1-as*	SNP: C/G	20207322	T15R	Xia et al., 2012
*E2*	Glyma.10g221500					Watanabe et al., 2011
*E3*	Glyma.19g224200					Watanabe et al., 2009

*Positions are based on the Williams 82 soybean reference genome sequence (Wm82.a2.v1). Dt1 (Glyma.19g194300) is on the reverse strand in the genomic sequence; thus, SNPs at the locus are reverse complement on Chromosome 19. The recessive dt2 was designated based on allele status at the position of an associated intron variant (Pos: 55642486 on Chromosome 18)*.

Whole genome resequenced data of the two tall determinate cultivars, Peking and Soysota, were publicly available (Zhou et al., 2015), so the nucleotide polymorphisms at the *Dt1* locus were investigated in the sequence data using positions on Wm82.a2.v1 chromosome 19 (Table 1). The data were downloaded from the National Center for Biotechnology Information (NCBI) Sequence Read Archive (SRA) (https://phytozome-next.jgi.doe.gov/). The data sets were processed with a custom Pgen workflow and analyzed on SNPViz v2.0 (Zeng et al., 2021). For the two tall determinate isolines, L91-8052 and L91-8060, Sanger sequencing of PCR amplified products was conducted for checking allele status at the locus. Template DNA was prepared from leaf presses from 1.2-mm washed FTA (Whatman, Clifton, NJ) card punches according to the manufacturer's instructions. Fully developed trifoliate leaves were used to prepare the FTA card punches. The DNA templates were amplified by PCR using the two primer sets, which are targeting each of exon 1 and exon 4 regions at the *Dt1* locus where the missense R62S and P113L, R130K, and R166W alleles are located, respectively: a symmetric mix of primers (Dt1upf1: 5'-CACACTCGATCTACCT-3', Dt1in1r: 5'- ACATACCGTGTGACCATG-3'), with a product size of 485 bp targeting exon 1, and a symmetric mix of primers (Dt1in31f: 5'-CATGAGAGAGATCACTGAC−3', Dt1endr1; R: 5'-GCAAAACCAGCAGCTACTT-3'), with a product size of 292 bp targeting exon 4 region at *Dt1* locus. The total volume of reactions was 20-μl containing-templates DNA, primers, a buffer [40-mM Tricine- KOH (pH 8.0) 16-mM KCl, 3.5-mM MgCl_2_, 3.75-μg ml^−1^ BSA], 5% DMSO, 200-μM dNTPs, and 0.2 X Titanium Taq polymerase (BD Biosciences, Palo Alto, CA). The cycling conditions were 95°C for 3 min, 40 cycles of 95°C for 20 s, 60°C for 20 s and 72°C for 30 s, final extension at 72°C for 3 min, and ended at 4°C. The PCR products were determined by 1.2% agarose gel electrophoresis and then sequenced using each of the forward primers at the University of Missouri DNA Core Facility (https://dnacore.missouri.edu/). Sequencing results were analyzed using Chromas software.

### Analysis on Disparity in Measuring Stem Termination Types

To examine how historically observers have phenotyped stem termination types of soybean accessions, a set of 528 soybean accessions of which both phenotypes and resequenced data are publicly accessible were selected for this analysis. The detailed information of the accessions is listed in Supplementary Table 1. The phenotype data on stem termination type were downloaded from The USDA National Plant Germplasm System and Germplasm Resources Information Network (GRIN) database collection (www.ars-grin.gov). The soybean accessions were evaluated with three categories: D (determinate), I (indeterminate), and S (semi-determinate), depending on the morphological characteristics at the stem tip. The publicly available resequenced data of the soybean accessions were analyzed from our curated accession panel of 775 soybean accessions with whole genome resequence data. The Soy775 resource is comprised of data sets from the USB-481 resequencing project and Zhou302 data set remapped to Wm82.a2.v1 and available on soykb.org (Zhou et al., 2015; Valliyodan et al., 2016, 2021; Škrabišová et al., In press). The genotype categories of each soybean accession were assigned based on the allele status at the two genes, conferring stem termination types, *Dt1* and *Dt2*. In terms of the *Dt1* gene, the allele status of each line was sorted depending on the presence of one of four of the most frequently found alleles, *dt1-t2* (R62S), dt1^*^ (P113L), *dt1-t1* (R130K), and *dt1* (R166W), out of the six previously identified missense mutations. For the *Dt2* allele, we selected a variant genomic position (Pos: 55642486 on Chromosome 18) in the second intron of Glyma.18g273600 based on preliminary analyses of the cloned gene using our SNPViz haplotype viewer tool (Ping et al., 2014; Langewisch et al., 2017) and determined that this position had high accuracy to the GWAS hit on Chromosome 18 (ss715632223) for semi-determinate stem architecture (Bandillo et al., 2017). The genotype at the *Dt2* locus was assigned based on the nucleotide polymorphism at the genomic position of the marker: functional *Dt2* in presence of nucleotide G, and recessive *dt2* in presence of nucleotide A. The disparity rates of each genetic category were calculated based on the distribution of observed phenotypes for accessions categorized by their *Dt1* and *Dt2* allele status. For example, for the 292 accessions with the genotype *Dt1 dt2*, 92.8% were scored indeterminate (matching the predicted phenotype), while 5.8% were scored semi-determinate, and 1.4% were scored determinate.

### Field Experiment in Three Distinct Maturity Group Environments in the United States

To evaluate morphological characteristics and potential agronomic merits of soybean lines with various plant architecture types, soybean lines with each of the targeted genotype combinations were planted in three different latitudinal environments [maturity groups (MGs) III, V, and VI] in the US in 2019 and 2020. Agronomic traits were measured from the soybean plants produced in each of the production environments. The details of each field experiment are summarized in Supplementary Table 2.

### Plant Materials and Population Development

The plant materials used for each of the three field trials are listed in Table 2. We developed recombinant inbred line (RIL) populations, having either the tall determinate or semi-determinate stem termination alleles, along with the functional *E1* maturity gene, which is critical to southern US soybean production environments (Langewisch et al., 2017). Four experimental populations were generated for this study, and the details of population development are summarized in Supplementary Table 3. Each population had one donor parent of the functional *E1* maturity gene and one donor parent of the target stem termination alleles, either tall determinate [Glyma.19g194300 *dt1-t1* (R130K) or *dt1-t2* (R62S)] or semi-determinate (Glyma.18g273600 intron SNP). As the functional *E1* donor, two soybean cultivars were utilized. “Jake” is a high-yielding soybean cultivar with determinate stem termination type and MG V, which was released by the University of Missouri (Shannon et al., 2007). “Ellis” is a high-yielding late MG IV determinate soybean cultivar developed by the University of Tennessee (Pantalone et al., 2017). As the donor of target stem termination types, three soybean lines were utilized. L91-8060 and L91-8052 are the classical tall determinate isolines, which were generated from crosses between a soybean cultivar Clark and either soybean cultivars, Soysota or Peking, respectively (Thompson et al., 1997). LG90-2550 (PI 602995) is an MG III semi-determinate soybean line cooperatively developed and released in 1997 by the USDA-ARS and the Illinois Agricultural Experiment Station (Thompson et al., 1999). The four populations made for this study were as follows (Supplementary Table 3): Cross 1 was KB17-16: L91-8052, the tall determinate isoline carrying *dt1-t1* (R130K) allele, was crossed to Jake, the high-yielding MG V soybean cultivar, having the functional *E1* allele. Cross 2 was KB17-17: L91-8060, the other tall determinate isoline having *dt1-t2* (R62S) allele, was crossed to Jake. Cross 3 was KB17-7: LG90-2550, the semi-determinate soybean line carrying *Dt2* allele, was crossed to Jake. Cross 4 was KB17-8: LG90-2550 was crossed to Ellis, the high-yielding, determinate MG IV soybean cultivar, having the functional *E1* allele.

**Table 2:** Allele combination of experimental and control lines used for field experiments in three different environments.

**Genotype category**	**Gene**					**Soybean lines produced in each production experiment**			
	***Dt1***	***Dt2***	***E1***	***E2***	***E3***	**Name**	**19GA**	**20TN**	**20MO**
Dt1_E1	*Dt1*	*dt2*	*E1*	*E2*	*E3*	G19-197	Incl.		
dt1_E1	*dt1* (R166W)	*dt2*	*E1*	*E2*	*E3*	Jake	Incl.	Incl.	
						Ellis		Incl.	
						S11-20242C		Incl.	
						G19-192	Incl.		
dt1-t1_E1	*dt1-t1* (R130K)	*dt2*	*E1*	*E2*	*E3*	KB17-16 #1	Incl.	Incl.	
						KB17-16 #2	Incl.	Incl.	
						KB17-16 #3	Incl.		
						KB17-16 #4	Incl.		
						KB17-16 #5	Incl.		
						KB17-16 #6	Incl.		
						KB17-16 #7	Incl.		
						KB17-16 #8	Incl.		
dt1-t2_E1	*dt1-t2* (R62S)	*dt2*	*E1*	*E2*	*E3*	KB17-17 #1	Incl.	Incl.	
						KB17-17 #2	Incl.	Incl.	
						KB17-17 #3	Incl.	Incl.	
						KB17-17 #4	Incl.	Incl.	
						KB17-17 #5	Incl.		
						KB17-17 #6	Incl.		
Dt2_E1	*Dt1*	*Dt2*	*E1*	*E2*	*E3*	KB17-7 #1	Incl.		
						KB17-7 #2		Incl.	
						KB17-7 #3		Incl.	
						KB17-7 #4		Incl.	
						KB17-7 #5		Incl.	
						KB17-8 #1	Incl.		
						KB17-8 #2		Incl.	
						KB17-8 #3		Incl.	
						KB17-8 #4		Incl.	
						KB17-8 #5		Incl.	
Dt1_e1	*Dt1*	*dt2*	*e1-as*	*E2*	*E3*	Clark	Incl.		
						Williams 82			Incl.
						Jack			Incl.
						LG04-6000			Incl.
						S07-5049			Incl.
						S13-10592		Incl.	
dt1_e1	*dt1* (R166W)	*dt2*	*e1-as*	*E2*	*E3*	L63-3016			Incl.
						L63-3297			Incl.
						L65-792			Incl.
						L72-1737			Incl.
dt1-t1_e1	*dt1-t1* (R130K)	*dt2*	*e1-as*	*E2*	*E3*	L91-8052		Incl.	Incl.
dt1-t2_e1	*dt1-t2* (R62S)	*dt2*	*e1-as*	*E2*	*E3*	L91-8060		Incl.	Incl.
Dt2_e1	*Dt1*	*Dt2*	*e1-as*	*E2*	*E3*	LG90-2550	Incl.		Incl.
						L62-1251			Incl.
						L73-811			Incl.
						KB18-14-1375			Incl
Total number of evaluated soybeans in each of the field experiments							21	20	14

*Each of the soybean lines followed by Incl. was included in each field experiment marked on the top of the column*.

The crosses for the experimental soybean populations were made in the 2017 soybean-growing season at the South Farm Research Center near Columbia, MO. The F_1_ plants of KB17-16 and KB17-17 were self-pollinated to produce F_2_ seeds in January 2018 in Upala, Costa Rica. The KB17-16 (378) and KB17-17 (338) F_2_ seeds were returned to Missouri and planted in the field in May 2018 and harvested individually to generate F_2:3_ seeds in November 2018; a bulk of three seeds from each line was used for DNA extraction using the DNeasy Plant Mini Kit (Qiagen, Inc., Valencia, CA) and genotyping assays for *dt1-t1* (R130K) and *dt1-t2* (R62S) alleles and functional *E1* alleles identified eight lines for KB17-16 and six lines for KB17-17 with the targeted genotype. Three F_2:3_ seeds from each selected line were sent to the winter nursery in Costa Rica to advance a single F_3_ plant per line, with F_3:4_ seeds returned to Missouri in 2019. Also, in the winter nursery in Costa Rica, the F_1_ plants of KB17-7 and KB17-8 were self-pollinated to produce F_2_ seeds in January 2018; the F_2_ plants were sampled with leaf presses on Whatman FTA cards and processed as DNA templates for genotyping assays for functional *Dt1* alleles, as well as *E1*. The plants selected with the targeted genotypes were then single plant threshed, and the F_2:3_ seeds were returned for field planting in Columbia, Missouri in May 2018, confirmed by genotype to have *Dt2* alleles, and harvested as F_2:4_ seeds in October 2018. The seeds from selected lines were then advanced a single generation in the winter nursery in Costa Rica and returned to Missouri as F_4:5_ seeds in April 2019.

### Allele-Specific Molecular Marker Assays

A total of five separate SimpleProbe assays were used to evaluate alleles at the *E1* maturity gene and the two genes regulating stem termination type, *Dt1* and *Dt2*, of the tested soybean lines. The SimpleProbe assays of *E1* alleles were conducted as described by Langewisch et al. (2017). For the *Dt1* gene, three separate SimpleProbe assays were developed to distinguish each of the missense mutations at the *Dt1* locus targeted for the population developments, *dt1-t2 (R62S), dt1-t1 (R130K)*, and *dt1 (R166W)*. The SimpleProbes used in each of the assays consisted of 5′-Fluorescein-SPC- GGA**C**CTCATATCACCACCCTCAAT-phosphate-3′ for *dt1-t2 (R62S)*, 5′-Fluorescein-SPC- TGGAGTAACACACTGT**C**TACGCTT-phosphate-3′ for *dt1-t1 (R130K)*, 5′-Fluorescein-SPC- TGCACAG**A**GGGAAACGGCT-phosphate-3′ for *dt1 (R166W)* allele. Each of the mutations is indicated by a bold font with an underline. Two different sets of PCR primers were designed to amplify *Dt1* exon 1 and exon 4 regions where the *dt1-t2* (R62S) allele, and *dt1-t1* (R130K) and *dt1* (R166W) mutations are located, respectively. For the *dt1-t2* (R62S) allele, genotyping reactions were performed with a 5:2 (forward to reverse) asymmetric mix of primers (Dt1upf1: 5′-CACACTCGATCTACCT-3′ at.5-μM final concentration, and Dt1in1r: 5′-ACATACCGTGTGACCATG-3′ at 0.2-μM final concentration). For *dt1-t1* (R130K) and *dt1* (R166W) alleles, genotyping reactions were performed with a 2:5 (forward to reverse) asymmetric mix of primers (Dt1in31f: 5′-CATGAGAGAGATCACTGAC-3′ at 0.2-μM final concentration, Dt1endr1: 5′-GCAAAACCAGCAGCTACTT-3′ at 0.5-μM final concentration). In terms of the *Dt2* gene, a SimpleProbe assay was developed for the associated nucleotide polymorphism associated with overexpression of the *Dt2* gene Glyma.18g273600 (Pos: 55642486 on Chromosome 18). The SimpleProbe consisted of 5′-Fluorescein-SPC- GTGCAGACTACCA**C**GCATGC -phosphate-3′. A set of PCR primers was designed to amplify the surrounding region of the SNP. Genotyping reactions were performed with a 5:2 (forward to reverse) asymmetric mix of primers (Dt2fa: 5′-CACAGGTTCGTAGTTATAG-3′ at 0.5-μM final concentration, and Dt2reva: 5′- CATAGGATACTAACCAACG-3′ at 0.2-μM final concentration). The SimpleProbes were designed using Roche Applied Science LightCycler Probe Design software 2.0 (version 1.0, February 2004), and the probes were ordered from Flourescentric, Inc. (Park City, UT).

Reactions for each of the genotyping assays were carried out in 20-μl total volume, containing a 5–50-ng DNA template, primers, 0.2-μM final concentration of SimpleProbe, a buffer [40-mM Tricine- KOH (pH 8.0) 16-mM KCl, 3.5-mM MgCl2, 3.75-μg.ml^−1^ BSA], 5% DMSO, 200-μM dNTPs, and 0.2-X Titanium Taq polymerase (BD Biosciences, Palo Alto, CA). Genotyping reactions were performed using a Lightcycler 480 II real-time PCR instrument (Roche Life Sciences, Indianapolis, IN), using the following PCR parameters: 95°C for 5 min, followed by 40 cycles of 95°C for 20 s, 60 °C for 20 s, 72°C for 20 s. The melting curves of each of the genotyping assay were as follows: a *dt1-t2* (R62S) assay—from 54 to 72°C (reference alleles produced a peak at 66°C, mutant alleles produced a peak at 58.5°C, and heterozygous samples produced both peaks); a *dt1-t1* (R130K) assay—from 50 to 80°C (reference alleles produced a peak at 64.5°C, mutant alleles produced a peak at 57°C, and heterozygous samples produced both peaks); a *dt1* (R166W) assay—from 50 to 70°C (reference alleles produced a peak at 63°C, mutant alleles produced a peak at 57°C, and heterozygous samples produced both peaks); a *Dt2* assay—from 54 to 72°C (reference alleles produced a peak at 67.8°C, mutant alleles produced a peak at 59.5°C, and heterozygous samples produced both peaks).

### Growth Conditions

For MG VI–VII environment, a set of 21 soybean lines, including 16 experimental and 5 control soybean lines under 7 genotype categories, was planted in 2019 at University of Georgia Iron Horse Plant Sciences Farm, Watkinsville, GA (19GA; Table 2). The field trial in GA consisted of a completely randomized design without replication. The lines were planted on June 5, 2019, at the seeding rate of 48 seeds per row. Individual plots consisted of two rows with a length of 1.83 m and row spacing of 76 cm. Artificial irrigation was applied as needed during the growth period.

In terms of the MG IV–V environment, a total of 20 soybean lines, including 14 experimental and 6 control soybean lines, were planted in 2020 at the East Tennessee Agriculture and Education Center, Knoxville, TN (20TN; Table 2). The soybean lines were planted on May 27, 2020 in a randomized complete block design with three replications. Individual plots consisted of two rows with the row length of 4.88 m; spacing, 76 cm apart. The seeding rate was 200 seeds per row. During the growing season, irrigation was applied depending on the field condition. A total of 20 plots with poor germination were not included in the data analysis.

For the MG III–IV environment, the 14 soybean lines with the *e1-as* background with different stem termination genotypes were planted in 2020 at the South Farm Research Center near Columbia, MO (20MO; Table 2). The field experiment was conducted based on a randomized complete block design with three replications. The soybean lines were planted on June 2, 2020 in 2.1-m rows with 0.6-m alleys and row spacing of 76 cm apart. The seeding rate was 50 seeds per row. No artificial irrigation was applied.

### Phenotype Measurements

Several agronomic traits were evaluated as parameters to assess potential agronomic merits depending on the plant architecture genes in each of the three production environments. For the field experiment in 2019 in Georgia (19GA), five morphological characteristics, plant height, number of nodes, stem diameter, lodging, and number of pods were measured at maturity. Plant height, number of nodes, and stem diameter were measured from five randomly selected individual plants from each plot, and lodging was scored per plot. Plant height was measured as the distance from the soil surface at the base of the plants to the stem apex in centimeters. The number of nodes was counted from the main stem of each plant, and the diameters at the first, middle, and last internodes of each plant were measured, and then the mean of the three diameters was calculated as overall thickness of each stem. Lodging was subjectively scored per plot on a scale of 1 (all the plants were erect) to 5 (almost all the plants were prostrate). Number of pods was counted at an apical stem of each plant.

For the Tennessee (20TN) production environment, a total of seven agronomic traits, including the five morphological parameters assessed in 19 GA plus two other parameters, days to maturity (DTM), and raceme length, were evaluated at maturity. Lodging and DTM were evaluated per plot, while the other morphological characteristics were measured on 10 individual plants from each plot. The plant height, the number of nodes, stem diameter, lodging, and number of pods were measured in the same manner as in 19 GA. For DTM, it was measured as the number of days from planting to the date when the soybean plants in a plot reached the R8 stage when 95% of the pods reached their mature color. Length of terminal raceme was recorded in centimeters, and the number of branches was counted from the main stem of each plant.

In the field trial at Missouri in 2020 (20MO), a total of nine agronomic traits were recorded, including the seven parameters measured in 19TN and two additional traits, days to flowering (DTF), and number of branches. Lodging, DTM, and DTF were evaluated per plot, and the other characteristics were evaluated from five individual plants per plot. For the DTF, it was scored as the number of days from planting to the date when more than three plants had opened flowers within each plot. The number of branches was counted from the main stem of each plant. The other seven agronomic traits were measured in the same manner as in 19GA and 20TN.

### Data Analysis

For the phenotype data collected in MG VI-VII environment in the US, the differences in agronomic traits depending on genotype combinations were analyzed using descriptive statistics since the experimental design used was completely randomized design without replication. In terms of the phenotype data collected in MG IV-V and MG III-IV environments, the statistical differences in agronomic traits depending on genotype combinations were tested using two separate methods. For lodging scores, the data were analyzed using the non-parametric Kruskal–Wallis test using PROC NPAR1WAY in SAS version 9.4 (SAS Institute Inc., Cary, NC, 2013), and significant differences between each genotype category were examined using the Dwass, Steel, Critchlow-Fligner (DSCF) multiple comparison analysis. Otherwise, statistical differences were tested using PROC GLM in SAS version 9.4 (SAS Institute Inc., Cary, NC, 2013). Means were separated by Fisher's least significant difference (LSD) procedure at the *p* = 0.05 probability level.

## Results

### The Classical *dt1-t* Alleles Responsible for Tall Determinate Stem Termination Type Are Distinct Missense Mutations in *dt1*

Since the gene controlling the tall determinate stem type was found to be allelic to *dt1* (Thompson et al., 1997), we first examined the DNA sequence of the coding region of *Dt1* (Glyma.19g194300; 45183357 – 45185175 Wm82.a2.v1) in the classical tall determinate soybean cultivars, Peking and Soysota, and their isolines L91-8060 and L91-8052 (Table 1; Supplementary Table 1). Glyma.19g194300 is an 1819 bp gene with four exons that encode a 173 amino acid protein, alternatively termed as *GmTFL1* (https://www.ncbi.nlm.nih.gov/sra) (Tian et al., 2010). In the coding region of *Dt1*, six missense alleles—R62S, L67Q, P113L, R130K, H141R, and R166W—have been identified in the search for recessive variants (Liu et al., 2010; Tian et al., 2010; Yue et al., 2021). In the Williams 82 Assembly 2 Genomic Sequence (Wm82.a2), the *Dt1* gene, Glyma.19g194300, is oriented on the opposite strand. For the tall determinate cultivars Peking and Soysota, we utilized resequencing data from Zhou et al. (2015) and analyzed nucleotide polymorphisms at the *Dt1* locus. For the two isolines, a primer set specific for *Dt1* gene and PCR amplification was developed, and the sequence of the gene was confirmed by PCR analysis and Sanger sequencing of PCR fragments.

The classical tall determinate soybean line Peking and its isoline, L91-8060, contained a guanine to thymine causative SNP on Chromosome 19 at position 45,184,804 (Wm82.a2.v1), g186t in coding sequence. It creates a missense mutation, resulting in a change from arginine to serine at amino acid position 62 (R62S) in the protein amino acid sequence. The other tall determinate soybean line Soysota and its isoline, L91-8052, contained a guanine to adenine causative SNP on Chromosome 19 at position 45,183,808 (Wm82.a2.v1), g389a in coding sequence. It creates a missense mutation, resulting in a change from arginine to lysine at position 130 (R130K) in the protein amino acid sequence. The tall determinate stem type alleles will herein be referred to as *dt1-t1* and *dt1-t2*, specifying the R130K and R62S missense alleles, respectively; the standard determinate stem type representing the arginine to tryptophan at position 166 (R166W) missense alleles will be referred to herein as *dt1*.

### High Disparity in Distinguishing Stem Termination Types in Soybean Accessions With Tall Determinate Alleles

In an attempt to address how stem termination types of soybean accessions with the tall determinate alleles that have been phenotypically scored, as well as to broaden our understanding of the relationships between the known genotypes in stem termination and the actual phenotype displayed, we analyzed publicly available genome resequence data, along with phenotypic measurement data of the evaluated soybean lines. A total of 528 soybean accessions, which have both phenotype and resequencing data, were examined. The genotype of each accession was designated depending on the allele combinations at the *Dt1* and *Dt2* loci (Table 1). The accessions were grouped based on their genetic stem termination type based on the *Dt1* and *Dt2* genotype and then identified by their observed stem termination phenotype (determinate, semi-determinate, or indeterminate).

**Figure 1:**
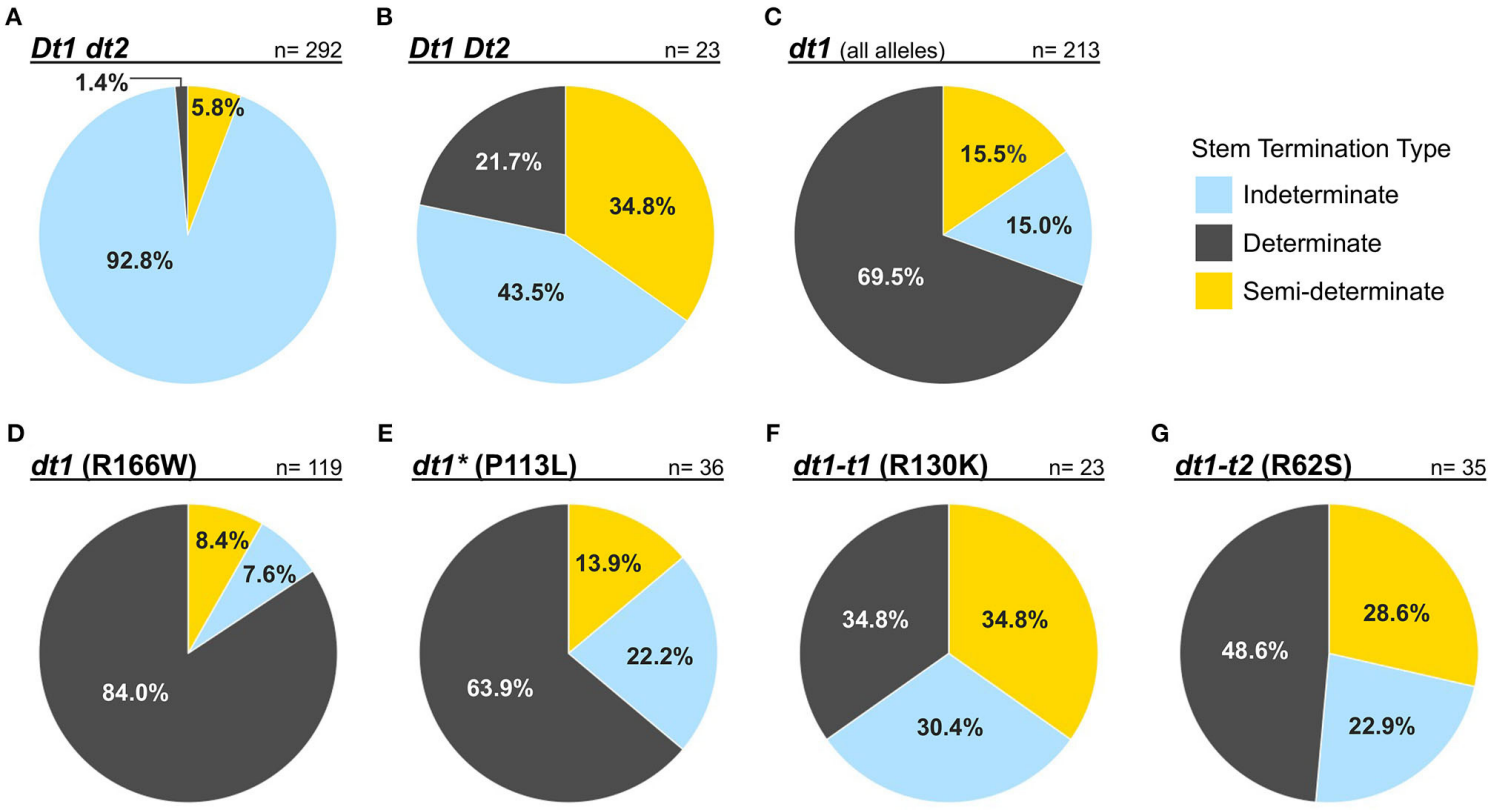
Pie charts showing the rates of concordance between publicly available phenotypes and genotypes for stem termination types of 528 *G. max* accessions. The phenotype data were downloaded from the GRIN database. Each of the evaluated phenotypes was highlighted by colors—blue: indeterminate; yellow: semi-determinate; gray: determinate. Genotype categories about which each of the pie charts illustrates were written in bold and italic characters above of lines drawn over each chart. Percentages written in pie charts represent rates on how often people evaluated the stem termination types of soybean accessions within each genotype category. Digits followed by n = represented numbers of the *G. max* accessions within each genotype category. **(A–C)** Illustrate how stem termination types of soybean accessions with each genotype combination for the *Dt1* and *Dt2* genes that have been phenotypically scored. **(D–G)** Showed variations in scored phenotypes depending on the presence of each of the four missense mutations at the *dt1* locus: *dt1* (R166W) represents the standard determinate stem type, *dt1-t1* (R130K), and *dt1-t2* (R62S) represent the tall determinate stem type alleles, and dt1* (P113L) represents another frequently found missense mutation at the *dt1* locus, which is only mentioned in the analysis on disparity.

The rates of concordance between the observed stem termination type phenotypes from the GRIN database and the expected phenotypes based on the genetic allele combination of stem growth habit genes for the *G. max* accessions are shown in Figure 1. When soybean accessions have the indeterminate *Dt1dt2* genotype, observers correctly assigned the indeterminate stem termination type with a rate of 92.8% (Figure 1A). A high rate of discrepancy was observed for the genetically semi-determinate *Dt1Dt2* genotype, where only about 35% of those accessions were scored with a semi-determinate phenotype (Figure 1B). Despite the distinct phenotypic characteristics of the determinate stem type, not quite 70% of the genetically determinate accessions were scored as determinate when evaluating soybeans with any of the missense *dt1* alleles (Figure 1C). The group of soybean accessions with missense *dt1* alleles was further subdivided into four genotype categories, depending on the presence of one of the four missense mutations (Figures 1D–G). Interestingly, the concordance between different *dt1* alleles and observed phenotypes was specific to the allele status. When *G. max* accessions have the *dt1* (R166W) allele, 84% of these were classified as a determinate type (Figure 1D). In the case of dt1^*^ (P113L), about 64% of the accessions were scored determinate (Figure 1E). Notably, when either the *dt1-t1* (R130K) or *dt1-t2* (R62S) alleles were present, the soybean accessions were more frequently evaluated as indeterminate or semi-determinate rather than the determinate type, with only a minority 34.8 or 48.6%, respectively, scored as determinate. This disparity, which was also observed for the genetically semi-determinate *Dt1 Dt2* genotype, suggests that soybeans having *dt1-t1* (R130K) or *dt1-t2* (R62S) alleles are likely to have intermediate stem characteristics, similar to the semi-determinate genotype allele combination. The overall discrepancy in phenotyping stem termination type implies that phenotypic evaluation itself is not enough for efficient utilization of the traits. Additional strategies, such as marker-assisted selection or development of sophisticated criteria in phenotyping, are required utilization in soybean breeding and improvement.

### Effects of Plant Architecture Genes on Agronomic Characteristics in an MG VI–VII Environment

In order to evaluate potential agronomic merits of the tall determinate alleles and other plant architecture genes in MG VI–VII environments, experimental and control soybean lines having each of seven different genotype combinations for stem architecture and maturity genes were produced in a field experiment at Athens, GA (19GA), for evaluation of their morphological characteristics. The genotype categories for the soybean lines were assigned based on the allele status of the two genes for stem termination type, *Dt1* and *Dt2*, and one gene for maturity, *E1* (Table 2). Among the seven genotype categories, *dt1* (R166W) with *E1* (dt1_E1) is the most predominantly found from commercial soybean cultivars grown in the southern US environments. Five agronomic traits—plant height, number of main stem nodes, internode diameter, lodging, and number of pods at stem tip—were measured as parameters for evaluating agronomic performance of each genotype category under the production environment.

**Figure 2:**
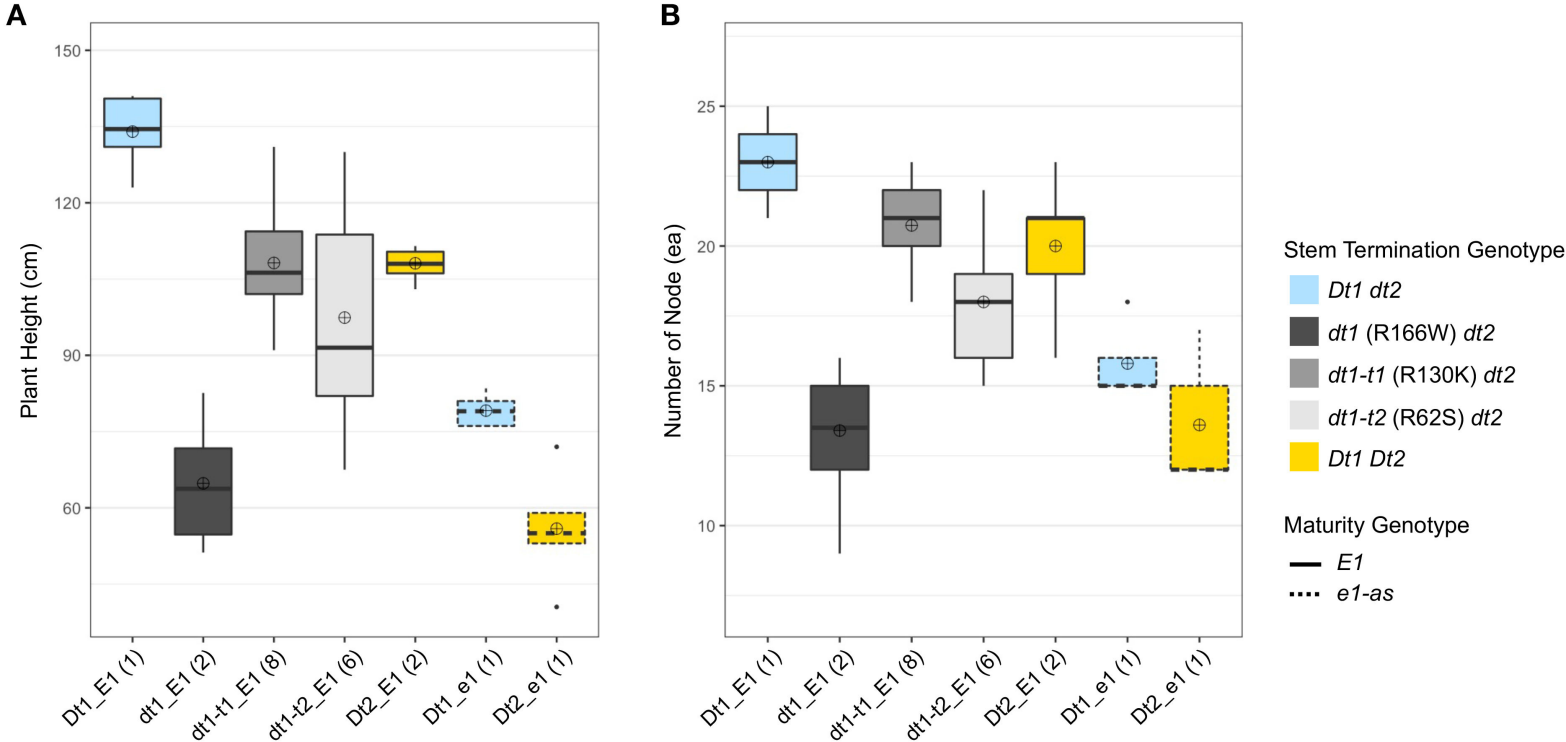
Boxplots of plant height and number of nodes depending on genotype categories examined in 2019 at Athens, GA (19GA). A total of 21 soybean lines with each allele combination for genes related to stem termination type and maturity were evaluated. The colors of each box were designated based on the allele status of the two genes conferring stem termination type, *Dt1* and *Dt2*. The outlines of each box were designated based on the allele status of the *E1* maturity gene. Sixteen soybean lines under three genotype categories, dt1-t1_E1, dt1-t2_E1, and Dt2_E1, were experimental lines from KB17-16 (F_3:4_), KB17-17 (F_3:4_), and either KB17-7 or KB17-8 RIL populations (F_4:5_), respectively. The other five soybean lines were control cultivars. Each genotype category has an unequal number of soybean lines, and the number of lines was written in parentheses beside each genotype category. The plant height and the number of nodes were measured from five individual plants per line. ⊕ in each box represents mean values of each genotype category. **(A)** Displays the distribution of final plant height at maturity in cm. **(B)** Shows the distribution of number of nodes counted on the main stem at maturity.

The number of pod-bearing nodes achieved due to plant height, architecture, and plant density is directly associated with yield potential of soybeans (Vogel et al., 2021). In the functional *E1* background, the soybean lines with *dt1* alleles (dt1_E1) had the shortest plant heights with an average plant height of 64.8 cm compared to other soybean lines under different genotype categories (Figure 2A). Even the control line, which carries the early flowering *e1-as* gene and indeterminate alleles of *Dt1*, was taller (Dt1_e1; 79.1 cm) than the dt1_E1 lines with *dt1* and late flowering *E1* alleles (Figure 2A). Notably, in the functional *E1* background, the soybean lines having either *dt1t-1* or *dt1t-2* missense mutations (dt1-t1_E1 and dt1-t2_E1) were about 1.7 or 1.5 times, respectively, taller than those with *dt1* alleles, expanding the earlier characterization of tall determinate genetic types in the *e1-as* background (Thompson et al., 1997).

Interestingly, the lines with *dt1-t1* (R130K) alleles and E1 (dt1-t1_E1) had similar plant heights compared to those having the category Dt2_E1, which showed intermediate plant heights typically expected from the semi-determinate stem termination type (Ping et al., 2014). The ranking of plant heights in the *E1* background had the *Dt1* line as the tallest, *dt1* lines as the shortest, and the other three categories as intermediate in height with dt1-t1_E1 and Dt2_E1 category lines having similar means, but, within the intermediate set, the dt1-t2_E1 lines were shorter (Figure 2A).

In the case of the number of nodes, soybean lines with taller plants tended to have more nodes, and the pattern was similar between plant height and number of nodes (Figure 2). In the functional *E1* background, soybean lines with *dt1* alleles had the fewest nodes with an average number of nodes of 14.3 for dt1_E1 compared to those with other stem termination genotypes (Figure 2B). The early flowering soybean lines having Dt1_e1 or Dt2_e1 genotypes in the *e1-as* background produced a similar small number of nodes like those with dt1_E1 alleles. In the functional *E1* maturity gene background, genotype Dt1_E1 had the highest number of nodes with an average number of nodes of 23, and lines with dt1-t1_E1 or Dt2_E1 stem termination genotypes had the second most nodes with an average number of nodes of 20.7 or 20.0, respectively. Soybeans with dt1-t2_E1 alleles also had a higher number of nodes, averaging about 5 more nodes than those with dt1_E1 alleles. The ranking of number of nodes in the *E1* background had the Dt1_E1 line with the most, dt1_E1 lines with the fewest, and the other three categories as intermediate (Figure 2B).

The soybean plant architecture parameters, internode diameter, number of pods at the apical stem, raceme length, and lodging score are also associated with loss of yield potential (Vogel et al., 2021). For considering thickness of the whole parts of a main stem in each soybean plant, the mean of the diameters measured at the first, middle, and last internodes were used as a scale of the stem diameter in this study. There were differences in the lodging components, depending on plant architecture genotype categories for 19 GA (Table 3). In the functional *E1* background, compared to the typical determinate dt1_E1 stem termination genotype in this environment, most soybean lines, which exhibited taller plant heights and more nodes, had stem thickness that was not significantly different from the dt1_E1 genotypes with an average stem diameter of 6.4 mm. Only the soybeans with semi-determinate Dt2_E1 alleles were thinner than those with dt1_E1 alleles. In the functional *E1* maturity gene background, soybean lines with either functional *Dt1* or one of the three missense mutations at the *dt1* locus (Dt1_E1, dt1-t1_E1, dt1-t2_E1, or dt1_E1) showed an average stem diameter, ranging from 6.1 to 6.8 mm; the variations depending on the stem termination allele at the *dt1* locus were not significant for the stem diameter. There were differences in lodging scores for soybean lines, depending on the genotype category for 19GA (Table 4). The lodging scores of the soybeans with either of the two tall determinate alleles (dt1-t1_E1, dt1-t2_E1) were higher than the average lodging score of 1 for those with dt1_E1 alleles. There was substantial variation for lodging scores for the lines with tall determinate alleles and, to a lesser degree, also, for semi-determinate lines (Table 4).

**Table 3:** Mean values of the internode diameter, number of pods at apical stems, raceme length, and maturity for soybeans having different genotype combinations in field experiments at two different southern environments in 2019 and 2020 (19GA and 20TN).

**Genotype category**	**Gene**			**19GA**			**20TN**				
	***Dt1***	***Dt2***	***E1***	***n***	**SD**	**Pod**	***n***	**SD**	**Pod**	**RL**	**DTM**
Dt1_E1	*Dt1*	*dt2*	*E1*	1	6.2 ± 0.6	1.2 ± 0.8					
dt1_E1	*dt1* (R166W)	*dt2*	*E1*	2	6.4 ± 1.0	8.6 ± 2.7	3	5.2 ± 1.3 b	7.7 ± 3.1 ab	2.9 ± 1.5 a	140.1 ± 3.1 a
dt1-t1_E1	*dt1-t1* (R130K)	*dt2*	*E1*	8	6.8 ± 1.2	6.4 ± 2.7	2	6.9 ± 1.7 a	6.9 ± 3.9 bc	1.8 ± 1.2 b	137.7 ± 1.3 b
dt1-t2_E1	*dt1-t2* (R62S)	*dt2*	*E1*	6	6.1 ± 0.8	5.1 ± 3.2	4	6.8 ± 1.6 a	8.7 ± 3.4 a	3.0 ± 1.4 a	140.1 ± 3.7 a
Dt2_E1	*Dt1*	*Dt2*	*E1*	2	5.5 ± 1.5	4.1 ± 2.2	8	5.2 ± 1.3 b	6.2 ± 2.5 c	1.0 ± 0.6 c	138.5 ± 3.1 b
Dt1_e1	*Dt1*	*dt2*	*e1-as*	1	5.2 ± 0.8	1.2 ± 0.8	1	4.9 ± 1.0 b	3.1 ± 1.7 e	0.2 ± 0.4 d	138.5 ± 0.5 b
dt1-t1_e1	*dt1-t1* (R130K)	*dt2*	*e1-as*				1	4.7 ± 1.0 b	4.6 ± 2.1 d	1.6 ± 0.7 b	131.3 ± 1.3 d
dt1-t2_e1	*dt1-t2* (R62S)	*dt2*	*e1-as*				1	4.7 ± 0.9 b	5.7 ± 2.2 cd	1.7 ± 0.7 b	132.5 ± 0.5 c
Dt2_e1	*Dt1*	*Dt2*	*e1-as*	1	3.1 ± 0.9	2.4 ± 1.5					
			LSD (0.05)		0.8	2.0		0.6	1.4	0.5	0.9

*Each genotype category has an unequal number of soybean lines and n represents number of lines within each of the genotype categories. SD represents mean of stem diameters (mm) measured at first, middle, and last internode at maturity. Pod represents number of pods at apical stems, and RL represents raceme length in cm. DTM was measured by plot. For other traits, total five individual plants per line for 19GA, and maximum thirty individual plants per line for 20TN (10 plants by 3 reps; but data from 20 plots with poor germination were not included for the data analysis) were sampled for phenotyping. Mean values of each trait were obtained by averaging the means of lines within each genotype category, and standard deviations are listed after the mean values (±). Means followed by the same letter were not significantly different from each other at 95% confidence with Fisher's least significant difference (LSD) procedure*.

**Table 4:** Genotypes and lodging scores of soybeans lines having allele combinations for stem termination type and maturity from field experiments at three different environments in 2019 and 2020, 19GA, 20TN, and 20MO.

**Genotype category**	**Gene**			**Lodging**										
	***Dt1***	***Dt2***	***E1***	**19GA**			**20TN**				**20MO**			
				***n***	** $\bar{x}\pm \sigma$ **	** $\tilde{x}$ **	***n***	** $\bar{x}\pm \sigma$ **	** $\tilde{x}$ **		***n***	** $\bar{x}\pm \sigma$ **	** $\tilde{x}$ **	
Dt1_E1	*Dt1*	*dt2*	*E1*	1	1.0 ± 0.0	1.0								
dt1_E1	*dt1* (R166W)	*dt2*	*E1*	2	1.0 ± 0.0	1.0	3	2.2 ± 0.6	2.0	ab				
dt1-t1_E1	*dt1-t1* (R130K)	*dt2*	*E1*	8	2.8 ± 1.0	3.3	2	2.4 ± 0.3	2.5	a				
dt1-t2_E1	*dt1-t2* (R62S)	*dt2*	*E1*	6	1.6 ± 0.9	1.0	4	2.4 ± 0.3	2.5	a				
Dt2_E1	*Dt1*	*Dt2*	*E1*	2	3.0 ± 0.5	3.0	8	2.2 ± 0.3	2.0	b				
Dt1_e1	*Dt1*	*dt2*	*e1-as*	1	1.0 ± 0.0	1.0	1	2.5 ± 0.0	2.5	a				
dt1-t1_e1	*dt1-t1* (R130K)	*dt2*	*e1-as*				1	1.7 ± 0.2	1.5	c				
dt1-t2_e1	*dt1-t2* (R62S)	*dt2*	*e1-as*				1	1.5 ± 0.0	1.5	c				
Dt2_e1	*Dt1*	*Dt2*	*e1-as*	1	1.0 ± 0.0	1.0								
Dt1_e1	*Dt1*	*dt2*	*e1-as*								4	1.3 ± 0.2	1.5	a
dt1_e1	*dt1* (R166W)	*dt2*	*e1-as*								4	1.1 ± 0.3	1.0	b
dt1-t1_e1	*dt1-t1* (R130K)	*dt2*	*e1-as*								1	1.0 ± 0.0	1.0	a
dt1-t2_e1	*dt1-t2* (R62S)	*dt2*	*e1-as*								1	1.2 ± 0.2	1.0	a
Dt2_e1	*Dt1*	*Dt2*	*e1-as*								4	1.0 ± 0.1	1.0	b

*The lodging score was evaluated by plot. The lodging was scored at maturity on a scale of one (all the plants were erect) to five (almost all the plants were prostrate). Each genotype category has an unequal number of soybean lines, and n represents number of lines within each of the genotype categories, $\bar{x}$ ± σ refers to the mean ± standard deviation, and $\tilde{x}$ refers to the median of the samples within the genotype categories. Mean values of each trait were obtained by averaging the means of lines within each genotype category; the genotypes followed by the same letter indicate the genotypes are not significantly different from each other at 95% confidence with the Dwass, Steel, Critchlow–Fligner multiple comparison analysis*.

### Effects of Plant Architecture Genes on Agronomic Characteristics in an MG IV–V Environment

The potential agronomic merits of the plant architecture genes were further evaluated in a field experiment (20TN) in Knoxville TN, equivalent to MG IV-V (Table 2). Like the previous field trial in 19GA, the genotype categories of each soybean line were classified based on the genotype at the two loci for stem termination type, *Dt1* and *Dt2*, and one maturity gene, *E1* (Table 2). The seven genotype categories tested in 20TN were slightly different from those tested in 19GA. For evaluating the potential agronomic merits of each of the genotype categories in the 20TN production environment, the agronomic traits plant height, number of nodes in the main stem, stem diameter (SD), number of pods at the apical stem, raceme length, days to maturity, and lodging score were measured as parameters.

**Figure 3:**
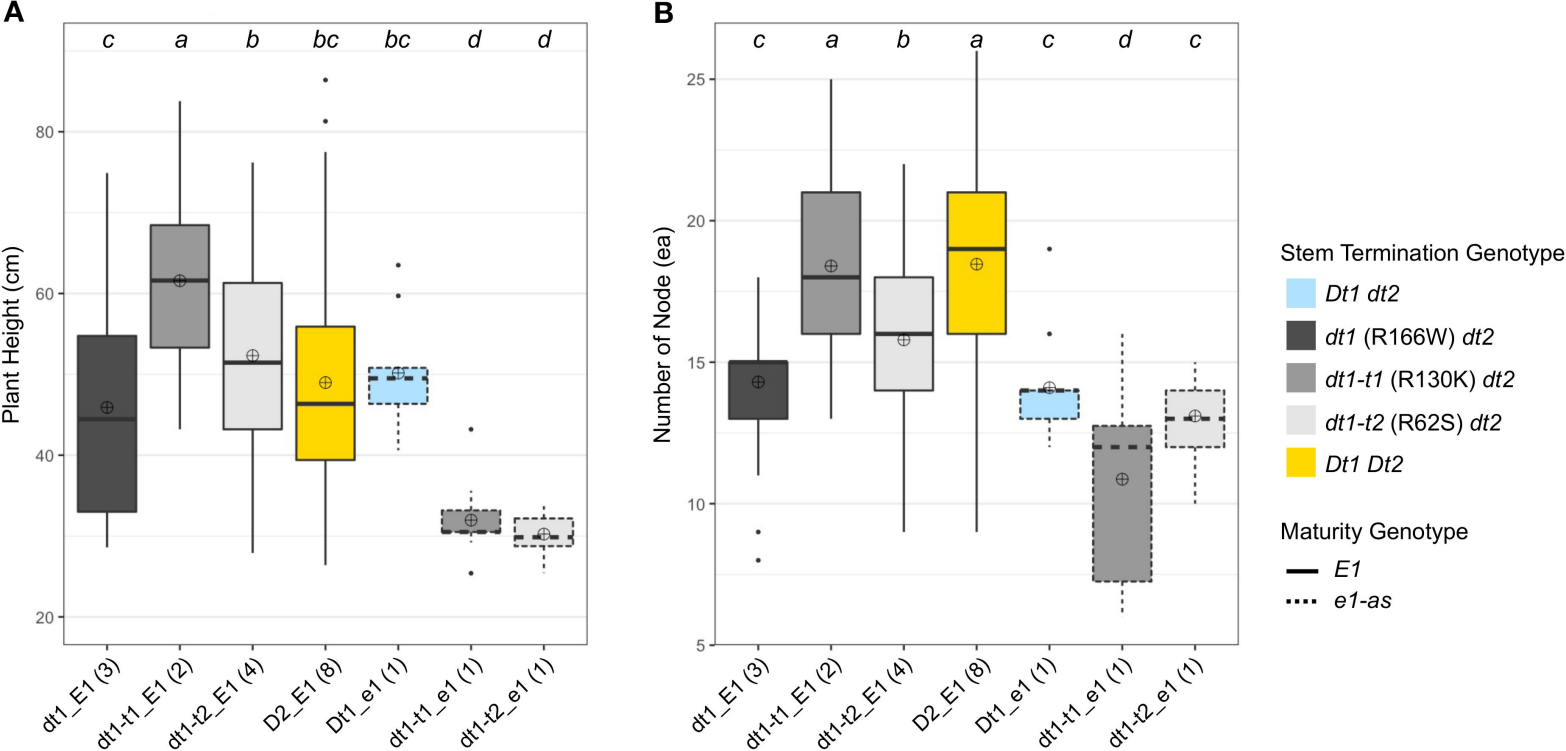
Boxplots of plant height and number of nodes depending on genotype categories examined in 2020 at Knoxville, TN (20TN). A total of 20 soybean lines with each allele combination for genes related to stem termination type and maturity were evaluated. The colors of each box were designated based on the allele status of the two genes, conferring stem termination type, *Dt1* and *Dt2*. The outlines of each box were designated based on the allele status of the *E1* maturity gene. Fourteen soybean lines under three genotype categories, dt1-t1_E1, dt1-t2_E1, and Dt2_E1, were experimental lines from KB17-16 (F_3:5_), KB17-17 (F_3:5_), and either KB17-7 or KB17-8 RIL populations (F_4:6_), respectively. The other six soybean lines were control cultivars. Each genotype category has an unequal number of soybean lines, and the number of lines was written in parentheses beside each genotype category. The plant height and the number of nodes were measured from ten individual plants per plot. ⊕ in each box represents mean values of each genotype category. The genotype categories marked with a common lower-case letter indicate that the means are not significantly different (*p* = 0.05) according to Fisher's Least Significant Difference (LSD) procedure. **(A)** Displays the distribution of final plant height at maturity in cm. **(B)** Shows the distribution of number of nodes counted on the main stem at maturity.

The planting date in 20TN was later than in 19GA, and the plant heights of experimental and control lines grown in 20TN were overall shorter (ranging from 25.4 cm to 86.4 cm) than those grown in 19GA (Figure 3A). As observed in the previous field trial in 19GA, the lines with dt1_E1 alleles had the shortest plant heights with an average plant height of 45.9 cm among all the tested genotype categories in the functional *E1* maturity gene background (Figure 3A). The plant heights of soybean lines with the functional *E1* and the two tall determinate alleles, either dt1-t1_E1or dt1-t2*_*E1, were significantly increased with average plant heights of 61.6 cm and 52.3 cm, respectively, compared to those with the dt1-t1_E1 alleles in this production environment, which aligns with the observations in the field experiment in 19GA. Unlike the plant heights of soybean lines under the Dt2_E1 genotype category grown in 19GA, which showed statistically equivalent plant heights with those under the dt1-t1_E1 category, the Dt2_E1 lines in the 20TN production environment had significantly shorter plant heights with an average plant height of 49. cm compared to the dt1-t1_E1 lines. The ranking of plant heights in the *E1* background had the dt1-t1_E1 lines as the tallest, dt1_E1 lines as the shortest, although statistically equivalent to the Dt2_E1 lines, and the dt1-t2*_*E1 lines as intermediate in height between dt1-t1_E1 and dt1_E1 lines.

The ranking pattern of the number of nodes in this production environment differed from the 20TN environment plant heights (Figure 3). In the functional *E1* background, the number of nodes of soybean lines with either dt1-t1_E1 or dt1-t2*_*E1 alleles was significantly increased from those with dt1-t1_E1 alleles in this production environment, which also expands the earlier characterization of tall determinate genetic types originally studied in the *e1-as* background (Thompson et al., 1997). The ranking of number of nodes in the *E1* background had the Dt2_E1 and dt1-t1_E1 lines with the most (18.5 and 18.4 ea, respectively), with no significant difference between these two stem termination types; dt1_E1 lines had the fewest nodes (14.3 ea), and dt1-t2*_*E1 had an intermediate number of nodes (15.8 ea) (Figure 2B).

Similar to the observations in the 19GA production environment, significant differences in lodging components, stem diameter, and lodging score were observed, depending on the tested genotype categories (Tables 3, 4). In the functional *E1* background, soybean lines with the tall determinate alleles, either dt1-t1_E1or dt1-t2*_*E1, showed the thickest stems (6.9 or 6.8 mm, respectively), with no significant differences. The other five genotype categories had statistically equivalent stem diameters, ranging from 4.7 to 5.2 mm, regardless of the allele status at the *E1* maturity gene. In terms of the lodging score for 20TN, the lines in the functional *E1* background with different stem termination types were statistically different, but the actual mean value of the lodging score by genotype category was phenotypically indistinguishable (Table 4). The soybean lines with *e1-as* alleles showed mean lodging scores, ranging from 1.5 to 1.7 mm, overall better in lodging scores than those with *E1* alleles.

### Effects of Plant Architecture Genes on Agronomic Characteristics in an MG III–IV Environment

The differences in agronomic traits related to yield potential of soybean depending on plant architecture genes were also evaluated in Columbia, MO, which is an MG III–IV environment (20MO). A total of 14 lines, including eight Clark isolines and six control lines, were evaluated in the 20MO field experiment (Table 2). Each of the 14 lines was grouped into five genotype categories, depending on the allele combinations of the two genes regulating stem termination type, *Dt1* and *Dt2*. In terms of the *E1* maturity gene, all the tested soybean lines in 20MO had the *e1-as* allele, which is the maturity gene used in this production environment. Among the five tested genotype categories, Dt1_e1 is the most predominantly found in the Midwest environment in the US (Bernard, 1972; Tian et al., 2010; Liu et al., 2015). A total of nine agronomic traits, plant height, number of nodes, stem diameter, lodging score, days to flowering (DTF), days to maturity (DTM), number of pods at stem tips, raceme length, and number of branches were measured as parameters for evaluating agronomic performance, depending on the allele combination in the 20MO environment.

**Figure 4:**
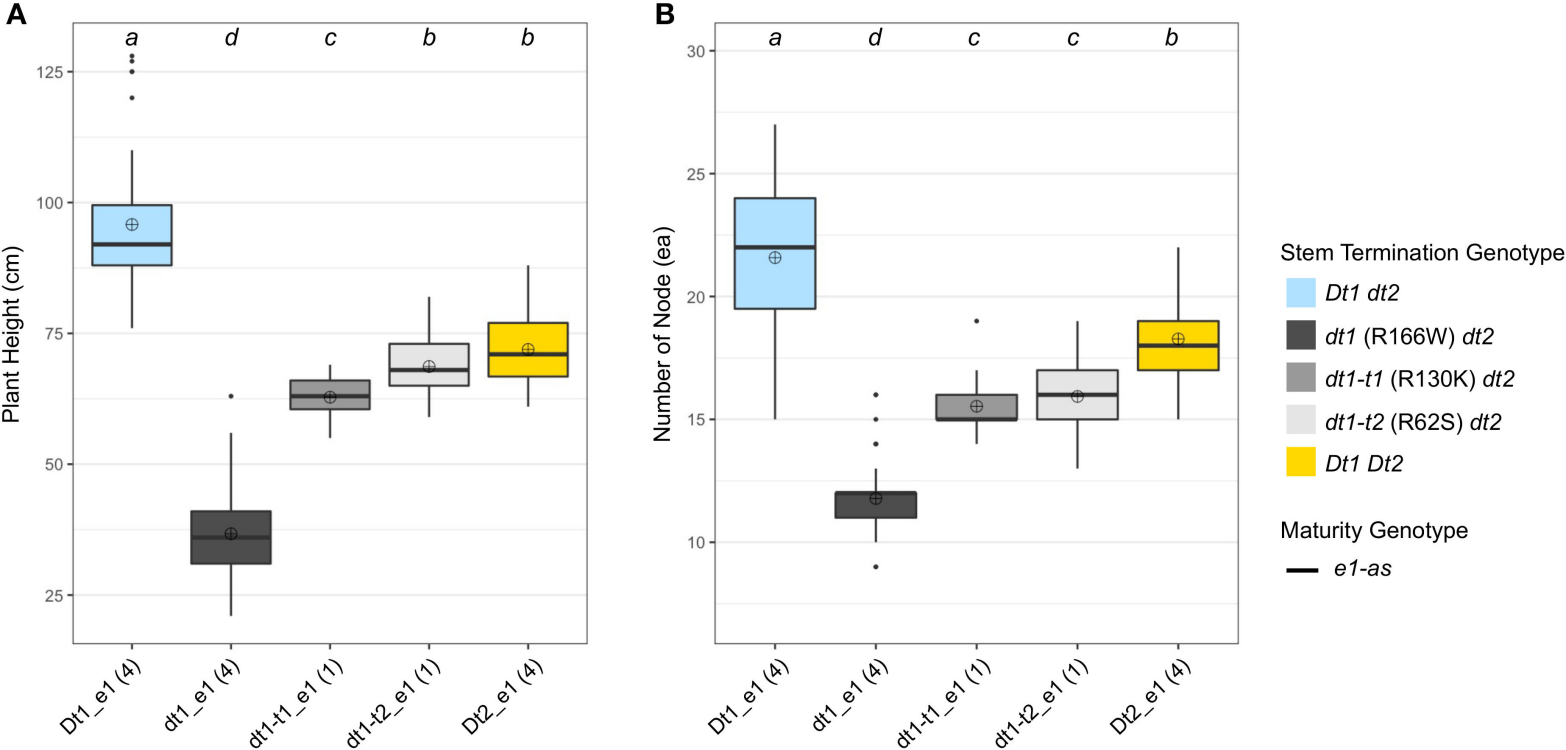
Boxplots of plant height and number of nodes depending on genotype categories examined in 2020 in Columbia, MO (20MO). A total of 14 soybean lines with each allele combination for genes related to stem termination type and maturity were evaluated. The colors of each box were designated based on the allele status of the two genes, conferring stem termination type, *Dt1* and *Dt2*. All the experimental and control soybean lines had *e1-as* maturity alleles. Each genotype category has an unequal number of soybean lines and the number of lines was written in parentheses below each genotype category. The plant height and the number of nodes were measured from 15 individual plants per plot. ⊕ in each box represents mean values of each genotype category. The genotype categories marked with a common lower-case letter indicate that the means are not significantly different (*p* = 0.05) according to Fisher's Least Significant Difference (LSD) procedure. **(A)** Displays the distribution of final plant height at maturity in cm. **(B)** Shows the distribution of number of nodes counted from a main stem at maturity.

In terms of plant height, the soybean lines with the functional *Dt1* allele had the tallest plants, with an average plant height of 95.8 cm among the five different stem termination types tested in this environment; the plant height of soybean lines having *dt1* alleles had the shortest plant height, with an average of 36.7 cm (Figure 4A). Notably, in the presence of either of the two tall determinate alleles, *dt1-t1* or *dt1-t2*, the plant heights were increased about 1.7 or 1.9 times, respectively, compared to those with the *dt1* alleles. In the case of the soybean lines with *dt1-t2* alleles having a mean plant height of 68.7 cm, they had statistically equivalent plant heights to the soybean lines having *Dt2* with a mean plant height of 71.9 cm. In summary, the ranking of plant height in the *e1-as* background had the *Dt1* lines as the tallest, *dt1* lines as the shortest, and the three categories as intermediate in height, with only small significant differences in *dt1-t1* compared to *dt1-t2* and *Dt2* (Figure 4A). The 20MO environment results with *dt1-t1* and *dt1-t2* alleles in the *e1-as* background were consistent with the original characterization of tall determinate genetic types (Thompson et al., 1997).

For the number of nodes of the tested soybean lines in the 20MO experiment, there was a tendency for the taller plants to have more nodes, and the pattern of ranking was remarkably similar with that of plant height (Figure 4). In the *e1-as* background, soybean lines with the *Dt1* allele had the most nodes with an average number of nodes of 21.6, while those with *dt1* had the fewest nodes with an average number of nodes of 11.8 (Figure 4B). Consistent with the previous observation by Thompson et al. (1997), the soybean lines with either *dt1-t1* or *dt1-t2* alleles had about 5 more nodes compared to those with *dt1* alleles. In terms of soybean lines with *Dt2* alleles, these had about 7 more nodes compared to those with *dt1* alleles. Therefore, the ranking of number of nodes in the *e1-as* background had the *Dt1* lines with the most, *dt1* lines with the fewest, and the other three categories as intermediate in number of nodes, while significant differences were observed between *Dt2* and the tall determinate category lines.

There were significant differences in the other agronomic traits measured in the 20MO production environment (Table 5). For the stem diameter, the soybean lines having the missense alleles, *dt1, dt1-t1*, and *dt1-t2* had the thickest stems without significant differences between the alleles (Table 5). The stem diameter of the soybean lines having *dt1* alleles was about 0.8 to 1.6 mm thicker compared to those having either the *Dt1* or *Dt2* alleles, respectively. The mean lodging scores of all the tested genotype categories were at or below 1.3, which reflects very low lodging for lines in any of the categories (Table 4). As for the flowering time, the *Dt2* lines were significantly earlier flowering compared to other stem termination types with an average DTF of 42.1 days (Table 5). In comparison of DTF among soybean lines with either functional *Dt1* or one of the missense *dt1 or dt1-t* alleles, statistically significant differences were observed, although the actual differences were <4 days at the most. In terms of maturity, soybean lines with *Dt1* allele matured significantly later compared to other genotype categories with an average DTM of 4.7 days later than the next closest genotype category (Table 5). The soybean lines with *Dt2* alleles were the earliest to mature, with the difference in DTM between *Dt2* and *Dt1* lines of about 8 days. The DTM of soybean lines with missense *dt1* or *dt1-t* alleles was intermediate between those of soybean lines with either *Dt1* or *Dt2*. In the comparison in DTM among the soybean lines with *dt1* or either of the *dt1-t* alleles, the differences were <2 days at the most. The average number of branches of all the tested genotype categories was <2, and there were no significant differences in number of branches depending on the genotype categories (Table 5). In terms of morphological characteristics at the stem tip, the *Dt1* lines had the shortest raceme with the fewest number of pods at the apical stems (Table 5). The soybean lines with *dt1* or either of the *dt1-t* alleles had significantly longer racemes than either the *Dt1* or *Dt2* lines. There were >10-fold more pods at the apical stem for all of the *dt1, dt1-t*, and *Dt2* lines compared to the *Dt1* lines, although there were minor significant differences in number of pods at the apical stem among the *dt1* and *Dt2* genotype categories. The soybean lines with *Dt2* alleles had intermediate raceme lengths.

**Table 5:** Mean values of agronomic traits of soybeans having different genotype combinations measured at Columbia, MO, in 2020 (20MO).

**Genotype category**	**Gene**			***n***	**Agronomic traits**					
	***Dt1***	***Dt2***	***E1***		**SD**	**DTF**	**DTM**	**Branch**	**Pod**	**RL**
Dt1_e1	*Dt1*	*dt2*	*e1-as*	4	4.9 ± 1.1 b	45.3 ± 2.2 b	122.7 ± 2.6 a	1.9 ± 2.0 a	0.8 ± 0.5 c	0.3 ± 0.4 c
dt1_e1	*dt1* (R166W)	*dt2*	*e1-as*	4	6.5 ± 1.5 a	44.7 ± 1.9 b	116.8 ± 4.4 bc	1.7 ± 1.5 ab	8.9 ± 4.0 ab	4.3 ± 2.2 a
dt1-t1_e1	*dt1-t1* (R130K)	*dt2*	*e1-as*	1	6.5 ± 1.3 a	43.7 ± 0.5 c	116.0 ± 2.5 cd	1.3 ± 1.0 ab	10.3 ± 2.6 a	4.6 ± 1.7 a
dt1-t2_e1	*dt1-t2* (R62S)	*dt2*	*e1-as*	1	6.1 ± 1.4 a	47.0 ± 0.0 a	118.0 ± 2.9 b	1.1 ± 1.4 b	8.1 ± 3.2 b	4.4 ± 2.0 a
Dt2_e1	*Dt1*	*Dt2*	*e1-as*	4	5.3 ± 1.0 b	42.1 ± 2.9 d	114.8 ± 3.0 d	1.5 ± 1.2 ab	7.8 ± 2.9 b	2.2 ± 0.7 b
			LSD (0.05)		0.7	0.7	1.4	0.8	1.4	0.7

*n represents the number of lines within each of the genotype categories. Means ± standard deviations are presented. SD represents the mean of stem diameters (mm) measured at the first, middle, and last internodes at maturity; DTF and DTM represent days to flowering and maturity, respectively; Branch represents number of branches on the main stem; Pod represents number of pods at apical stems; RL represents raceme length in cm. DTF and DTM were measured by a plot. For the other traits, a total of fifteen individual plants per line (five plants by 3 replications) were sampled for phenotyping. Mean values of each trait were obtained by averaging the means of lines within each genotype category; means followed by the same letter were not significantly different from each other at 95% confidence with Fisher's least significant difference (LSD) procedure*.

## Discussion

Soybean stem termination type is an important agronomic trait, which not only is associated with domestication and adaptation of soybeans but also affects other important agronomic characteristics, such as plant height, node number, flowering and maturity, and so forth. Three stem termination types—indeterminate, determinate, and semi-determinate—have been generally accepted, although an additional stem type, called tall determinate, was introduced about 25 years ago. Only two stem termination types, either indeterminate or determinate, have been typically utilized as genetic sources for the development of soybean cultivars targeting northern and southern production environments (Cooper, 1985). Semi-determinate, the other generally accepted stem termination with intermediate stem characteristics between indeterminate and determinate, has been recognized for its potential to improve productivity in high-yielding and lodging-prone environments but is still not widely used in various breeding programs (Cooper, 1985; Ping et al., 2014). Although there are potential agronomic benefits using the other alternative stem termination type, tall determinate, in soybean cultivar development, it has not been broadly introduced in breeding programs (Thompson et al., 1997). Here, we presented the molecular basis of the alternative genetic sources to modify plant architecture of soybean and evaluated their responses in multiple production environments to provide insight for further use in breeding programs, with the goal of genetic improvement. We found that the tall determinate stem termination type is caused by two of the previously identified missense alleles of the determinate gene *dt1* (Glyma.19g194300), *dt1-t1* (R130K), and *dt1-t2* (R62S).

Through sequence analysis at the *Dt1* locus, we found that the two tall determinate alleles were rarely present [35 lines for *dt1-t2* (R62S) and 23 lines with *dt1-t1* (R130K) were found out of 528 soybean accessions evaluated] in soybean germplasm pools (Supplementary Table 1). The semi-determinate allele (*Dt2*) was also rarely found among the analyzed soybean accessions (only 23 lines out of 528 accessions had the *Dt2* allele). In particular, none of the tall determinate or semi-determinate alleles were found among 16 North American ancestor lines evaluated (Tian et al., 2010; Zhou et al., 2015). The rare occurrences of these alleles in soybean genetic pools suggest a reason for limited use of these alleles in modern soybean breeding programs.

In this study, we presented an objective measure of the disconnect between the stem termination phenotype for soybean accessions with the actual stem termination genotype. There was a high disparity in phenotyping when soybean accessions had one of the two tall determinate alleles. The high disparity suggests that soybeans having the *dt1-t1* (R130K) or *dt1-t2* (R62S) alleles are likely to have intermediate stem characteristics, similar to those with the semi-determinate genotype allele combination. The tendency of higher rates of disparity was also observed from the analysis with a panel of 1,120 Chinese soybean cultivars (Liu et al., 2015). The higher rates of disparity between the expected phenotype resulting from each genotype in stem termination type and how observers evaluated the phenotypes suggest that the current practice of phenotypic evaluation is insufficient to classify stem termination type accurately. There are further difficulties for accurate phenotyping in stem termination type due to the effects of other confounding factors, such as environment, management, and genetic context (Li et al., 2020). Therefore, for breeding soybean lines with a specific stem architecture appropriate in an environment, it is necessary to broaden the understanding of the morphological characteristics each stem termination type has under various production environments.

Our results in the 20MO environment were consistent with the original description of early maturing soybeans with the tall determinate stem type (Thompson et al., 1997). Hartung et al. (1981) evaluated soybean stem termination types in a high plant density Nebraska field experiment and observed higher mean seed yield for a semi-determinate Clark isoline, but no or a negative effect on yield for determinate Clark isolines. We hypothesized that longer season environments would be most suitable for adjusting stem termination types since the current practice is the use of the determinate stem termination type to control lodging. Our results of targeted later maturing soybeans with *E1* alleles for field experiments in two distinct environments revealed that soybeans with tall determinate stem type have taller plant heights and a greater number of nodes while having similar stem thickness and similar pod density at the stem tip (Table 3) compared to the typical determinate type in the Southern environments. We speculate that plants with additional nodes and, therefore, the potential for more pods combined with adequate lodging resistance have the potential to result in improved seed yield, especially in high-yield production environments (Vogel et al., 2021). To incorporate more genetic diversity in soybean cultivar development, it is worthwhile to utilize the tall determinate alleles into breeding programs for soybeans with better environmental adaptability.

Our results failed to reveal obvious improvements in lodging scores. We utilized experimental soybean germplasm that contained the targeted alleles in different genetic backgrounds with the idea that the unselected genetic context would contribute only minor effects. Given the fact that the filial generation of the experimental lines having the *dt1-t1* (R130K) or *dt1-t2* (R62S) alleles was F_3:4_ (Supplementary Table 3) and the high variation in lodging scores observed from the lines, the possibility remains that additional evaluation in diverse environments will enable line selection with improved lodging scores based on stem termination alleles. Also, this study was not able to generate seed yield data depending on the genotype combinations for stem architecture due to the availability of seed, variable emergence in the studies, and difficulties in managing suitable field environments. Considering positive correlations between seed yield potential and number of pod-bearing nodes and plant height (Board, 1987; Vogel et al., 2021), the soybeans with *dt1-t1* (R130K) alleles are expected to have greater yield potential in the southern environments compared to the typical *dt1* alleles due to its effect on stem characteristics, producing a significantly higher number of nodes and strong stems. Given that the intermediate stem characteristics of the *dt1-t1* (R130K) alleles, which were also observed in semi-determinate stem types, the soybean lines with the tall determinate alleles might have potential to improve yield in particular production environments, such as high-planting density. Additional studies need to be conducted, preferably with near isogenic lines, in various production environments to reveal the impact of the tall determinate stem termination type on yield of soybean and further characterize the effects of the alleles on plant lodging.

For a prolonged period, yield has been a major concern in soybean breeding programs. There have been diverse approaches to improve the yield potential, since yield is a complex system of traits affected by diverse genetic and environmental factors. A previous study on the relationship between flowering time and yield suggested that yield improvement can be achieved by developing full-season soybeans with longer reproductive periods (Cooper, 2003). Choosing soybeans with higher plant heights, a greater number of nodes, strong stems, and longer growth periods was suggested as an ideal strategy for the genetic improvement by optimizing plant architecture (Li et al., 2020; Liu et al., 2020). Previous studies quantified the genetic changes to yield and yield stability, which occurred across eight decades, from 1928 to 2008, in soybean breeding in Northern and Southern environments in the US (Rincker et al., 2014; Boehm et al., 2019). The authors found that the yield selections over the time have resulted in shorter plants with better lodging resistance (Rincker et al., 2014; Boehm et al., 2019). Information about the stem termination types of the released soybean cultivars is missing in the articles. However, considering the absence of tall determinate alleles in US soybean ancestor lines, as well as the released soybean cultivars were targeted either to northern or southern parts of the US where the majority of soybean cultivars grown are indeterminate or determinate, respectively, it is highly probable that the tall determinate alleles have not been utilized and thus have not been examined. Therefore, it is still worthwhile to utilize the alternative stem termination type, tall determinate, into breeding programs since it has potential to improve yield by generating plants with more pod-bearing nodes along with lodging resistance.

## Data Availability Statement

The datasets presented in this study can be found in online repositories. The names of the repository/repositories and accession number(s) can be found in the article/Supplementary Material.

## Author Contributions

KB and J-HK conceived and designed the experiments with assistance from AS. J-HK, KB, VP, and ZL performed the experiments. J-HK and KB analyzed the data. J-HK and KB wrote the manuscript with inputs from AS, VP, and ZL. All authors contributed to the article and approved the submitted version.

## Conflict of Interest

The authors declare that the research was conducted in the absence of any commercial or financial relationships that could be construed as a potential conflict of interest.

## Publisher's Note

All claims expressed in this article are solely those of the authors and do not necessarily represent those of their affiliated organizations, or those of the publisher, the editors and the reviewers. Any product that may be evaluated in this article, or claim that may be made by its manufacturer, is not guaranteed or endorsed by the publisher.
