# Supplementary material for: Utilization of Plant Architecture Genes in Soybean to Positively Impact Adaptation to High Yield Environments
Source: Front Plant Sci. 2022 May 24;13:891587. doi: 10.3389/fpls.2022.891587 (PMC9171370; doi:10.3389/fpls.2022.891587)
Supplement: Supplementary file 1 [file Table_1.docx]

**Supplementary Table 1**. Summary of soybean accessions used in the study to evaluate rates of concordance between the observed stem termination type phenotypes

| **Accession** | **Genotype** |  |  |  |  | **Observed stem termination type^§^** | **Name** | **Taxonomy^§^** | **Improvement status^§^** | **Classification^§^** | **Origin^§^** | **MG^§^** | **Source of resequenced data** |
| --- | --- | --- | --- | --- | --- | --- | --- | --- | --- | --- | --- | --- | --- |
|  | ***Dt1*^†^** | ***Dt2*^†^** | ***E1*^‡^** | ***E2*^‡^** | ***E3*^‡^** |  |  |  |  |  |  |  |  |
| PI 166105 | *Dt1* | *dt2* | *E1* | *e2* | *e3* | Indeterminate | Bhart | *G. max* | Landrace | Other | Uttar Pradesh, India | VII | Valliyodan et al. (2021) |
| PI 458505 | *Dt1* | *dt2* | *E1* | *e2* | *e3* | Indeterminate | Da Bai mei | *G. max* | Landrace | Other | Liaoning Sheng, China | II | Valliyodan et al. (2021) |
| PI 632650 | *Dt1* | *dt2* | *E1* | *e2* | *e3* | Indeterminate | DT 22 | *G. max* | Landrace | Other | Vietnam | V | Valliyodan et al. (2021) |
| PI 567489 A | *Dt1* | *dt2* | *E1* | *e2* | *e3* | Indeterminate | Er da li huang dou | *G. max* | Landrace | Other | Hebei Sheng, China | IV | Valliyodan et al. (2021) |
| PI 592523 | *Dt1* | *dt2* | *E1* | *e2* | *e3* | Indeterminate | Glacier | *G. max* | Elite | Other | Minnesota, United States | 00 | Valliyodan et al. (2021) |
| PI 407965 | *Dt1* | *dt2* | *E1* | *e2* | *e3* | Indeterminate | KAERI 504-4 | *G. max* | Elite | Other | Jeollanam-do, Korea, South | V | Valliyodan et al. (2006) |
| PI 437265 D | *Dt1* | *dt2* | *E1* | *e2* | *E3* | Indeterminate | (Dobruzanca D) | *G. max* | Landrace | Other | Moldova | 0 | Valliyodan et al. (2021) |
| PI 361066 B | *Dt1* | *dt2* | *E1* | *e2* | *E3* | Indeterminate | (F. 56-17) | *G. max* | Landrace | Other | Romania | I | Valliyodan et al. (2021) |
| PI 468408 B | *Dt1* | *dt2* | *E1* | *e2* | *E3* | Indeterminate | (Qi Huang No. 1) | *G. max* | Landrace | Other | China | III | Valliyodan et al. (2021) |
| PI 602502 B | *Dt1* | *dt2* | *E1* | *e2* | *E3* | Indeterminate | (Xiong yue xiao huang dou) | *G. max* | Landrace | Other | China | IV | Valliyodan et al. (2021) |
| PI 438347 | *Dt1* | *dt2* | *E1* | *e2* | *E3* | Indeterminate | 35S.277 | *G. max* | Landrace | Other | Australia | VII | Valliyodan et al. (2021) |
| PI 153231 | *Dt1* | *dt2* | *E1* | *e2* | *E3* | Indeterminate | B-63 | *G. max* | Landrace | Other | Unknown | III | Valliyodan et al. (2021) |
| PI 567418 A | *Dt1* | *dt2* | *E1* | *e2* | *E3* | Indeterminate | Bai hei dou | *G. max* | Landrace | Other | Shanxi Sheng, China | II | Valliyodan et al. (2021) |
| PI 567428 | *Dt1* | *dt2* | *E1* | *e2* | *E3* | Indeterminate | Bai ji yao | *G. max* | Landrace | Other | Shanxi Sheng, China | IV | Valliyodan et al. (2021) |
| PI 179935 | *Dt1* | *dt2* | *E1* | *e2* | *E3* | Indeterminate | Bhart | *G. max* | Landrace | Other | Himachal Pradesh, India | VII | Valliyodan et al. (2021) |
| PI 361070 | *Dt1* | *dt2* | *E1* | *e2* | *E3* | Indeterminate | Faur | *G. max* | Landrace | Other | Romania | 0 | Valliyodan et al. (2021) |
| PI 603162 | *Dt1* | *dt2* | *E1* | *e2* | *E3* | Indeterminate | GL 2631 /96 | *G. max* | Landrace | Other | Korea, North | IV | Valliyodan et al. (2021) |
| PI 417581 | *Dt1* | *dt2* | *E1* | *e2* | *E3* | Indeterminate | H-060072 | *G. max* | Landrace | Other | United States | V | Valliyodan et al. (2021) |
| PI 603494 | *Dt1* | *dt2* | *E1* | *e2* | *E3* | Indeterminate | Hai dou zi | *G. max* | Landrace | Other | China | IV | Valliyodan et al. (2021) |
| PI 567173 | *Dt1* | *dt2* | *E1* | *e2* | *E3* | Indeterminate | Hei he 51 | *G. max* | Landrace | Other | Heilongjiang Sheng, China | 00 | Valliyodan et al. (2021) |
| PI 603526 | *Dt1* | *dt2* | *E1* | *e2* | *E3* | Indeterminate | Hei you dou | *G. max* | Landrace | Other | China | IV | Valliyodan et al. (2021) |
| PI 567439 | *Dt1* | *dt2* | *E1* | *e2* | *E3* | Indeterminate | Hong jia huang dou | *G. max* | Landrace | Other | Shanxi Sheng, China | V | Valliyodan et al. (2021) |
| PI 603555 | *Dt1* | *dt2* | *E1* | *e2* | *E3* | Indeterminate | Hua da hei dou | *G. max* | Landrace | Other | China | IV | Valliyodan et al. (2021) |
| PI 567548 | *Dt1* | *dt2* | *E1* | *e2* | *E3* | Indeterminate | Hua li hu zi | *G. max* | Landrace | Other | Shandong Sheng, China | IV | Valliyodan et al. (2021) |
| PI 603389 | *Dt1* | *dt2* | *E1* | *e2* | *E3* | Indeterminate | Huang ke | *G. max* | Landrace | Other | China | II | Valliyodan et al. (2021) |
| PI 424195 A | *Dt1* | *dt2* | *E1* | *e2* | *E3* | Indeterminate | ISZ-3 | *G. max* | Landrace | Other | Hungary | 0 | Valliyodan et al. (2021) |
| PI 587804 | *Dt1* | *dt2* | *E1* | *e2* | *E3* | Indeterminate | Jing 789 | *G. max* | Landrace | Other | Hubei Sheng, China | IV | Valliyodan et al. (2021) |
| PI 398965 | *Dt1* | *dt2* | *E1* | *e2* | *E3* | Indeterminate | KLS 628-1 | *G. max* | Landrace | Other | Jeollanam-do, Korea, South | IV | Valliyodan et al. (2021) |
| PI 437160 | *Dt1* | *dt2* | *E1* | *e2* | *E3* | Indeterminate | Krasnodarscaja 13 | *G. max* | Landrace | Other | Krasnodar, Russian Federation | I | Valliyodan et al. (2021) |
| PI 567361 | *Dt1* | *dt2* | *E1* | *e2* | *E3* | Indeterminate | Lu fang huang dou | *G. max* | Landrace | Other | Ningxia Huizi Zizhiqu, China | III | Valliyodan et al. (2021) |
| PI 567343 | *Dt1* | *dt2* | *E1* | *e2* | *E3* | Indeterminate | Ma huang dou | *G. max* | Landrace | Other | Gansu Sheng, China | V | Valliyodan et al. (2021) |
| PI 548364 | *Dt1* | *dt2* | *E1* | *e2* | *E3* | Indeterminate | Macoupin | *G. max* | Landrace | Other | Japan | IV | Valliyodan et al. (2021) |
| PI 603549 | *Dt1* | *dt2* | *E1* | *e2* | *E3* | Indeterminate | Mei dou | *G. max* | Landrace | Other | China | III | Valliyodan et al. (2021) |
| PI 123440 | *Dt1* | *dt2* | *E1* | *e2* | *E3* | Indeterminate | No. 2 | *G. max* | Landrace | Other | Myanmar | VI | Valliyodan et al. (2021) |
| PI 548479 | *Dt1* | *dt2* | *E1* | *e2* | *E3* | Indeterminate | Otootan | *G. max* | Landrace | Other | Taiwan | VIII | Valliyodan et al. (2021) |
| PI 567746 | *Dt1* | *dt2* | *E1* | *e2* | *E3* | Indeterminate | Pei xian da bai jiao | *G. max* | Landrace | Other | Jiangsu Sheng, China | IV | Valliyodan et al. (2021) |
| PI 417242 | *Dt1* | *dt2* | *E1* | *e2* | *E3* | Indeterminate | Pekin dai seitou | *G. max* | Landrace | Other | China | II | Valliyodan et al. (2021) |
| PI 495017 C | *Dt1* | *dt2* | *E1* | *e2* | *E3* | Indeterminate | (Beijing da qing don) | *G. max* | Landrace | Other | Beijing Shi, China | IV | Valliyodan et al. (2006) |
| PI 404198 B | *Dt1* | *dt2* | *E1* | *e2* | *E3* | Indeterminate | (Sun huan do) | *G. max* | Landrace | Other | China | IV | Valliyodan et al. (2006) |
| PI 567519 | *Dt1* | *dt2* | *E1* | *e2* | *E3* | Indeterminate | Bai hua chi | *G. max* | Elite | Other | Shandong Sheng, China | III | Valliyodan et al. (2006) |
| PI 548317 | *Dt1* | *dt2* | *E1* | *e2* | *E3* | Indeterminate | Columbia | *G. max* | Landrace | Other | Hebei Sheng, China | III | Valliyodan et al. (2006) |
| PI 567357 | *Dt1* | *dt2* | *E1* | *e2* | *E3* | Indeterminate | Du jia qiao huang dou | *G. max* | Landrace | Other | Ningxia Huizi Zizhiqu, China | III | Valliyodan et al. (2006) |
| PI 567719 | *Dt1* | *dt2* | *E1* | *e2* | *E3* | Indeterminate | Fu yang (43) | *G. max* | Elite | Other | Anhui Sheng, China | IV | Valliyodan et al. (2006) |
| PI 567305 | *Dt1* | *dt2* | *E1* | *e2* | *E3* | Indeterminate | Hei dou zi | *G. max* | Landrace | Other | Gansu Sheng, China | IV | Valliyodan et al. (2006) |
| PI 567387 | *Dt1* | *dt2* | *E1* | *e2* | *E3* | Indeterminate | Huang huai dou | *G. max* | Landrace | Other | Shaanxi Sheng, China | IV | Valliyodan et al. (2006) |
| PI 561271 | *Dt1* | *dt2* | *E1* | *e2* | *E3* | Indeterminate | Pei xian da quing dou | *G. max* | Elite | Other | Zhejiang Sheng, China | V | Valliyodan et al. (2006) |
| PI 84987A | *Dt1* | *dt2* | *E1* | *e2* | *E3* | Indeterminate | (Oni Hadaka) | *G. max* | Landrace | Other |  | III | Zhou et al. (2015) |
| PI 567293 | *Dt1* | *dt2* | *E1* | *e2* | *E3* | Indeterminate | Ben di huang dou | *G. max* | Landrace | Other | Gansu Sheng, China | II | Zhou et al. (2015) |
| PI 567395 | *Dt1* | *dt2* | *E1* | *e2* | *E3* | Indeterminate | Lai wa dou | *G. max* | Landrace | Other | Shaanxi Sheng, China | IV | Zhou et al. (2015) |
| PI 548593 | *Dt1* | *dt2* | *E1* | *e2* | *E3* | Indeterminate | Maple Arrow | *G. max* | Elite | NA Cultivar | Ontario, Canada | 00 | Zhou et al. (2015) |
| PI 548643 | *Dt1* | *dt2* | *E1* | *e2* | *E3* | Indeterminate | Maple Glen | *G. max* | Elite | NA Cultivar | Ontario, Canada | 00 | Zhou et al. (2015) |
| PI 548391 | *Dt1* | *dt2* | *E1* | *e2* | *E3* | Indeterminate | Mukden | *G. max* | Landrace | NA Ancestor | Liaoning Sheng, China | II | Zhou et al. (2015) |
| PI 567364 | *Dt1* | *dt2* | *E1* | *e2* | *E3* | Indeterminate | Ping luo huang da dou | *G. max* | Landrace | Other | Ningxia Huizi Zizhiqu, China | III | Zhou et al. (2015) |
| PI 438496 C | *Dt1* | *dt2* | *E1* | *E2* | *e3* | Indeterminate | (Peking) | *G. max* | Landrace | Other | United States | IV | Valliyodan et al. (2021) |
| PI 567726 | *Dt1* | *dt2* | *E1* | *E2* | *e3* | Indeterminate | Fu yang (50) | *G. max* | Landrace | Other | Anhui Sheng, China | IV | Valliyodan et al. (2021) |
| PI 438323 | *Dt1* | *dt2* | *E1* | *E2* | *e3* | Indeterminate | Grignon 53-F-3 | *G. max* | Landrace | Other | France | I | Valliyodan et al. (2021) |
| PI 209333 | *Dt1* | *dt2* | *E1* | *E2* | *e3* | Indeterminate | No. 3 | *G. max* | Landrace | Other | Hokkaidô, Japan | VI | Valliyodan et al. (2021) |
| PI 602993 | *Dt1* | *dt2* | *E1* | *E2* | *e3* | Indeterminate | Pi xian ruan tiao zhi | *G. max* | Landrace | Other | Jiangsu Sheng, China | IV | Valliyodan et al. (2021) |
| PI 157421 | *Dt1* | *dt2* | *E1* | *E2* | *e3* | Indeterminate | Ebony | *G. max* | Landrace | Other | Korea, South | III | Zhou et al. (2015) |
| PI 424038 B | *Dt1* | *dt2* | *E1* | *E2* | *E3* | Indeterminate | 74053 | *G. max* | Landrace | Other | Kyonggi, Korea, South | V | Valliyodan et al. (2021) |
| PI 567435 B | *Dt1* | *dt2* | *E1* | *E2* | *E3* | Indeterminate | (Hei hei dou) | *G. max* | Landrace | Other | Shanxi Sheng, China | III | Valliyodan et al. (2021) |
| PI 603495 B | *Dt1* | *dt2* | *E1* | *E2* | *E3* | Indeterminate | (Hong mi lan dou zi) | *G. max* | Landrace | Other | China | V | Valliyodan et al. (2021) |
| PI 438496 B | *Dt1* | *dt2* | *E1* | *E2* | *E3* | Indeterminate | (Peking) | *G. max* | Landrace | Other | United States | III | Valliyodan et al. (2021) |
| PI 567410 B | *Dt1* | *dt2* | *E1* | *E2* | *E3* | Indeterminate | (Yang huang dou) | *G. max* | Landrace | Other | Shaanxi Sheng, China | VII | Valliyodan et al. (2021) |
| PI 567415 A | *Dt1* | *dt2* | *E1* | *E2* | *E3* | Indeterminate | Bai da huang dou | *G. max* | Landrace | Other | Shanxi Sheng, China | IV | Valliyodan et al. (2021) |
| PI 567416 | *Dt1* | *dt2* | *E1* | *E2* | *E3* | Indeterminate | Bai dou | *G. max* | Landrace | Other | Shanxi Sheng, China | IV | Valliyodan et al. (2021) |
| PI 567426 | *Dt1* | *dt2* | *E1* | *E2* | *E3* | Indeterminate | Bai huang dou | *G. max* | Landrace | Other | Shanxi Sheng, China | IV | Valliyodan et al. (2021) |
| PI 391577 | *Dt1* | *dt2* | *E1* | *E2* | *E3* | Indeterminate | Cha ye sheng tou | *G. max* | Landrace | Other | Jilin Sheng, China | II | Valliyodan et al. (2021) |
| PI 548316 | *Dt1* | *dt2* | *E1* | *E2* | *E3* | Indeterminate | Cloud | *G. max* | Landrace | Other | Zhejiang Sheng, China | III | Valliyodan et al. (2021) |
| PI 548452 | *Dt1* | *dt2* | *E1* | *E2* | *E3* | Indeterminate | Dixie | *G. max* | Landrace | Other | Phyeongyang, Korea, North | V | Valliyodan et al. (2021) |
| PI 417500 | *Dt1* | *dt2* | *E1* | *E2* | *E3* | Indeterminate | Escura A | *G. max* | Landrace | Other | Brazil | VIII | Valliyodan et al. (2021) |
| PI 561371 | *Dt1* | *dt2* | *E1* | *E2* | *E3* | Indeterminate | Fen dou 15 | *G. max* | Landrace | Other | Shanxi Sheng, China | IV | Valliyodan et al. (2021) |
| PI 574477 | *Dt1* | *dt2* | *E1* | *E2* | *E3* | Indeterminate | Fen dou 31 | *G. max* | Landrace | Other | Shanxi Sheng, China | IV | Valliyodan et al. (2021) |
| PI 437662 | *Dt1* | *dt2* | *E1* | *E2* | *E3* | Indeterminate | Gun-tszu-lin 658 | *G. max* | Landrace | Other | China | II | Valliyodan et al. (2021) |
| PI 578495 | *Dt1* | *dt2* | *E1* | *E2* | *E3* | Indeterminate | Jin dou No. 4 | *G. max* | Landrace | Other | Beijing Shi, China | IV | Valliyodan et al. (2021) |
| PI 548359 | *Dt1* | *dt2* | *E1* | *E2* | *E3* | Indeterminate | Kingwa | *G. max* | Landrace | Other | Beijing Shi, China | IV | Valliyodan et al. (2021) |
| PI 507017 | *Dt1* | *dt2* | *E1* | *E2* | *E3* | Indeterminate | Madara ooha tsuru mame | *G. max* | Landrace | Other | Japan | VII | Valliyodan et al. (2021) |
| PI 209332 | *Dt1* | *dt2* | *E1* | *E2* | *E3* | Indeterminate | No. 4 | *G. max* | Landrace | Other | Hokkaidô, Japan | IV | Valliyodan et al. (2021) |
| PI 567782 | *Dt1* | *dt2* | *E1* | *E2* | *E3* | Indeterminate | OAC Dorado | *G. max* | Elite | Other | Ontario, Canada | I | Valliyodan et al. (2021) |
| PI 603492 | *Dt1* | *dt2* | *E1* | *E2* | *E3* | Indeterminate | Qi hei dou | *G. max* | Landrace | Other | China | IV | Valliyodan et al. (2021) |
| PI 324924 | *Dt1* | *dt2* | *E1* | *E2* | *E3* | Indeterminate | Rhosa | *G. max* | Landrace | Other | South Africa | V | Valliyodan et al. (2021) |
| PI 567516 C | *Dt1* | *dt2* | *E1* | *E2* | *E3* | Indeterminate | (Ba yue zha) | *G. max* | Landrace | Other | Shandong Sheng, China | IV | Valliyodan et al. (2006) |
| PI 567336 B | *Dt1* | *dt2* | *E1* | *E2* | *E3* | Indeterminate | (Lao hei dou) | *G. max* | Landrace | Other | Gansu Sheng, China | IV | Valliyodan et al. (2006) |
| PI 475783 B | *Dt1* | *dt2* | *E1* | *E2* | *E3* | Indeterminate | (Tsing 2) | *G. max* | Elite | Other | Shanxi Sheng, China | III | Valliyodan et al. (2006) |
| PI 612611 | *Dt1* | *dt2* | *E1* | *E2* | *E3* | Indeterminate | Browngilgun | *G. max* | Landrace | Other | Korea, North | III | Valliyodan et al. (2006) |
| PI 467312 | *Dt1* | *dt2* | *E1* | *E2* | *E3* | Indeterminate | Cha-mo-shi-dou | *G. max* | Landrace | Other | Jilin Sheng, China | II | Valliyodan et al. (2006) |
| PI 437655 | *Dt1* | *dt2* | *E1* | *E2* | *E3* | Indeterminate | Er-huan-jan | *G. max* | Landrace | Other | China | III | Valliyodan et al. (2006) |
| PI 548349 | *Dt1* | *dt2* | *E1* | *E2* | *E3* | Indeterminate | Ilsoy | *G. max* | Landrace | Other | Phyeongyang, Korea, North | III | Valliyodan et al. (2006) |
| PI 548298 | *Dt1* | *dt2* | *E1* | *E2* | *E3* | Indeterminate | A.K. (Harrow) | *G. max* | Landrace | NA Ancestor | China | III | Zhou et al. (2015) |
| FC 33243 | *Dt1* | *dt2* | *E1* | *E2* | *E3* | Indeterminate | Anderson | *G. max* | Elite | NA Ancestor | Unknown | IV | Zhou et al. (2015) |
| PI 437654 | *Dt1* | *dt2* | *E1* | *E2* | *E3* | Indeterminate | Er-hej-jan | *G. max* | Landrace | Other | China | III | Zhou et al. (2015) |
| PI 548348 | *Dt1* | *dt2* | *E1* | *E2* | *E3* | Indeterminate | Illini | *G. max* | Landrace | NA Ancestor | China | III | Zhou et al. (2015) |
| PI 578457 A | *Dt1* | *dt2* | *E1* |  | *E3* | Indeterminate | May den | *G. max* | Landrace | Other | Vietnam | VIII | Zhou et al. (2015) |
| PI 424078 | *Dt1* | *dt2* | *e1-as* | *e2* | *e3* | Indeterminate | 74077 | *G. max* | Landrace | Other | Gangwon-do, Korea, South | III | Valliyodan et al. (2021) |
| PI 372403 B | *Dt1* | *dt2* | *e1-as* | *e2* | *e3* | Indeterminate | (Caloria) | *G. max* | Landrace | Other | Austria | 00 | Valliyodan et al. (2021) |
| PI 578309 | *Dt1* | *dt2* | *e1-as* | *e2* | *e3* | Indeterminate | Bhatmash | *G. max* | Landrace | Other | Nepal | VI | Valliyodan et al. (2021) |
| PI 592960 | *Dt1* | *dt2* | *e1-as* | *e2* | *e3* | Indeterminate | Dong nong 38 | *G. max* | Landrace | Other | Heilongjiang Sheng, China | I | Valliyodan et al. (2021) |
| PI 514671 | *Dt1* | *dt2* | *e1-as* | *e2* | *e3* | Indeterminate | Feng shou No. 7 | *G. max* | Landrace | Other | Heilongjiang Sheng, China | 0 | Valliyodan et al. (2021) |
| PI 548325 | *Dt1* | *dt2* | *e1-as* | *e2* | *e3* | Indeterminate | Flambeau | *G. max* | Elite | Other | Russian Federation | 00 | Valliyodan et al. (2021) |
| PI 548336 | *Dt1* | *dt2* | *e1-as* | *e2* | *e3* | Indeterminate | Habaro | *G. max* | Landrace | Other | Habarovskij kraj, Russian Federation | I | Valliyodan et al. (2021) |
| PI 548571 | *Dt1* | *dt2* | *e1-as* | *e2* | *e3* | Indeterminate | Harlon | *G. max* | Landrace | Other | Ontario, Canada | I | Valliyodan et al. (2021) |
| PI 548582 | *Dt1* | *dt2* | *e1-as* | *e2* | *e3* | Indeterminate | McCall | *G. max* | Elite | Other | Minnesota, United States | 00 | Valliyodan et al. (2021) |
| PI 424298 | *Dt1* | *dt2* | *e1-as* | *e2* | *e3* | Indeterminate | KAS 300-10 | *G. max* | Landrace | Other | Chungcheongnam-do, Korea, South | IV | Valliyodan et al. (2006) |
| PI 424608 A | *Dt1* | *dt2* | *e1-as* | *e2* | *e3* | Indeterminate | KAS 681-21 | *G. max* | Landrace | Other | Gyeongsangbuk-do, Korea, South | IV | Valliyodan et al. (2006) |
| PI 508083 | *Dt1* | *dt2* | *e1-as* | *e2* | *e3* | Indeterminate | Dassel | *G. max* | Elite | NA Cultivar | Minnesota, United States | 0 | Zhou et al. (2015) |
| PI 513382 | *Dt1* | *dt2* | *e1-as* | *e2* | *e3* | Indeterminate | Glenwood | *G. max* | Elite | NA Cultivar | Minnesota, United States | 0 | Zhou et al. (2015) |
| PI 548379 | *Dt1* | *dt2* | *e1-as* | *e2* | *e3* | Indeterminate | Mandarin (Ottawa) | *G. max* | Landrace | NA Ancestor | Heilongjiang Sheng, China | 0 | Zhou et al. (2015) |
| PI 416751 | *Dt1* | *dt2* | *e1-as* | *e2* | *E3* | Indeterminate | A-B(D) | *G. max* | Landrace | Other | Japan | I | Valliyodan et al. (2021) |
| PI 417529 | *Dt1* | *dt2* | *e1-as* | *e2* | *E3* | Indeterminate | A38 | *G. max* | Landrace | Other | Germany | 0 | Valliyodan et al. (2021) |
| PI 548521 | *Dt1* | *dt2* | *e1-as* | *e2* | *E3* | Indeterminate | BSR 201 | *G. max* | Elite | Other | Iowa, United States | II | Valliyodan et al. (2021) |
| PI 297505 | *Dt1* | *dt2* | *e1-as* | *e2* | *E3* | Indeterminate | Czi ti No. 5 | *G. max* | Landrace | Other | China | I | Valliyodan et al. (2021) |
| PI 578412 | *Dt1* | *dt2* | *e1-as* | *e2* | *E3* | Indeterminate | Gong jiao 6308-1 | *G. max* | Landrace | Other | China | II | Valliyodan et al. (2021) |
| PI 567171 | *Dt1* | *dt2* | *e1-as* | *e2* | *E3* | Indeterminate | Hei he No. 1 | *G. max* | Landrace | Other | Heilongjiang Sheng, China | 00 | Valliyodan et al. (2021) |
| PI 407701 | *Dt1* | *dt2* | *e1-as* | *e2* | *E3* | Indeterminate | Hei long No. 3 | *G. max* | Landrace | Other | China | I | Valliyodan et al. (2021) |
| PI 548561 | *Dt1* | *dt2* | *e1-as* | *e2* | *E3* | Indeterminate | Hodgson | *G. max* | Elite | Other | Minnesota, United States | I | Valliyodan et al. (2021) |
| PI 398633 | *Dt1* | *dt2* | *e1-as* | *e2* | *E3* | Indeterminate | KAS 390-17-2 | *G. max* | Landrace | Other | Chungcheongbuk-do, Korea, South | V | Valliyodan et al. (2021) |
| PI 548360 | *Dt1* | *dt2* | *e1-as* | *e2* | *E3* | Indeterminate | Korean | *G. max* | Landrace | Other | Korea, North | II | Valliyodan et al. (2021) |
| PI 567558 | *Dt1* | *dt2* | *e1-as* | *e2* | *E3* | Indeterminate | Liu shi ri jin huang da dou | *G. max* | Landrace | Other | Shandong Sheng, China | III | Valliyodan et al. (2021) |
| PI 497967 | *Dt1* | *dt2* | *e1-as* | *e2* | *E3* | Indeterminate | PLSO 96 | *G. max* | Landrace | Other | Jammu and Kashmir, India | VII | Valliyodan et al. (2021) |
| PI 437169 B | *Dt1* | *dt2* | *e1-as* | *e2* | *E3* | Indeterminate | (VNIISC-4) | *G. max* | Elite | Other | Krasnodar, Russian Federation | II | Valliyodan et al. (2006) |
| PI 548511 | *Dt1* | *dt2* | *e1-as* | *e2* | *E3* | Indeterminate | Beeson 80 | *G. max* | Elite | NA Cultivar | Indiana, United States | II | Valliyodan et al. (2006) |
| PI 518751 | *Dt1* | *dt2* | *e1-as* | *e2* | *E3* | Indeterminate | NS-20 | *G. max* | Elite | Other | Former Serbia and Montenegro | II | Valliyodan et al. (2006) |
| PI 533655 | *Dt1* | *dt2* | *e1-as* | *e2* | *E3* | Indeterminate | Burlison | *G. max* | Elite | NA Cultivar | Illinois, United States | II | Zhou et al. (2015) |
| PI 548512 | *Dt1* | *dt2* | *e1-as* | *e2* | *E3* | Indeterminate | Century | *G. max* | Elite | NA Cultivar | Indiana, United States | II | Zhou et al. (2015) |
| PI 542403 | *Dt1* | *dt2* | *e1-as* | *e2* | *E3* | Indeterminate | Dawson | *G. max* | Elite | NA Cultivar | Minnesota, United States | 0 | Zhou et al. (2015) |
| PI 548573 | *Dt1* | *dt2* | *e1-as* | *e2* | *E3* | Indeterminate | Harosoy | *G. max* | Elite | NA Cultivar | Ontario, Canada | II | Zhou et al. (2015) |
| PI 547680 | *Dt1* | *dt2* | *e1-as* | *e2* | *E3* | Indeterminate | L62-17 | *G. max* | Elite | NA Cultivar | Illinois, United States | II | Zhou et al. (2015) |
| PI 547686 | *Dt1* | *dt2* | *e1-as* | *e2* | *E3* | Indeterminate | L62-956 | *G. max* | Elite | NA Cultivar | Illinois, United States | II | Zhou et al. (2015) |
| PI 547690 | *Dt1* | *dt2* | *e1-as* | *e2* | *E3* | Indeterminate | L63-1212 | *G. max* | Elite | NA Cultivar | Illinois, United States | II | Zhou et al. (2015) |
| PI 253658 B | *Dt1* | *dt2* | *e1-as* | *e2* | *E3* | Indeterminate | No. 9 | *G. max* | Landrace | Other | China | I | Zhou et al. (2015) |
| PI 518750 | *Dt1* | *dt2* | *e1-as* | *e2* | *E3* | Indeterminate | NS-16 | *G. max* | Elite | Other | Former Serbia and Montenegro | I | Zhou et al. (2015) |
| PI 548638 | *Dt1* | *dt2* | *e1-as* | *e2* | *E3* | Indeterminate | OAC Libra | *G. max* | Elite | NA Cultivar | Ontario, Canada | 0 | Zhou et al. (2015) |
| PI 548644 | *Dt1* | *dt2* | *e1-as* | *e2* | *E3* | Indeterminate | OAC Musca | *G. max* | Elite | NA Cultivar | Ontario, Canada | 0 | Zhou et al. (2015) |
| PI 591435 | *Dt1* | *dt2* | *e1-as* | *e2* | *E3* | Indeterminate | OT94-41 | *G. max* | Elite | NA Cultivar | Ontario, Canada | I | Zhou et al. (2015) |
| PI 561389 B | *Dt1* | *dt2* | *e1-as* | *E2* | *e3* | Indeterminate | (Okura Natto) | *G. max* | Landrace | Other | Japan | 0 | Valliyodan et al. (2021) |
| PI 361080 | *Dt1* | *dt2* | *e1-as* | *E2* | *e3* | Indeterminate | Kormovaia 15 | *G. max* | Landrace | Other | Russian Federation | II | Valliyodan et al. (2021) |
| PI 266806 C | *Dt1* | *dt2* | *e1-as* | *E2* | *e3* | Indeterminate | No. 4 | *G. max* | Landrace | Other | Hebei Sheng, China | II | Valliyodan et al. (2021) |
| PI 548520 | *Dt1* | *dt2* | *e1-as* | *E2* | *e3* | Indeterminate | Preston | *G. max* | Elite | Other | Iowa, United States | II | Valliyodan et al. (2021) |
| PI 548311 | *Dt1* | *dt2* | *e1-as* | *E2* | *e3* | Indeterminate | Capital | *G. max* | Elite | NA Ancestor | Ontario, Canada | 0 | Zhou et al. (2015) |
| PI 548540 | *Dt1* | *dt2* | *e1-as* | *E2* | *e3* | Indeterminate | Corsoy | *G. max* | Elite | NA Cultivar | Iowa, United States | II | Zhou et al. (2015) |
| PI 578375 B | *Dt1* | *dt2* | *e1-as* | *E2* | *E3* | Indeterminate | (Aan tu dang di hei dou) | *G. max* | Landrace | Other | China | I | Valliyodan et al. (2021) |
| PI 639550 E | *Dt1* | *dt2* | *e1-as* | *E2* | *E3* | Indeterminate | (KSHI 713) | *G. max* | Landrace | Other | Moldova | II | Valliyodan et al. (2021) |
| PI 605765 B | *Dt1* | *dt2* | *e1-as* | *E2* | *E3* | Indeterminate | (Ninh minh) | *G. max* | Landrace | Other | Tuyên Quang, Vietnam | II | Valliyodan et al. (2021) |
| PI 556511 | *Dt1* | *dt2* | *e1-as* | *E2* | *E3* | Indeterminate | A3127 | *G. max* | Elite | Other | United States | III | Valliyodan et al. (2021) |
| PI 548313 | *Dt1* | *dt2* | *e1-as* | *E2* | *E3* | Indeterminate | Chestnut | *G. max* | Landrace | Other | Habarovskij kraj, Russian Federation | III | Valliyodan et al. (2021) |
| PI 378663 | *Dt1* | *dt2* | *e1-as* | *E2* | *E3* | Indeterminate | Habarovskaja II | *G. max* | Landrace | Other | Russian Federation | I | Valliyodan et al. (2021) |
| PI 561318 A | *Dt1* | *dt2* | *e1-as* | *E2* | *E3* | Indeterminate | Hui nan bai hua xiao hei dou | *G. max* | Landrace | Other | Beijing Shi, China | I | Valliyodan et al. (2021) |
| PI 603442 | *Dt1* | *dt2* | *e1-as* | *E2* | *E3* | Indeterminate | Ke qi xiao hei dou | *G. max* | Landrace | Other | China | III | Valliyodan et al. (2021) |
| PI 548383 | *Dt1* | *dt2* | *e1-as* | *E2* | *E3* | Indeterminate | Mansoy | *G. max* | Landrace | Other | Heilongjiang Sheng, China | III | Valliyodan et al. (2021) |
| PI 253661 B | *Dt1* | *dt2* | *e1-as* | *E2* | *E3* | Indeterminate | No. 12 | *G. max* | Landrace | Other | China | III | Valliyodan et al. (2021) |
| PI 548400 | *Dt1* | *dt2* | *e1-as* | *E2* | *E3* | Indeterminate | Patoka | *G. max* | Landrace | Other | Heilongjiang Sheng, China | IV | Valliyodan et al. (2021) |
| PI 552538 | *Dt1* | *dt2* | *e1-as* | *E2* | *E3* | Indeterminate | Dunbar | *G. max* | Elite | NA Cultivar | Nebraska, United States | III | Valliyodan et al. (2006) |
| PI 542044 | *Dt1* | *dt2* | *e1-as* | *E2* | *E3* | Indeterminate | Kunitz | *G. max* | Elite | NA Cultivar | Illinois, United States | III | Valliyodan et al. (2006) |
| PI 639740 | *Dt1* | *dt2* | *e1-as* | *E2* | *E3* | Indeterminate | LD00-3309 | *G. max* | Elite | NA Cultivar | Illinois, United States | IV | Valliyodan et al. (2006) |
| PI 593258 | *Dt1* | *dt2* | *e1-as* | *E2* | *E3* | Indeterminate | Macon | *G. max* | Elite | Other | Illinois, United States | III | Valliyodan et al. (2006) |
| PI 597387 | *Dt1* | *dt2* | *e1-as* | *E2* | *E3* | Indeterminate | Pana | *G. max* | Elite | NA Cultivar | Illinois, United States | III | Valliyodan et al. (2006) |
| PI 547460 | *Dt1* | *dt2* | *e1-as* | *E2* | *E3* | Indeterminate | L64-1083 | *G. max* | Elite | NA Cultivar | Illinois, United States | IV | Zhou et al. (2015) |
| PI 547562 | *Dt1* | *dt2* | *e1-as* | *E2* | *E3* | Indeterminate | L72-2157 | *G. max* | Elite | NA Cultivar | Illinois, United States | IV | Zhou et al. (2015) |
| PI 547862 | *Dt1* | *dt2* | *e1-as* | *E2* | *E3* | Indeterminate | L83-570 | *G. max* | Elite | NA Cultivar | Illinois, United States | III | Valliyodan et al. (2006) |
| PI 591511 | *Dt1* | *dt2* | *e1-as* | *E2* | *E3* | Indeterminate | L89-1581 | *G. max* | Elite | NA Cultivar | Illinois, United States | III | Zhou et al. (2015) |
| PI 591539 | *Dt1* | *dt2* | *e1-as* | *E2* | *E3* | Indeterminate | L91-8558 | *G. max* | Elite | NA Cultivar | Illinois, United States | III | Valliyodan et al. (2006) |
| PI 591495 | *Dt1* | *dt2* | *e1-as* | *E2* | *E3* | Indeterminate | L93-2740 | *G. max* | Elite | NA Cultivar | Illinois, United States | IV | Zhou et al. (2015) |
| PI 548362 | *Dt1* | *dt2* | *e1-as* | *E2* | *E3* | Indeterminate | Lincoln | *G. max* | Elite | NA Ancestor | Illinois, United States | III | Zhou et al. (2015) |
| PI 515961 | *Dt1* | *dt2* | *e1-as* | *E2* | *E3* | Indeterminate | Pennyrile | *G. max* | Elite | NA Cultivar | Kentucky, United States | IV | Zhou et al. (2015) |
| PI 548603 | *Dt1* | *dt2* | *e1-as* | *E2* | *E3* | Indeterminate | Perry | *G. max* | Elite | NA Ancestor | Indiana, United States | IV | Zhou et al. (2015) |
| PI 639559 B | *Dt1* | *dt2* | *e1-as* |  | *E3* | Indeterminate | (VYTKA 2) | *G. max* | Landrace | Other | Ukraine | II | Valliyodan et al. (2021) |
| PI 547716 | *Dt1* | *dt2* | *e1-as* |  |  | Indeterminate | L62-667 | *G. max* | Elite | NA Cultivar | Illinois, United States | II | Zhou et al. (2015) |
| PI 378658 | *Dt1* | *dt2* |  | *e2* | *E3* | Indeterminate | Dnepropetrovsk 12 | *G. max* | Landrace | Other | Dnipropetrovsk, Ukraine | 0 | Valliyodan et al. (2021) |
| PI 548572 | *Dt1* | *dt2* |  | *e2* | *E3* | Indeterminate | Harly | *G. max* | Elite | Other | Ontario, Canada | I | Valliyodan et al. (2021) |
| PI 504288 | *Dt1* | *dt2* |  |  |  | Indeterminate | S | *G. max* | Landrace | Other | Iwate, Japan | V | Valliyodan et al. (2021) |
| PI 438335 | *Dt1* | *dt2* |  |  |  | Indeterminate | SAO 196-C | *G. max* | Landrace | Other | Algeria | III | Valliyodan et al. (2021) |
| PI 548411 | *Dt1* | *dt2* |  |  |  | Indeterminate | Seneca | *G. max* | Landrace | Other | China | II | Valliyodan et al. (2021) |
| PI 479735 | *Dt1* | *dt2* |  |  |  | Indeterminate | Silihuang | *G. max* | Landrace | Other | Jilin Sheng, China | III | Valliyodan et al. (2021) |
| PI 548619 | *Dt1* | *dt2* |  |  |  | Indeterminate | Sparks | *G. max* | Elite | Other | Kansas, United States | IV | Valliyodan et al. (2021) |
| PI 180501 | *Dt1* | *dt2* |  |  |  | Indeterminate | Strain No. 18 | *G. max* | Landrace | Other | Germany | 0 | Valliyodan et al. (2021) |
| PI 593953 | *Dt1* | *dt2* |  |  |  | Indeterminate | Sui nong No. 10 | *G. max* | Landrace | Other | China | I | Valliyodan et al. (2021) |
| PI 548193 | *Dt1* | *dt2* |  |  |  | Indeterminate | T201 | *G. max* | Landrace | Other | Iowa, United States | IV | Valliyodan et al. (2021) |
| PI 548200 | *Dt1* | *dt2* |  |  |  | Indeterminate | T211H | *G. max* | Landrace | Other | Illinois, United States | IV | Valliyodan et al. (2021) |
| PI 587588 A | *Dt1* | *dt2* |  |  |  | Indeterminate | Tai xing niu mao huang yi | *G. max* | Landrace | Other | Jiangsu Sheng, China | IV | Valliyodan et al. (2021) |
| PI 548490 | *Dt1* | *dt2* |  |  |  | Indeterminate | Tanner | *G. max* | Landrace | Other | Taiwan | VII | Valliyodan et al. (2021) |
| PI 632418 | *Dt1* | *dt2* |  |  |  | Indeterminate | Tara | *G. max* | Elite | Other | Maryland, United States | V | Valliyodan et al. (2021) |
| PI 417381 | *Dt1* | *dt2* |  |  |  | Indeterminate | Tenpoku shirome | *G. max* | Landrace | Other | Hokkaidô, Japan | 0 | Valliyodan et al. (2021) |
| PI 578503 | *Dt1* | *dt2* |  |  |  | Indeterminate | Tie jia si li huang | *G. max* | Landrace | Other | China | I | Valliyodan et al. (2021) |
| PI 518668 | *Dt1* | *dt2* |  |  |  | Indeterminate | TN 4-86 | *G. max* | Elite | Other | Tennessee, United States | IV | Valliyodan et al. (2021) |
| PI 437165 A | *Dt1* | *dt2* |  |  |  | Indeterminate | Toncostebelnaja 27 | *G. max* | Landrace | Other | Krasnodar, Russian Federation | I | Valliyodan et al. (2021) |
| PI 507467 | *Dt1* | *dt2* |  |  |  | Indeterminate | Tousan kei F 764 | *G. max* | Landrace | Other | Japan | IV | Valliyodan et al. (2021) |
| PI 507471 | *Dt1* | *dt2* |  |  |  | Indeterminate | Tousan kei na 16 | *G. max* | Landrace | Other | Japan | III | Valliyodan et al. (2021) |
| PI 594307 | *Dt1* | *dt2* |  |  |  | Indeterminate | Tsurusengoku | *G. max* | Landrace | Other | Japan | VIII | Valliyodan et al. (2021) |
| PI 437376 A | *Dt1* | *dt2* |  |  |  | Indeterminate | Ussurijscaja 308 | *G. max* | Landrace | Other | Primorye, Russian Federation | I | Valliyodan et al. (2021) |
| PI 437991 B | *Dt1* | *dt2* |  |  |  | Indeterminate | VIR 1657 | *G. max* | Landrace | Other | China | 0 | Valliyodan et al. (2021) |
| PI 438019 B | *Dt1* | *dt2* |  |  |  | Indeterminate | VIR 1883 | *G. max* | Landrace | Other | China | II | Valliyodan et al. (2021) |
| PI 639528 B | *Dt1* | *dt2* |  |  |  | Indeterminate | VIR 233 | *G. max* | Landrace | Other | Primorye, Russian Federation | II | Valliyodan et al. (2021) |
| PI 437110 A | *Dt1* | *dt2* |  |  |  | Indeterminate | VIR 244 | *G. max* | Landrace | Other | Russian Federation | III | Valliyodan et al. (2021) |
| PI 437112 A | *Dt1* | *dt2* |  |  |  | Indeterminate | VIR 249 | *G. max* | Landrace | Other | Russian Federation | II | Valliyodan et al. (2021) |
| PI 438083 | *Dt1* | *dt2* |  |  |  | Indeterminate | VIR 2506 | *G. max* | Landrace | Other | China | II | Valliyodan et al. (2021) |
| PI 437788 A | *Dt1* | *dt2* |  |  |  | Indeterminate | VIR 3018 | *G. max* | Landrace | Other | China | II | Valliyodan et al. (2021) |
| PI 639543 | *Dt1* | *dt2* |  |  |  | Indeterminate | VIR 3715 | *G. max* | Landrace | Other | Primorye, Russian Federation | II | Valliyodan et al. (2021) |
| PI 437500 A | *Dt1* | *dt2* |  |  |  | Indeterminate | VIR 3810 | *G. max* | Landrace | Other | Primorye, Russian Federation | I | Valliyodan et al. (2021) |
| PI 437505 | *Dt1* | *dt2* |  |  |  | Indeterminate | VIR 3853 | *G. max* | Landrace | Other | Primorye, Russian Federation | II | Valliyodan et al. (2021) |
| PI 438230 A | *Dt1* | *dt2* |  |  |  | Indeterminate | VIR 4521 | *G. max* | Landrace | Other | China | I | Valliyodan et al. (2021) |
| PI 438239 B | *Dt1* | *dt2* |  |  |  | Indeterminate | VIR 4536 | *G. max* | Landrace | Other | China | I | Valliyodan et al. (2021) |
| PI 639570 | *Dt1* | *dt2* |  |  |  | Indeterminate | VIR 7010 | *G. max* | Landrace | Other | Philippines | V | Valliyodan et al. (2021) |
| PI 438500 | *Dt1* | *dt2* |  |  |  | Indeterminate | Virginia | *G. max* | Landrace | Other | United States | III | Valliyodan et al. (2021) |
| PI 567238 | *Dt1* | *dt2* |  |  |  | Indeterminate | W6 6210 | *G. max* | Landrace | Other | Yunnan Sheng, China | IX | Valliyodan et al. (2021) |
| PI 548524 | *Dt1* | *dt2* |  |  |  | Indeterminate | Weber | *G. max* | Elite | Other | Iowa, United States | I | Valliyodan et al. (2021) |
| PI 548427 | *Dt1* | *dt2* |  |  |  | Indeterminate | Wilson | *G. max* | Landrace | Other | Liaoning Sheng, China | IV | Valliyodan et al. (2021) |
| PI 445824 A | *Dt1* | *dt2* |  |  |  | Indeterminate | Wolfsthaler | *G. max* | Landrace | Other | Germany | 000 | Valliyodan et al. (2021) |
| PI 548633 | *Dt1* | *dt2* |  |  |  | Indeterminate | Wye | *G. max* | Elite | Other | Maryland, United States | IV | Valliyodan et al. (2021) |
| PI 603399 | *Dt1* | *dt2* |  |  |  | Indeterminate | Xiao bai qi | *G. max* | Landrace | Other | China | II | Valliyodan et al. (2021) |
| PI 567407 | *Dt1* | *dt2* |  |  |  | Indeterminate | Xiao dou | *G. max* | Landrace | Other | Shaanxi Sheng, China | V | Valliyodan et al. (2021) |
| PI 567408 | *Dt1* | *dt2* |  |  |  | Indeterminate | Xiao jin huang | *G. max* | Landrace | Other | Shaanxi Sheng, China | V | Valliyodan et al. (2021) |
| PI 495020 | *Dt1* | *dt2* |  |  |  | Indeterminate | Xu dou 2 | *G. max* | Landrace | Other | Beijing Shi, China | IV | Valliyodan et al. (2021) |
| PI 603290 | *Dt1* | *dt2* |  |  |  | Indeterminate | Zao shu 18 | *G. max* | Landrace | Other | China | I | Valliyodan et al. (2021) |
| PI 592937 | *Dt1* | *dt2* |  |  |  | Indeterminate | ZDD 18846 | *G. max* | Landrace | Other | China | IV | Valliyodan et al. (2021) |
| PI 592940 | *Dt1* | *dt2* |  |  |  | Indeterminate | ZDD 18849 | *G. max* | Landrace | Other | China | IV | Valliyodan et al. (2021) |
| PI 603556 | *Dt1* | *dt2* |  |  |  | Indeterminate | ZDD08563 | *G. max* | Landrace | Other | China | III | Valliyodan et al. (2021) |
| PI 603559 | *Dt1* | *dt2* |  |  |  | Indeterminate | ZDD08590 | *G. max* | Landrace | Other | China | IV | Valliyodan et al. (2021) |
| PI 467347 | *Dt1* | *dt2* |  |  |  | Indeterminate | Zi-hua-cuo-zi | *G. max* | Landrace | Other | Jilin Sheng, China | II | Valliyodan et al. (2021) |
| PI 612754 | *Dt1* | *dt2* |  |  |  | Indeterminate | ZY 645 | *G. max* | Landrace | Other | China | I | Valliyodan et al. (2021) |
| PI 549017 | *Dt1* | *dt2* |  |  |  | Indeterminate | ZYD 3938 | *G. max* | Landrace | Other | Ningxia Huizi Zizhiqu, China | IV | Valliyodan et al. (2021) |
| PI 549018 | *Dt1* | *dt2* |  |  |  | Indeterminate | ZYD 3939 | *G. max* | Landrace | Other | Ningxia Huizi Zizhiqu, China | V | Valliyodan et al. (2021) |
| FC 029333 | *Dt1* | *dt2* |  |  |  | Indeterminate |  | *G. max* | Landrace | Other |  | III | Valliyodan et al. (2021) |
| FC 031697 | *Dt1* | *dt2* |  |  |  | Indeterminate |  | *G. max* | Landrace | Other |  | IV | Valliyodan et al. (2021) |
| PI 054591 | *Dt1* | *dt2* |  |  |  | Indeterminate |  | *G. max* | Landrace | Other |  | III | Valliyodan et al. (2021) |
| PI 054614 | *Dt1* | *dt2* |  |  |  | Indeterminate |  | *G. max* | Landrace | Other |  | IV | Valliyodan et al. (2021) |
| PI 058955 | *Dt1* | *dt2* |  |  |  | Indeterminate |  | *G. max* | Landrace | Other |  | IV | Valliyodan et al. (2021) |
| PI 062203 | *Dt1* | *dt2* |  |  |  | Indeterminate |  | *G. max* | Landrace | Other |  | V | Valliyodan et al. (2021) |
| PI 070080 | *Dt1* | *dt2* |  |  |  | Indeterminate |  | *G. max* | Landrace | Other |  | III | Valliyodan et al. (2021) |
| PI 071465 | *Dt1* | *dt2* |  |  |  | Indeterminate |  | *G. max* | Landrace | Other |  | V | Valliyodan et al. (2021) |
| PI 081041 | *Dt1* | *dt2* |  |  |  | Indeterminate |  | *G. max* | Landrace | Other |  | III | Valliyodan et al. (2021) |
| PI 081785 | *Dt1* | *dt2* |  |  |  | Indeterminate |  | *G. max* | Landrace | Other | Hokkaido, Japan | III | Valliyodan et al. (2021) |
| PI 083881 | *Dt1* | *dt2* |  |  |  | Indeterminate |  | *G. max* | Landrace | Other |  | IV | Valliyodan et al. (2021) |
| PI 084637 | *Dt1* | *dt2* |  |  |  | Indeterminate |  | *G. max* | Landrace | Other |  | II | Valliyodan et al. (2021) |
| PI 084656 | *Dt1* | *dt2* |  |  |  | Indeterminate |  | *G. max* | Landrace | Other |  | III | Valliyodan et al. (2021) |
| PI 084973 | *Dt1* | *dt2* |  |  |  | Indeterminate |  | *G. max* | Landrace | Other |  | III | Valliyodan et al. (2021) |
| PI 086904 | *Dt1* | *dt2* |  |  |  | Indeterminate |  | *G. max* | Landrace | Other |  | VI | Valliyodan et al. (2021) |
| PI 087620 | *Dt1* | *dt2* |  |  |  | Indeterminate |  | *G. max* | Landrace | Other |  | III | Valliyodan et al. (2021) |
| PI 088788 | *Dt1* | *dt2* |  |  |  | Indeterminate |  | *G. max* | Landrace | Other |  | III | Valliyodan et al. (2021) |
| PI 089775 | *Dt1* | *dt2* |  |  |  | Indeterminate |  | *G. max* | Landrace | Other |  | VI | Valliyodan et al. (2021) |
| PI 090763 | *Dt1* | *dt2* |  |  |  | Indeterminate |  | *G. max* | Landrace | Other |  | IV | Valliyodan et al. (2021) |
| PI 091160 | *Dt1* | *dt2* |  |  |  | Indeterminate |  | *G. max* | Landrace | Other |  | III | Valliyodan et al. (2021) |
| PI 092651 | *Dt1* | *dt2* |  |  |  | Indeterminate |  | *G. max* | Landrace | Other |  | IV | Valliyodan et al. (2021) |
| PI 291294 | *Dt1* | *dt2* |  |  |  | Indeterminate |  | *G. max* | Landrace | Other | Heilongjiang Sheng, China | I | Valliyodan et al. (2021) |
| PI 468908 | *Dt1* | *dt2* |  |  |  | Indeterminate |  | *G. max* | Landrace | Other | Jilin Sheng, China | 000 | Valliyodan et al. (2021) |
| PI 475820 | *Dt1* | *dt2* |  |  |  | Indeterminate |  | *G. max* | Landrace | Other | Xinjiang Uygur Zizhiqu, China | II | Valliyodan et al. (2021) |
| PI 291309 D | *Dt1* | *dt2* |  |  |  | Indeterminate |  | *G. max* | Landrace | Other | Heilongjiang Sheng, China | II | Valliyodan et al. (2021) |
| PI 291310 C | *Dt1* | *dt2* |  |  |  | Indeterminate |  | *G. max* | Landrace | Other | Heilongjiang Sheng, China | II | Valliyodan et al. (2021) |
| PI 342619 A | *Dt1* | *dt2* |  |  |  | Indeterminate |  | *G. max* | Landrace | Other | Primorye, Russian Federation | 0 | Valliyodan et al. (2021) |
| PI 054615 -1 | *Dt1* | *dt2* |  |  |  | Indeterminate |  | *G. max* | Landrace | Other |  | III | Valliyodan et al. (2021) |
| PI 068732 -1 | *Dt1* | *dt2* |  |  |  | Indeterminate |  | *G. max* | Landrace | Other |  | III | Valliyodan et al. (2021) |
| PI 091159 -4 | *Dt1* | *dt2* |  |  |  | Indeterminate |  | *G. max* | Landrace | Other |  | IV | Valliyodan et al. (2021) |
| PI 548415 | *Dt1* | *dt2* |  |  |  | Indeterminate | Sooty | *G. max* | Landrace | Other | Zhejiang Sheng, China | IV | Valliyodan et al. (2006) |
| PI 438258 | *Dt1* | *dt2* |  |  |  | Indeterminate | VIR 4714 | *G. max* | Elite | Other | China | II | Valliyodan et al. (2006) |
| PI 567230 | *Dt1* | *dt2* |  |  |  | Indeterminate | WJK-PRC-23 | *G. max* | Landrace | Other | Shaanxi Sheng, China | V | Valliyodan et al. (2006) |
| PI 567354 | *Dt1* | *dt2* |  |  |  | Indeterminate | You huang dou | *G. max* | Landrace | Other | Gansu Sheng, China | IV | Valliyodan et al. (2006) |
| FC 031721 | *Dt1* | *dt2* |  |  |  | Indeterminate |  | *G. max* | Landrace | Other |  | VI | Valliyodan et al. (2006) |
| PI 086006 | *Dt1* | *dt2* |  |  |  | Indeterminate |  | *G. max* | Landrace | Other |  | III | Valliyodan et al. (2006) |
| PI 087617 | *Dt1* | *dt2* |  |  |  | Indeterminate |  | *G. max* | Elite | Other |  | III | Valliyodan et al. (2006) |
| PI 407729 | *Dt1* | *dt2* |  |  |  | Indeterminate |  | *G. max* | Landrace | Other | Beijing Shi, China | IV | Valliyodan et al. (2006) |
| PI 468915 | *Dt1* | *dt2* |  |  |  | Indeterminate |  | *G. max* | Landrace | Other | Liaoning Sheng, China | II | Valliyodan et al. (2006) |
| PI 549031 | *Dt1* | *dt2* |  |  |  | Indeterminate |  | *G. max* | Landrace | Other | Beijing Shi, China | III | Valliyodan et al. (2006) |
| PI 603176 A | *Dt1* | *dt2* |  |  |  | Indeterminate |  | *G. max* | Elite | Other | Korea, North | IV | Valliyodan et al. (2006) |
| PI 087631 -1 | *Dt1* | *dt2* |  |  |  | Indeterminate |  | *G. max* | Landrace | Other |  | III | Valliyodan et al. (2006) |
| PI 548488 | *Dt1* | *dt2* |  |  |  | Indeterminate | S-100 | *G. max* | Landrace | NA Ancestor | Heilongjiang Sheng, China | V | Zhou et al. (2015) |
| PI 548631 | *Dt1* | *dt2* |  |  |  | Indeterminate | Williams | *G. max* | Elite | NA Cultivar | Illinois, United States | III | Zhou et al. (2015) |
| PI 603318 | *Dt1* | *dt2* |  |  |  | Indeterminate | Xiao zhu yao | *G. max* | Landrace | Other | China | I | Zhou et al. (2015) |
| PI 548634 | *Dt1* | *dt2* |  |  |  | Indeterminate | Zane | *G. max* | Elite | NA Cultivar | Ohio, United States | III | Zhou et al. (2015) |
| PI 603424 A | *Dt1* | *dt2* |  |  |  | Indeterminate | ZDD007871 | *G. max* | Landrace | Other | China | 0 | Zhou et al. (2015) |
| PI 603420 | *Dt1* | *dt2* |  |  |  | Indeterminate | ZDD01501 | *G. max* | Landrace | Other | China | II | Zhou et al. (2015) |
| PI 89138 | *Dt1* | *dt2* |  |  |  | Indeterminate | Zontanorukon | *G. max* | Landrace | Other | Hamkyeongpukto, Korea, North | II | Zhou et al. (2015) |
| PI 339734 | *Dt1* | *dt2* |  |  |  | Indeterminate |  | *G. max* | Landrace | Other | Gangwon-do, Korea, South | IV | Zhou et al. (2015) |
| PI 603675 | *Dt1* | *dt2* | *E1* | *e2* | *E3* | Semi-determinate | Huai yin gua dou jia | *G. max* | Landrace | Other | China | III | Valliyodan et al. (2021) |
| PI 594599 | *Dt1* | *dt2* | *E1* | *e2* | *E3* | Semi-determinate | Chang de chun hei dou | *G. max* | Elite | Other | Hunan Sheng, China | IV | Valliyodan et al. (2006) |
| PI 437321 | *Dt1* | *dt2* | *E1* | *e2* | *E3* | Semi-determinate | Dunganscaja 462 | *G. max* | Landrace | Other | Primorye, Russian Federation | III | Zhou et al. (2015) |
| PI 88479 | *Dt1* | *dt2* | *E1* | *e2* | *E3* | Semi-determinate | Kungchuling Improved No. 77 | *G. max* | Landrace | Other | Jilin Sheng, China | II | Zhou et al. (2015) |
| PI 594615 | *Dt1* | *dt2* | *E1* | *e2* | *E3* | Semi-determinate | Liu yue zao | *G. max* | Landrace | Other | Guizhou Sheng, China | IV | Zhou et al. (2015) |
| PI 407708 A | *Dt1* | *dt2* | *e1-as* | *e2* | *e3* | Semi-determinate | Feng shou No. 10 | *G. max* | Landrace | Other | Heilongjiang Sheng, China | 0 | Valliyodan et al. (2021) |
| PI 548406 | *Dt1* | *dt2* | *e1-as* | *e2* | *e3* | Semi-determinate | Richland | *G. max* | Landrace | NA Ancestor | Jilin Sheng, China | II | Zhou et al. (2015) |
| PI 297520 | *Dt1* | *dt2* | *e1-as* | *e2* | *E3* | Semi-determinate | Iregi Universal | *G. max* | Landrace | Other | Hungary | 0 | Valliyodan et al. (2021) |
| PI 603426 G | *Dt1* | *dt2* | *e1-as* | *E2* | *E3* | Semi-determinate | (Ben di yuan huang dou) | *G. max* | Landrace | Other | China | II | Valliyodan et al. (2021) |
| PI 437776 | *Dt1* | *dt2* |  |  |  | Semi-determinate | VIR 1302 | *G. max* | Landrace | Other | China | III | Valliyodan et al. (2021) |
| PI 603345 | *Dt1* | *dt2* |  |  |  | Semi-determinate | ZDD00403 | *G. max* | Landrace | Other | China | II | Valliyodan et al. (2021) |
| PI 094159 -3 | *Dt1* | *dt2* |  |  |  | Semi-determinate |  | *G. max* | Landrace | Other |  | IV | Valliyodan et al. (2021) |
| PI 458515 | *Dt1* | *dt2* |  |  |  | Semi-determinate | Tie Zhugan | *G. max* | Landrace | Other | Shandong Sheng, China | IV | Valliyodan et al. (2006) |
| PI 80822 | *Dt1* | *dt2* |  |  |  | Semi-determinate | Shiheigai Shirobana | *G. max* | Landrace | Other | China | III | Zhou et al. (2015) |
| PI 548182 | *Dt1* | *dt2* |  |  |  | Semi-determinate | T157 | *G. max* | Elite | NA Cultivar | Illinois, United States | III | Zhou et al. (2015) |
| PI 437944 | *Dt1* | *dt2* |  |  |  | Semi-determinate | VIR 569 | *G. max* | Landrace | Other | China | II | Zhou et al. (2015) |
| PI 587848 | *Dt1* | *dt2* |  |  |  | Semi-determinate | Wu chang hei dong dou | *G. max* | Landrace | Other | Hubei Sheng, China | V | Zhou et al. (2015) |
| PI 578499 A | *Dt1* | *dt2* | *E1* | *E2* | *E3* | Determinate | Lu yue bai | *G. max* | Landrace | Other | China | II | Valliyodan et al. (2021) |
| PI 587552 | *Dt1* | *dt2* | *E1* | *E2* | *E3* | Determinate | Nan jing da ping ding huang yi No. 1 | *G. max* | Landrace | Other | Jiangsu Sheng, China | VII | Zhou et al. (2015) |
| PI 506942 | *Dt1* | *dt2* | *e1-as* | *e2* | *E3* | Determinate | Koushurei 235 | *G. max* | Landrace | Other | Japan | II | Valliyodan et al. (2021) |
| PI 248515 | *Dt1* | *dt2* |  |  |  | Determinate | White Hilum Iwata Variety No. 2 | *G. max* | Elite | Other | Japan | IV | Valliyodan et al. (2006) |
| PI 567488 A | *Dt1* | *Dt2* | *E1* | *e2* | *E3* | Indeterminate | Di liu huang dou No. 2 | *G. max* | Landrace | Other | Hebei Sheng, China | IV | Valliyodan et al. (2021) |
| PI 437838 | *Dt1* | *Dt2* | *E1* | *e2* | *E3* | Indeterminate | DV-254 | *G. max* | Landrace | Other | Russian Federation | II | Valliyodan et al. (2021) |
| PI 567225 | *Dt1* | *Dt2* | *E1* | *e2* | *E3* | Indeterminate | Kisinevskaja 90 | *G. max* | Landrace | Other | Moldova | 0 | Valliyodan et al. (2021) |
| PI 171428 | *Dt1* | *Dt2* | *E1* | *e2* | *E3* | Indeterminate | Large Yellow Soybean | *G. max* | Landrace | Other | Beijing Shi, China | IV | Valliyodan et al. (2021) |
| PI 437127 A | *Dt1* | *Dt2* | *E1* | *E2* | *E3* | Indeterminate | Imeretinscaja | *G. max* | Landrace | Other | Georgia | IV | Valliyodan et al. (2021) |
| PI 417091 | *Dt1* | *Dt2* | *E1* | *E2* | *E3* | Indeterminate | Kuro mame | *G. max* | Landrace | Other | Japan | II | Valliyodan et al. (2006) |
| PI 437863 A | *Dt1* | *Dt2* | *E1* |  | *E3* | Indeterminate | DV-2841 | *G. max* | Elite | Other | China | II | Valliyodan et al. (2006) |
| PI 438112 B | *Dt1* | *Dt2* |  |  |  | Indeterminate | VIR 2623 | *G. max* | Landrace | Other | China | III | Valliyodan et al. (2021) |
| PI 549041 A | *Dt1* | *Dt2* |  |  |  | Indeterminate | ZYD 2709 | *G. max* | Landrace | Other | Liaoning Sheng, China | III | Valliyodan et al. (2021) |
| PI 054608 -1 | *Dt1* | *Dt2* |  |  |  | Indeterminate |  | *G. max* | Landrace | Other |  | II | Valliyodan et al. (2021) |
| PI 407716 | *Dt1* | *Dt2* | *E1* | *e2* | *e3* | Semi-determinate | Jin nung No. 3 | *G. max* | Landrace | Other | Jilin Sheng, China | I | Zhou et al. (2015) |
| PI 391583 | *Dt1* | *Dt2* | *E1* | *e2* | *E3* | Semi-determinate | Jilin No. 10 | *G. max* | Landrace | Other | Jilin Sheng, China | II | Valliyodan et al. (2021) |
| PI 547409 | *Dt1* | *Dt2* | *e1-as* | *E2* | *E3* | Semi-determinate | L62-1251 | *G. max* | Elite | NA Cultivar | Illinois, United States | IV | Zhou et al. (2015) |
| PI 547459 | *Dt1* | *Dt2* | *e1-as* | *E2* | *E3* | Semi-determinate | L64-1081 | *G. max* | Elite | NA Cultivar | Illinois, United States | IV | Zhou et al. (2015) |
| PI 548169 | *Dt1* | *Dt2* |  |  |  | Semi-determinate | T117 | *G. max* | Landrace | Other | Illinois, United States | IV | Valliyodan et al. (2021) |
| PI 464923 | *Dt1* | *Dt2* |  |  |  | Semi-determinate | Tie Fen 16 | *G. max* | Landrace | Other | Liaoning Sheng, China | I | Valliyodan et al. (2021) |
| PI 548190 | *Dt1* | *Dt2* |  |  |  | Semi-determinate | T176 | *G. max* | Elite | NA Cultivar | Illinois, United States | II | Zhou et al. (2015) |
| PI 467343 | *Dt1* | *Dt2* |  |  |  | Semi-determinate | Yan-nong No. 2 | *G. max* | Landrace | Other | Jilin Sheng, China | I | Zhou et al. (2015) |
| PI 458510 | *Dt1* | *Dt2* | *E1* | *e2* | *E3* | Determinate | Ji Ti No. 1 | *G. max* | Landrace | Other | Liaoning Sheng, China | III | Valliyodan et al. (2021) |
| PI 464896 | *Dt1* | *Dt2* | *E1* | *e2* | *E3* | Determinate | Jou Nong No. 5 | *G. max* | Landrace | Other | Jilin Sheng, China | I | Valliyodan et al. (2021) |
| PI 603357 | *Dt1* | *Dt2* | *e1-as* | *E2* | *e3* | Determinate | Du Lu Dou | *G. max* | Landrace | Other | China | I | Zhou et al. (2015) |
| PI 476352 B | *Dt1* | *Dt2* | *e1-as* | *E2* | *E3* | Determinate | (Colnon) | *G. max* | Landrace | Other | Kyrgyzstan | II | Valliyodan et al. (2021) |
| PI 088313 | *Dt1* | *Dt2* |  |  |  | Determinate |  | *G. max* | Landrace | Other |  | II | Valliyodan et al. (2021) |
| PI 417345 B | *dt1* (R166W) | *dt2* | *E1* | *e2* | *E3* | Indeterminate | (Shou outou) | *G. max* | Landrace | Other | China | IV | Valliyodan et al. (2021) |
| PI 578493 | *dt1* (R166W) | *dt2* | *E1* | *e2* | *E3* | Indeterminate | Huang bao zhu | *G. max* | Landrace | Other | China | II | Valliyodan et al. (2021) |
| PI 548474 | *dt1* (R166W) | *dt2* | *E1* | *e2* | *E3* | Indeterminate | Nanda | *G. max* | Landrace | Other | Hwanghaipukto, Korea, North | VIII | Valliyodan et al. (2021) |
| PI 159925 | *dt1* (R166W) | *dt2* | *E1* | *E2* | *E3* | Indeterminate | Glycine H | *G. max* | Landrace | Other | Lima, Peru | VIII | Valliyodan et al. (2021) |
| PI 548198 | *dt1* (R166W) | *dt2* |  |  |  | Indeterminate | T209 | *G. max* | Landrace | Other | Illinois, United States | III | Valliyodan et al. (2021) |
| PI 587811 A | *dt1* (R166W) | *dt2* |  |  |  | Indeterminate | ZDD005777 | *G. max* | Landrace | Other | Hubei Sheng, China | VIII | Valliyodan et al. (2021) |
| PI 549040 | *dt1* (R166W) | *dt2* |  |  |  | Indeterminate | ZYD 2704 | *G. max* | Landrace | Other | Liaoning Sheng, China | IV | Valliyodan et al. (2021) |
| PI 091100 -3 | *dt1* (R166W) | *dt2* |  |  |  | Indeterminate |  | *G. max* | Landrace | Other |  | III | Valliyodan et al. (2021) |
| PI 079691 -4 | *dt1* (R166W) | *dt2* |  |  |  | Indeterminate |  | *G. max* | Landrace | Other |  | III | Valliyodan et al. (2006) |
| PI 567532 | *dt1* (R166W) | *dt2* | *E1* | *e2* | *e3* | Semi-determinate | Dai ye xiao huang dou | *G. max* | Landrace | Other | Shandong Sheng, China | IV | Valliyodan et al. (2021) |
| PI 416890 | *dt1* (R166W) | *dt2* | *E1* | *e2* | *e3* | Semi-determinate | Gokuwase natsu daizu | *G. max* | Landrace | Other | Japan | 0 | Zhou et al. (2015) |
| PI 548382 | *dt1* (R166W) | *dt2* | *E1* | *e2* | *e3* | Semi-determinate | Manitoba Brown | *G. max* | Landrace | NA Ancestor | Unknown | 00 | Zhou et al. (2015) |
| PI 506933 | *dt1* (R166W) | *dt2* | *E1* | *e2* | *E3* | Semi-determinate | Kouiku 1 | *G. max* | Landrace | Other | Japan | IV | Valliyodan et al. (2021) |
| PI 628812 | *dt1* (R166W) | *dt2* | *E1* | *e2* | *E3* | Semi-determinate | MG/BR-46 (Conquista) | *G. max* | Landrace | Other | Brazil | V | Valliyodan et al. (2021) |
| PI 154189 | *dt1* (R166W) | *dt2* | *E1* | *E2* | *e3* | Semi-determinate | No. 57 | *G. max* | Landrace | Other | Netherlands | 0 | Valliyodan et al. (2021) |
| PI 628913 | *dt1* (R166W) | *dt2* | *e1-as* | *e2* | *E3* | Semi-determinate | BR-30 | *G. max* | Landrace | Other | Brazil | VI | Valliyodan et al. (2021) |
| PI 567262 A | *dt1* (R166W) | *dt2* |  |  |  | Semi-determinate | Similar to: Gu tian type | *G. max* | Landrace | Other | Fujian Sheng, China | II | Valliyodan et al. (2021) |
| PI 423926 | *dt1* (R166W) | *dt2* |  |  |  | Semi-determinate | Tousan 72 | *G. max* | Landrace | Other | Nagano, Japan | IV | Valliyodan et al. (2021) |
| PI 317336 | *dt1* (R166W) | *dt2* |  |  |  | Semi-determinate | Shinsei | *G. max* | Landrace | Other | Hokkaidô, Japan | 0 | Zhou et al. (2015) |
| PI 507293 B | *dt1* (R166W) | *dt2* | *E1* | *e2* | *e3* | Determinate | (Shoukin ou) | *G. max* | Landrace | Other | Japan | III | Valliyodan et al. (2021) |
| PI 416838 | *dt1* (R166W) | *dt2* | *E1* | *e2* | *e3* | Determinate | Choutan | *G. max* | Landrace | Other | Japan | V | Valliyodan et al. (2021) |
| PI 549028 | *dt1* (R166W) | *dt2* | *E1* | *e2* | *e3* | Determinate | Feng da li | *G. max* | Landrace | Other | Liaoning Sheng, China | V | Valliyodan et al. (2021) |
| PI 603397 | *dt1* (R166W) | *dt2* | *E1* | *e2* | *e3* | Determinate | Hei qi huang da dou | *G. max* | Landrace | Other | China | IV | Valliyodan et al. (2021) |
| PI 548356 | *dt1* (R166W) | *dt2* | *E1* | *e2* | *e3* | Determinate | Kanro | *G. max* | Landrace | Other | Phyeongyang, Korea, North | II | Valliyodan et al. (2021) |
| PI 438471 | *dt1* (R166W) | *dt2* | *E1* | *e2* | *e3* | Determinate | Fiskeby III | *G. max* | Elite | Other | Östergötlands län, Sweden | 00 | Valliyodan et al. (2006) |
| PI 603154 | *dt1* (R166W) | *dt2* | *E1* | *e2* | *e3* | Determinate | GL 2622 /96 | *G. max* | Elite | Other | Korea, North | V | Valliyodan et al. (2006) |
| PI 603170 | *dt1* (R166W) | *dt2* | *E1* | *e2* | *e3* | Determinate | GL 2683 /96 | *G. max* | Elite | Other | Korea, North | IV | Valliyodan et al. (2006) |
| PI 603175 | *dt1* (R166W) | *dt2* | *E1* | *e2* | *e3* | Determinate | GL 2688 /96 | *G. max* | Elite | Other | Korea, North | IV | Valliyodan et al. (2006) |
| PI 200471 | *dt1* (R166W) | *dt2* | *E1* | *e2* | *e3* | Determinate | Hanayome Ibaragi No. 1 | *G. max* | Elite | Other | Japan | III | Valliyodan et al. (2006) |
| PI 398593 | *dt1* (R166W) | *dt2* | *E1* | *e2* | *e3* | Determinate | KAS 390-4 | *G. max* | Elite | Other | Chungcheongbuk-do, Korea, South | V | Valliyodan et al. (2006) |
| PI 398595 | *dt1* (R166W) | *dt2* | *E1* | *e2* | *e3* | Determinate | KAS 390-5 | *G. max* | Elite | Other | Chungcheongbuk-do, Korea, South | V | Valliyodan et al. (2006) |
| PI 398610 | *dt1* (R166W) | *dt2* | *E1* | *e2* | *e3* | Determinate | KAS 390-8 | *G. max* | Elite | Other | Chungcheongbuk-do, Korea, South | V | Valliyodan et al. (2006) |
| PI 398614 | *dt1* (R166W) | *dt2* | *E1* | *e2* | *e3* | Determinate | KAS 390-9 | *G. max* | Elite | Other | Chungcheongbuk-do, Korea, South | V | Valliyodan et al. (2006) |
| PI 200508 | *dt1* (R166W) | *dt2* | *E1* | *e2* | *e3* | Determinate | Natsu Daizu | *G. max* | Elite | Other | Japan | I | Valliyodan et al. (2006) |
| PI 229343 | *dt1* (R166W) | *dt2* | *E1* | *e2* | *e3* | Determinate | Nonaka No. 1 | *G. max* | Elite | Other | Japan | IV | Valliyodan et al. (2006) |
| PI 407788 A | *dt1* (R166W) | *dt2* | *E1* | *e2* | *e3* | Determinate | ORD 8113 | *G. max* | Elite | Other | Kyonggi, Korea, South | IV | Valliyodan et al. (2006) |
| PI 398296 | *dt1* (R166W) | *dt2* | *E1* | *e2* | *e3* | Determinate | KAS 173-3 | *G. max* | Landrace | Other | Kyonggi, Korea, South | II | Zhou et al. (2015) |
| PI 407849 | *dt1* (R166W) | *dt2* | *E1* | *e2* | *e3* | Determinate | KAS 510-1 | *G. max* | Landrace | Other | Jeollabuk-do, Korea, South | III | Zhou et al. (2015) |
| PI 424391 | *dt1* (R166W) | *dt2* | *E1* | *e2* | *e3* | Determinate | KAS 521-15 | *G. max* | Landrace | Other | Jeollabuk-do, Korea, South | VI | Zhou et al. (2015) |
| PI 317334 A | *dt1* (R166W) | *dt2* | *E1* | *e2* | *e3* | Determinate | Kitamishiro | *G. max* | Landrace | Other | Hokkaidô, Japan | I | Zhou et al. (2015) |
| PI 591541 | *dt1* (R166W) | *dt2* | *E1* | *e2* | *e3* | Determinate | L74-102 | *G. max* | Elite | NA Cultivar | Illinois, United States | II | Zhou et al. (2015) |
| PI 84987 | *dt1* (R166W) | *dt2* | *E1* | *e2* | *e3* | Determinate | Oni Hadaka | *G. max* | Landrace | Other | Saitama, Japan | III | Zhou et al. (2015) |
| PI 591431 | *dt1* (R166W) | *dt2* | *E1* | *e2* | *e3* | Determinate | OT94-49 | *G. max* | Elite | NA Cultivar | Ontario, Canada | 0 | Zhou et al. (2015) |
| PI 603336 | *dt1* (R166W) | *dt2* | *E1* | *e2* | *e3* | Determinate | Qing pi si li huang | *G. max* | Landrace | Other | China | II | Zhou et al. (2015) |
| PI 437685 D | *dt1* (R166W) | *dt2* | *E1* | *e2* | *E3* | Determinate | (Phun-zhun) | *G. max* | Landrace | Other | China | III | Valliyodan et al. (2021) |
| PI 430595 | *dt1* (R166W) | *dt2* | *E1* | *e2* | *E3* | Determinate | 58-161 | *G. max* | Landrace | Other | China | IV | Valliyodan et al. (2021) |
| PI 567788 | *dt1* (R166W) | *dt2* | *E1* | *e2* | *E3* | Determinate | Bienville | *G. max* | Elite | Other | Louisiana, United States | VIII | Valliyodan et al. (2021) |
| PI 464912 | *dt1* (R166W) | *dt2* | *E1* | *e2* | *E3* | Determinate | Dan Dou 1 | *G. max* | Landrace | Other | Liaoning Sheng, China | IV | Valliyodan et al. (2021) |
| PI 490766 | *dt1* (R166W) | *dt2* | *E1* | *e2* | *E3* | Determinate | Dawudou | *G. max* | Landrace | Other | Hebei Sheng, China | III | Valliyodan et al. (2021) |
| PI 171451 | *dt1* (R166W) | *dt2* | *E1* | *e2* | *E3* | Determinate | Kosamame | *G. max* | Landrace | Other | Kanagawa, Japan | VII | Valliyodan et al. (2021) |
| PI 549021 A | *dt1* (R166W) | *dt2* | *E1* | *e2* | *E3* | Determinate | Na hei dou | *G. max* | Landrace | Other | Liaoning Sheng, China | III | Valliyodan et al. (2021) |
| PI 507088 | *dt1* (R166W) | *dt2* | *E1* | *e2* | *E3* | Determinate | Nattou Kotsubu | *G. max* | Landrace | Other | Japan | VI | Valliyodan et al. (2021) |
| PI 417215 | *dt1* (R166W) | *dt2* | *E1* | *e2* | *E3* | Determinate | Ooita Aki Daizu 2 | *G. max* | Landrace | Other | Japan | VIII | Valliyodan et al. (2021) |
| PI 471938 | *dt1* (R166W) | *dt2* | *E1* | *e2* | *E3* | Determinate | 197 | *G. max* | Elite | Other | Nepal | V | Valliyodan et al. (2006) |
| PI 464920 B | *dt1* (R166W) | *dt2* | *E1* | *e2* | *E3* | Determinate | (Jin Dou 33) | *G. max* | Elite | Other | Liaoning Sheng, China | III | Valliyodan et al. (2006) |
| PI 594012 | *dt1* (R166W) | *dt2* | *E1* | *e2* | *E3* | Determinate | Heuksatangdu | *G. max* | Elite | Other | Korea, South | V | Valliyodan et al. (2006) |
| PI 416937 | *dt1* (R166W) | *dt2* | *E1* | *e2* | *E3* | Determinate | Houjaku Kuwazu | *G. max* | Elite | Other | Japan | VI | Valliyodan et al. (2006) |
| PI 518664 | *dt1* (R166W) | *dt2* | *E1* | *e2* | *E3* | Determinate | Hutcheson | *G. max* | Elite | NA Cultivar | Virginia, United States | V | Valliyodan et al. (2006) |
| PI 548657 | *dt1* (R166W) | *dt2* | *E1* | *e2* | *E3* | Determinate | Jackson | *G. max* | Elite | NA Ancestor | North Carolina, United States | VII | Valliyodan et al. (2006) |
| PI 408105 A | *dt1* (R166W) | *dt2* | *E1* | *e2* | *E3* | Determinate | KAS 633-19 | *G. max* | Elite | Other | Gyeongsangbuk-do, Korea, South | IV | Valliyodan et al. (2006) |
| PI 417015 | *dt1* (R166W) | *dt2* | *E1* | *e2* | *E3* | Determinate | Kawanagare (Iwate) | *G. max* | Elite | Other | Iwate, Japan | III | Valliyodan et al. (2006) |
| PI 548342 | *dt1* (R166W) | *dt2* | *E1* | *e2* | *E3* | Determinate | Higan | *G. max* | Landrace | Other | Tôkyô, Japan | IV | Zhou et al. (2015) |
| PI 548985 | *dt1* (R166W) | *dt2* | *E1* | *e2* | *E3* | Determinate | Kershaw | *G. max* | Elite | NA Cultivar | South Carolina, United States | VI | Zhou et al. (2015) |
| PI 399043 | *dt1* (R166W) | *dt2* | *E1* | *e2* | *E3* | Determinate | KLS 903 | *G. max* | Landrace | Other | Jeju-teukbyeoljachido, Korea, South | III | Zhou et al. (2015) |
| PI 196166 | *dt1* (R166W) | *dt2* | *E1* | *e2* | *E3* | Determinate | No. 2296 | *G. max* | Landrace | Other | Korea, South | V | Zhou et al. (2015) |
| PI 548477 | *dt1* (R166W) | *dt2* | *E1* | *e2* | *E3* | Determinate | Ogden | *G. max* | Elite | NA Ancestor | Tennessee, United States | VI | Zhou et al. (2015) |
| PI 603384 | *dt1* (R166W) | *dt2* | *E1* | *e2* | *E3* | Determinate | Ping ding xiang | *G. max* | Landrace | Other | China | III | Zhou et al. (2015) |
| PI 548485 | *dt1* (R166W) | *dt2* | *E1* | *e2* | *E3* | Determinate | Roanoke | *G. max* | Landrace | NA Ancestor | Jiangsu Sheng, China | VII | Zhou et al. (2015) |
| PI 506862 | *dt1* (R166W) | *dt2* | *E1* | *E2* | *e3* | Determinate | Karikei 86 | *G. max* | Elite | Other | Japan | IV | Valliyodan et al. (2021) |
| PI 209334 | *dt1* (R166W) | *dt2* | *E1* | *E2* | *e3* | Determinate | No. 9 | *G. max* | Landrace | Other | Hokkaidô, Japan | III | Valliyodan et al. (2021) |
| PI 548456 | *dt1* (R166W) | *dt2* | *E1* | *E2* | *e3* | Determinate | Haberlandt | *G. max* | Landrace | NA Ancestor | Phyeongyang, Korea, North | VI | Zhou et al. (2015) |
| PI 561701 | *dt1* (R166W) | *dt2* | *E1* | *E2* | *E3* | Determinate | G88-20092 | *G. max* | Landrace | Other | Georgia, United States | IV | Valliyodan et al. (2021) |
| PI 548978 | *dt1* (R166W) | *dt2* | *E1* | *E2* | *E3* | Determinate | Gail | *G. max* | Elite | Other | Texas, United States | VI | Valliyodan et al. (2021) |
| PI 594922 | *dt1* (R166W) | *dt2* | *E1* | *E2* | *E3* | Determinate | Graham | *G. max* | Elite | Other | North Carolina, United States | V | Valliyodan et al. (2021) |
| PI 542972 | *dt1* (R166W) | *dt2* | *E1* | *E2* | *E3* | Determinate | H7190 | *G. max* | Landrace | Other | United States | VII | Valliyodan et al. (2021) |
| PI 561387 | *dt1* (R166W) | *dt2* | *E1* | *E2* | *E3* | Determinate | Kosuzu | *G. max* | Landrace | Other | Japan | V | Valliyodan et al. (2021) |
| PI 559932 | *dt1* (R166W) | *dt2* | *E1* | *E2* | *E3* | Determinate | Manokin | *G. max* | Elite | Other | Maryland, United States | IV | Valliyodan et al. (2021) |
| PI 548473 | *dt1* (R166W) | *dt2* | *E1* | *E2* | *E3* | Determinate | Monetta | *G. max* | Landrace | Other | Jiangsu Sheng, China | VII | Valliyodan et al. (2021) |
| PI 594512 A | *dt1* (R166W) | *dt2* | *E1* | *E2* | *E3* | Determinate | Bian zi jiang se dou | *G. max* | Elite | Other | Sichuan Sheng, China | VII | Valliyodan et al. (2006) |
| PI 548667 | *dt1* (R166W) | *dt2* | *E1* | *E2* | *E3* | Determinate | Essex | *G. max* | Elite | NA Cultivar | Virginia, United States | V | Valliyodan et al. (2006) |
| PI 647086 | *dt1* (R166W) | *dt2* | *E1* | *E2* | *E3* | Determinate | N8001 | *G. max* | Elite | NA Cultivar | North Carolina, United States | VIII | Valliyodan et al. (2006) |
| PI 553047 | *dt1* (R166W) | *dt2* | *E1* | *E2* | *E3* | Determinate | Gordon | *G. max* | Elite | NA Cultivar | Georgia, United States | VII | Zhou et al. (2015) |
| PI 533602 | *dt1* (R166W) | *dt2* | *E1* | *E2* | *E3* | Determinate | Lloyd | *G. max* | Elite | NA Cultivar | Arkansas, United States | VI | Zhou et al. (2015) |
| PI 548604 | *dt1* (R166W) | *dt2* | *E1* | *E2* | *E3* | Determinate | Pershing | *G. max* | Elite | NA Cultivar | Missouri, United States | IV | Zhou et al. (2015) |
| PI 591432 | *dt1* (R166W) | *dt2* | *E1* |  | *e3* | Determinate | OT94-51 | *G. max* | Elite | NA Cultivar | Ontario, Canada | 0 | Zhou et al. (2015) |
| PI 546044 | *dt1* (R166W) | *dt2* | *e1-as* | *e2* | *e3* | Determinate | OT89-06 | *G. max* | Elite | NA Cultivar | Ontario, Canada | 0 | Zhou et al. (2015) |
| PI 591433 | *dt1* (R166W) | *dt2* | *e1-as* | *e2* | *e3* | Determinate | OT94-37 | *G. max* | Elite | NA Cultivar | Ontario, Canada | 0 | Zhou et al. (2015) |
| PI 86024 | *dt1* (R166W) | *dt2* | *e1-as* | *e2* | *E3* | Determinate | Daidzuhinshu satei | *G. max* | Landrace | Other | Hokkaidô, Japan | III | Zhou et al. (2015) |
| PI 547779 | *dt1* (R166W) | *dt2* | *e1-as* | *e2* | *E3* | Determinate | L72D-4110 | *G. max* | Elite | NA Cultivar | Illinois, United States | II | Zhou et al. (2015) |
| PI 360957 | *dt1* (R166W) | *dt2* | *e1-as* | *e2* |  | Determinate | Karafuto No. 1 | *G. max* | Landrace | Other | Hokkaidô, Japan | 00 | Valliyodan et al. (2021) |
| PI 232992 | *dt1* (R166W) | *dt2* | *e1-as* | *E2* | *e3* | Determinate | Kono-Kuradaizu | *G. max* | Landrace | Other | Saga, Japan | III | Valliyodan et al. (2021) |
| PI 540552 | *dt1* (R166W) | *dt2* | *e1-as* | *E2* | *e3* | Determinate | Hoyt | *G. max* | Elite | NA Cultivar | Ohio, United States | II | Zhou et al. (2015) |
| PI 507180 | *dt1* (R166W) | *dt2* | *e1-as* | *E2* | *E3* | Determinate | Rikuu 21 | *G. max* | Landrace | Other | Japan | IV | Valliyodan et al. (2021) |
| PI 548565 | *dt1* (R166W) | *dt2* | *e1-as* | *E2* | *E3* | Determinate | Gnome | *G. max* | Elite | NA Cultivar | Ohio, United States | II | Zhou et al. (2015) |
| PI 547488 | *dt1* (R166W) | *dt2* | *e1-as* | *E2* | *E3* | Determinate | L67-3207 | *G. max* | Elite | NA Cultivar | Illinois, United States | IV | Zhou et al. (2015) |
| PI 549026 | *dt1* (R166W) | *dt2* |  |  | *E3* | Determinate | Gao li huang | *G. max* | Landrace | Other | Liaoning Sheng, China | V | Valliyodan et al. (2021) |
| PI 548178 | *dt1* (R166W) | *dt2* |  |  |  | Determinate | T145 | *G. max* | Landrace | Other | Illinois, United States | III | Valliyodan et al. (2021) |
| PI 548256 | *dt1* (R166W) | *dt2* |  |  |  | Determinate | T279 | *G. max* | Landrace | Other | Mississippi, United States | VII | Valliyodan et al. (2021) |
| PI 436684 | *dt1* (R166W) | *dt2* |  |  |  | Determinate | Tie-feng 8 | *G. max* | Landrace | Other | Liaoning Sheng, China | III | Valliyodan et al. (2021) |
| PI 598358 | *dt1* (R166W) | *dt2* |  |  |  | Determinate | TN 5-95 | *G. max* | Elite | Other | Tennessee, United States | V | Valliyodan et al. (2021) |
| PI 507458 | *dt1* (R166W) | *dt2* |  |  |  | Determinate | Tousan kei BL 521 | *G. max* | Landrace | Other | Japan | IV | Valliyodan et al. (2021) |
| PI 507480 | *dt1* (R166W) | *dt2* |  |  |  | Determinate | Tousan kei YL 24 | *G. max* | Landrace | Other | Japan | IV | Valliyodan et al. (2021) |
| PI 417479 | *dt1* (R166W) | *dt2* |  |  |  | Determinate | Yougetsu | *G. max* | Landrace | Other | Japan | IV | Valliyodan et al. (2021) |
| PI 083942 | *dt1* (R166W) | *dt2* |  |  |  | Determinate |  | *G. max* | Landrace | Other |  | V | Valliyodan et al. (2021) |
| PI 088468 | *dt1* (R166W) | *dt2* |  |  |  | Determinate |  | *G. max* | Landrace | Other |  | II | Valliyodan et al. (2021) |
| PI 095860 | *dt1* (R166W) | *dt2* |  |  |  | Determinate |  | *G. max* | Landrace | Other |  | VI | Valliyodan et al. (2021) |
| PI 090479 P | *dt1* (R166W) | *dt2* |  |  |  | Determinate |  | *G. max* | Landrace | Other |  | IV | Valliyodan et al. (2021) |
| PI 196175 | *dt1* (R166W) | *dt2* |  |  |  | Determinate | Yu tae | *G. max* | Elite | Other | Korea, South | V | Valliyodan et al. (2006) |
| PI 84631 | *dt1* (R166W) | *dt2* |  |  |  | Determinate | S-56 | *G. max* | Landrace | Other | Kyonggi, Korea, South | III | Zhou et al. (2015) |
| PI 243541 | *dt1* (R166W) | *dt2* |  |  |  | Determinate | Shakujo | *G. max* | Landrace | Other | Akita, Japan | IV | Zhou et al. (2015) |
| PI 423954 | *dt1* (R166W) | *dt2* |  |  |  | Determinate | Shirome | *G. max* | Landrace | Other | Kumamoto, Japan | 0 | Zhou et al. (2015) |
| PI 536635 | *dt1* (R166W) | *dt2* |  |  |  | Determinate | Sprite | *G. max* | Elite | NA Cultivar | Ohio, United States | III | Zhou et al. (2015) |
| PI 507355 | *dt1* (R166W) | *dt2* |  |  |  | Determinate | Tokei 423 | *G. max* | Landrace | Other | Hokkaidô, Japan | I | Zhou et al. (2015) |
| PI 417398 | *dt1* (R166W) | *dt2* |  |  |  | Determinate | Touhou torotou | *G. max* | Landrace | Other | China | III | Zhou et al. (2015) |
| PI 594301 | *dt1* (R166W) | *dt2* |  |  |  | Determinate | Toyomusume | *G. max* | Landrace | Other | Japan | I | Zhou et al. (2015) |
| PI 508266 | *dt1* (R166W) | *dt2* |  |  |  | Determinate | Young | *G. max* | Elite | NA Cultivar | North Carolina, United States | VI | Zhou et al. (2015) |
| PI 594579 | *dt1* (R166W) | *dt2* |  |  |  | Determinate | Zhong he tian cheng dou | *G. max* | Landrace | Other | Hunan Sheng, China | V | Zhou et al. (2015) |
| PI 080837 | *dt1* (R166W) | *dt2* |  |  |  | Determinate |  | *G. max* | Landrace | Other |  | IV | Zhou et al. (2015) |
| PI 407801 | *dt1* (R166W) | *dt2* |  |  |  | Determinate |  | *G. max* | Landrace | Other | Kyonggi, Korea, South | VI | Zhou et al. (2015) |
| PI 567611 | *dt1** (P113L) | *dt2* | *E1* | *e2* | *E3* | Indeterminate | Ba yue zha | *G. max* | Elite | Other | Henan Sheng, China | IV | Valliyodan et al. (2006) |
| PI 587666 | *dt1** (P113L) | *dt2* | *E1* | *e2* | *E3* | Indeterminate | Er dao zao | *G. max* | Landrace | Other | Anhui Sheng, China | VI | Zhou et al. (2015) |
| PI 416971 | *dt1** (P113L) | *dt2* | *E1* | *e2* | *E3* | Indeterminate | Kaifuu gyuumou ou 1 | *G. max* | Landrace | Other | Japan | IV | Zhou et al. (2015) |
| PI 404187 | *dt1** (P113L) | *dt2* |  |  |  | Indeterminate | Suj nii hun mao ju | *G. max* | Landrace | Other | China | II | Valliyodan et al. (2021) |
| PI 567231 | *dt1** (P113L) | *dt2* |  |  |  | Indeterminate | WJK-PRC-46 | *G. max* | Landrace | Other | Sichuan Sheng, China | VIII | Valliyodan et al. (2021) |
| PI 567675 | *dt1** (P113L) | *dt2* |  |  |  | Indeterminate | Yu cheng xiao tie jiao huang | *G. max* | Landrace | Other | Henan Sheng, China | IV | Valliyodan et al. (2021) |
| PI 567685 | *dt1** (P113L) | *dt2* |  |  |  | Indeterminate | Zhong mou tie jiao er cao | *G. max* | Landrace | Other | Henan Sheng, China | IV | Valliyodan et al. (2021) |
| PI 605869 A | *dt1** (P113L) | *dt2* |  |  |  | Indeterminate | Sample 140 | *G. max* | Elite | Other | Lào Cai, Vietnam | V | Valliyodan et al. (2006) |
| PI 567780 B | *dt1** (P113L) | *dt2* | *E1* | *e2* | *E3* | Semi-determinate | (Tong shan zheng ji dou) | *G. max* | Landrace | Other | Jiangsu Sheng, China | IV | Valliyodan et al. (2021) |
| PI 567690 | *dt1** (P113L) | *dt2* | *E1* | *e2* | *E3* | Semi-determinate | Fu yang (7) | *G. max* | Elite | Other | Anhui Sheng, China | III | Valliyodan et al. (2006) |
| PI 538386 A | *dt1** (P113L) | *dt2* | *E1* | *E2* | *E3* | Semi-determinate | 1886 | *G. max* | Landrace | Other | Hebei Sheng, China | III | Valliyodan et al. (2021) |
| PI 437695 A | *dt1** (P113L) | *dt2* |  |  |  | Semi-determinate | S-185 | *G. max* | Landrace | Other | China | I | Valliyodan et al. (2021) |
| PI 594456 A | *dt1** (P113L) | *dt2* |  |  |  | Semi-determinate | Xiao jin huang | *G. max* | Landrace | Other | Sichuan Sheng, China | III | Valliyodan et al. (2021) |
| PI 594777 | *dt1** (P113L) | *dt2* | *E1* | *e2* | *e3* | Determinate | Liu yue huang | *G. max* | Landrace | Other | Yunnan Sheng, China | IV | Zhou et al. (2015) |
| PI 587712 B | *dt1** (P113L) | *dt2* | *E1* | *e2* | *E3* | Determinate | (E dou No. 1) | *G. max* | Landrace | Other | Hubei Sheng, China | V | Valliyodan et al. (2021) |
| PI 548447 | *dt1** (P113L) | *dt2* | *E1* | *e2* | *E3* | Determinate | Cherokee | *G. max* | Landrace | Other | Zhejiang Sheng, China | VIII | Valliyodan et al. (2021) |
| PI 518727 | *dt1** (P113L) | *dt2* | *E1* | *e2* | *E3* | Determinate | Ju huang | *G. max* | Landrace | Other | Guangdong Sheng, China | VI | Valliyodan et al. (2021) |
| PI 603596 | *dt1** (P113L) | *dt2* | *E1* | *e2* | *E3* | Determinate | Bai hua gu tian dou | *G. max* | Landrace | Other | China | III | Zhou et al. (2015) |
| PI 567189 A | *dt1** (P113L) | *dt2* | *E1* | *e2* | *E3* | Determinate | Ekhabac | *G. max* | Landrace | Other | Vietnam | IV | Zhou et al. (2015) |
| PI 603463 | *dt1** (P113L) | *dt2* | *e1-as* | *e2* | *E3* | Determinate | Dong jie No. 1 | *G. max* | Landrace | Other | China | II | Valliyodan et al. (2021) |
| PI 603458 A | *dt1** (P113L) | *dt2* |  |  |  | Determinate | Shui dou | *G. max* | Landrace | Other | China | IV | Valliyodan et al. (2021) |
| PI 379618 | *dt1** (P113L) | *dt2* |  |  |  | Determinate | TC 1 | *G. max* | Landrace | Other | Taiwan | V | Valliyodan et al. (2021) |
| PI 578504 | *dt1** (P113L) | *dt2* |  |  |  | Determinate | Xiang dou No. 3 | *G. max* | Landrace | Other | China | II | Valliyodan et al. (2021) |
| PI 592954 | *dt1** (P113L) | *dt2* |  |  |  | Determinate | ZDD 11242 | *G. max* | Elite | Other | China | II | Valliyodan et al. (2021) |
| PI 603488 | *dt1** (P113L) | *dt2* |  |  |  | Determinate | ZDD19294 | *G. max* | Landrace | Other | China | III | Valliyodan et al. (2021) |
| PI 597464 | *dt1** (P113L) | *dt2* |  |  |  | Determinate | Zhe chun No. 3 | *G. max* | Landrace | Other | Zhejiang Sheng, China | II | Valliyodan et al. (2021) |
| PI 342434 | *dt1** (P113L) | *dt2* |  |  |  | Determinate |  | *G. max* | Landrace | Other | Iwate, Japan | V | Valliyodan et al. (2021) |
| PI 587752 | *dt1** (P113L) | *dt2* |  |  |  | Determinate | Xian ning dong huang dou jia | *G. max* | Landrace | Other | Hubei Sheng, China | V | Zhou et al. (2015) |
| PI 594629 | *dt1** (P113L) | *dt2* |  |  |  | Determinate | Xiao hua lian | *G. max* | Landrace | Other | Guizhou Sheng, China | VI | Zhou et al. (2015) |
| PI 588053 A | *dt1** (P113L) | *dt2* |  |  |  | Determinate | Xiao li huang | *G. max* | Landrace | Other | Guangdong Sheng, China | V | Zhou et al. (2015) |
| PI 603516 | *dt1** (P113L) | *dt2* |  |  |  | Determinate | Xiao ma yi dan | *G. max* | Landrace | Other | China | VI | Zhou et al. (2015) |
| PI 603756 | *dt1** (P113L) | *dt2* |  |  |  | Determinate | ZDD05996 | *G. max* | Landrace | Other | China | II | Zhou et al. (2015) |
| PI 548656 | *dt1** (P113L)^¶^ | *dt2* | *E1* | *E2* | *E3* | Determinate | Lee | *G. max* | Elite | Other | Mississippi, United States | VI | Valliyodan et al. (2021) |
| PI 548658 | *dt1** (P113L)^¶^ | *dt2* | *E1* | *E2* | *E3* | Determinate | Lee 74 | *G. max* | Elite | Other | Arkansas, United States | VI | Valliyodan et al. (2021) |
| PI 165675 | *dt1** (P113L)^¶^ | *dt2* | *E1* | *E2* | *E3* | Determinate | Nanking 332 | *G. max* | Landrace | Other | Jiangsu Sheng, China | VII | Valliyodan et al. (2021) |
| PI 548445 | *dt1** (P113L)^¶^ | *dt2* | *E1* | *E2* | *E3* | Determinate | CNS | *G. max* | Landrace | NA Ancestor | Jiangsu Sheng, China | VII | Zhou et al. (2015) |
| PI 189873 | *dt1-t1* (R130K) | *dt2* | *E1* | *e2* | *e3* | Indeterminate | Miko Saumon | *G. max* | Landrace | Other | France | 0 | Valliyodan et al. (2021) |
| PI 378680 E | *dt1-t1* (R130K) | *dt2* | *E1* | *e2* | *E3* | Indeterminate | (VNIIMK 9186) | *G. max* | Landrace | Other | Russian Federation | I | Valliyodan et al. (2021) |
| PI 606374 | *dt1-t1* (R130K) | *dt2* | *E1* | *e2* | *E3* | Indeterminate | Cao bang 8 | *G. max* | Landrace | Other | Vietnam | IV | Valliyodan et al. (2021) |
| PI 361093 | *dt1-t1* (R130K) | *dt2* | *E1* | *e2* | *E3* | Indeterminate | Novosadska Br. 1 | *G. max* | Landrace | Other | Serbia | I | Valliyodan et al. (2021) |
| PI 567307 | *dt1-t1* (R130K) | *dt2* | *E1* | *E2* | *E3* | Indeterminate | Hei huang dou | *G. max* | Landrace | Other | Gansu Sheng, China | IV | Valliyodan et al. (2021) |
| PI 437485 | *dt1-t1* (R130K) | *dt2* |  |  |  | Indeterminate | VIR 1048 | *G. max* | Landrace | Other | Primorye, Russian Federation | II | Valliyodan et al. (2021) |
| PI 404182 | *dt1-t1* (R130K) | *dt2* |  |  |  | Indeterminate | Sin i tu li rau | *G. max* | Landrace | Other | China | III | Zhou et al. (2015) |
| PI 567525 | *dt1-t1* (R130K) | *dt2* | *E1* | *e2* | *E3* | Semi-determinate | Cao qing huang dou | *G. max* | Landrace | Other | Shandong Sheng, China | II | Valliyodan et al. (2021) |
| PI 437653 | *dt1-t1* (R130K) | *dt2* | *E1* | *e2* | *E3* | Semi-determinate | Er-da-li | *G. max* | Landrace | Other | China | II | Zhou et al. (2015) |
| PI 594451 | *dt1-t1* (R130K) | *dt2* | *E1* | *e2* | *E3* | Semi-determinate | Liu yue bao | *G. max* | Landrace | Other | Sichuan Sheng, China | III | Zhou et al. (2015) |
| PI 437814 A | *dt1-t1* (R130K) | *dt2* | *e1-as* | *E2* | *E3* | Semi-determinate | Anda | *G. max* | Landrace | Other | China | II | Valliyodan et al. (2021) |
| PI 438309 | *dt1-t1* (R130K) | *dt2* |  |  |  | Semi-determinate | VIR 3017 | *G. max* | Landrace | Other | China | I | Valliyodan et al. (2021) |
| PI 437793 | *dt1-t1* (R130K) | *dt2* |  |  |  | Semi-determinate | VIR 3024 | *G. max* | Landrace | Other | China | II | Valliyodan et al. (2021) |
| PI 567651 | *dt1-t1* (R130K) | *dt2* |  |  |  | Semi-determinate | Shang cai er cao ping ding shi | *G. max* | Elite | Other | Henan Sheng, China | IV | Valliyodan et al. (2006) |
| PI 153262 | *dt1-t1* (R130K) | *dt2* |  |  |  | Semi-determinate | Roumanie | *G. max* | Landrace | Other | Belgium | 0 | Zhou et al. (2015) |
| PI 361087 | *dt1-t1* (R130K) | *dt2* | *E1* | *e2* | *E3* | Determinate | Medias 23 | *G. max* | Landrace | Other | Romania | I | Valliyodan et al. (2021) |
| PI 567298 | *dt1-t1* (R130K) | *dt2* | *E1* | *e2* | *E3* | Determinate | Chan yao dou | *G. max* | Landrace | Other | Gansu Sheng, China | V | Zhou et al. (2015) |
| PI 603698 J | *dt1-t1* (R130K) | *dt2* | *e1-as* | *e2* | *E3* | Determinate | (Dan yang shui bai dou) | *G. max* | Landrace | Other | China | 0 | Valliyodan et al. (2021) |
| PI 437240 | *dt1-t1* (R130K) | *dt2* | *e1-as* | *e2* | *E3* | Determinate | CSchi 1069 | *G. max* | Landrace | Other | Moldova | 0 | Valliyodan et al. (2021) |
| PI 567226 | *dt1-t1* (R130K) | *dt2* | *e1-as* | *e2* | *E3* | Determinate | Harkovskaja Zernoukosnaja | *G. max* | Landrace | Other | Russian Federation | 00 | Valliyodan et al. (2021) |
| PI 372418 | *dt1-t1* (R130K) | *dt2* | *e1-as* | *e2* | *E3* | Determinate | Novosadska Br. 4 | *G. max* | Landrace | Other | Serbia | I | Valliyodan et al. (2021) |
| PI 438336 | *dt1-t1* (R130K) | *dt2* |  |  |  | Determinate | Sao 208 | *G. max* | Landrace | Other | Algeria | 0 | Valliyodan et al. (2021) |
| PI 548417 | *dt1-t1* (R130K) | *dt2* |  |  |  | Determinate | Soysota | *G. max* | Landrace | Other | Italy | I | Zhou et al. (2015) |
| PI 603722 | *dt1-t2* (R62S) | *dt2* | *E1* | *e2* | *E3* | Indeterminate | Nan chong ba yue huang | *G. max* | Landrace | Other | China | VIII | Valliyodan et al. (2021) |
| PI 567576 | *dt1-t2* (R62S) | *dt2* | *E1* | *e2* | *E3* | Indeterminate | Ping ding huang | *G. max* | Landrace | Other | Shandong Sheng, China | III | Valliyodan et al. (2021) |
| PI 404166 | *dt1-t2* (R62S) | *dt2* | *E1* | *E2* | *E3* | Indeterminate | Krasnoarmejskaja | *G. max* | Landrace | Other | China | III | Valliyodan et al. (2006) |
| PI 165563 | *dt1-t2* (R62S) | *dt2* | *e1-as* | *e2* | *E3* | Indeterminate | Bhart | *G. max* | Landrace | Other | Uttar Pradesh, India | VII | Valliyodan et al. (2021) |
| PI 548171 | *dt1-t2* (R62S) | *dt2* |  |  |  | Indeterminate | T134 | *G. max* | Elite | Other | Illinois, United States | III | Valliyodan et al. (2021) |
| PI 567352 A | *dt1-t2* (R62S) | *dt2* |  |  |  | Indeterminate | Yang yan qing dou | *G. max* | Landrace | Other | Gansu Sheng, China | IV | Valliyodan et al. (2021) |
| PI 567353 | *dt1-t2* (R62S) | *dt2* |  |  |  | Indeterminate | Yang yan ren dou | *G. max* | Landrace | Other | Gansu Sheng, China | IV | Valliyodan et al. (2021) |
| PI 089772 | *dt1-t2* (R62S) | *dt2* |  |  |  | Indeterminate |  | *G. max* | Landrace | Other |  | IV | Valliyodan et al. (2021) |
| PI 567258 | *dt1-t2* (R62S) | *dt2* | *E1* | *e2* | *e3* | Semi-determinate | NC 9173 | *G. max* | Landrace | Other | Jiangxi Sheng, China | II | Zhou et al. (2015) |
| PI 532463 B | *dt1-t2* (R62S) | *dt2* | *E1* | *e2* | *E3* | Semi-determinate | (He bei No. 1) | *G. max* | Landrace | Other | Hebei Sheng, China | III | Valliyodan et al. (2021) |
| PI 567698 A | *dt1-t2* (R62S) | *dt2* | *E1* | *e2* | *E3* | Semi-determinate | Fu yang (17) | *G. max* | Landrace | Other | Anhui Sheng, China | IV | Valliyodan et al. (2021) |
| PI 567731 | *dt1-t2* (R62S) | *dt2* | *E1* | *e2* | *E3* | Semi-determinate | Fu yang (56) | *G. max* | Elite | Other | Anhui Sheng, China | III | Valliyodan et al. (2006) |
| PI 437690 | *dt1-t2* (R62S) | *dt2* | *E1* | *E2* | *E3* | Semi-determinate | Pin-din-guan | *G. max* | Landrace | Other | China | III | Valliyodan et al. (2006) |
| PI 437679 | *dt1-t2* (R62S) | *dt2* | *E1* | *E2* | *E3* | Semi-determinate | Nan-cou | *G. max* | Landrace | Other | China | IV | Zhou et al. (2015) |
| PI 567503 | *dt1-t2* (R62S) | *dt2* | *E1* | *E2* | *E3* | Semi-determinate | Niu mao huang | *G. max* | Landrace | Other | Hebei Sheng, China | IV | Zhou et al. (2015) |
| PI 548162 | *dt1-t2* (R62S) | *dt2* |  |  |  | Semi-determinate | T48 | *G. max* | Landrace | Other | Illinois, United States | IV | Valliyodan et al. (2021) |
| PI 437725 | *dt1-t2* (R62S) | *dt2* |  |  |  | Semi-determinate | Te-zu-gan | *G. max* | Landrace | Other | China | IV | Valliyodan et al. (2006) |
| PI 438498 | *dt1-t2* (R62S) | *dt2* |  |  |  | Semi-determinate | Sable | *G. max* | Landrace | Other | United States | IV | Zhou et al. (2015) |
| PI 603497 | *dt1-t2* (R62S) | *dt2* | *E1* | *e2* | *e3* | Determinate | Hua dou | *G. max* | Landrace | Other | China | III | Valliyodan et al. (2021) |
| PI 103088 | *dt1-t2* (R62S) | *dt2* | *E1* | *e2* | *e3* | Determinate | Ming Chuan | *G. max* | Landrace | Other | Henan Sheng, China | III | Valliyodan et al. (2021) |
| PI 407742 | *dt1-t2* (R62S) | *dt2* | *E1* | *e2* | *E3* | Determinate | 16 | *G. max* | Landrace | Other | Shaanxi Sheng, China | V | Valliyodan et al. (2021) |
| PI 587588 B | *dt1-t2* (R62S) | *dt2* | *E1* | *e2* | *E3* | Determinate | (Tai xing niu mao huang yi) | *G. max* | Landrace | Other | Jiangsu Sheng, China | V | Valliyodan et al. (2021) |
| PI 567383 | *dt1-t2* (R62S) | *dt2* | *E1* | *e2* | *E3* | Determinate | Da ke huang dou | *G. max* | Landrace | Other | Shaanxi Sheng, China | V | Valliyodan et al. (2021) |
| PI 597476 | *dt1-t2* (R62S) | *dt2* | *E1* | *e2* | *E3* | Determinate | Deogyukong | *G. max* | Elite | Other | Korea, South | V | Valliyodan et al. (2021) |
| PI 548696 | *dt1-t2* (R62S) | *dt2* | *E1* | *e2* | *E3* | Determinate | Dortchsoy 67 | *G. max* | Elite | Other | Arkansas, United States | V | Valliyodan et al. (2021) |
| PI 567346 | *dt1-t2* (R62S) | *dt2* | *E1* | *e2* | *E3* | Determinate | Niu mao huang dou | *G. max* | Landrace | Other | Gansu Sheng, China | V | Valliyodan et al. (2021) |
| PI 602991 | *dt1-t2* (R62S) | *dt2* | *E1* | *e2* | *E3* | Determinate | Niu jiao qi da hei dou | *G. max* | Landrace | Other | Shandong Sheng, China | IV | Zhou et al. (2015) |
| PI 548402 | *dt1-t2* (R62S) | *dt2* | *E1* | *E2* | *E3* | Determinate | Peking | *G. max* | Landrace | Other | Beijing Shi, China | IV | Valliyodan et al. (2021) |
| PI 597478 B | *dt1-t2* (R62S) | *dt2* | *e1-as* | *e2* | *E3* | Determinate | (Paldalkong) | *G. max* | Landrace | Other | Korea, South | III | Valliyodan et al. (2021) |
| PI 594170 B | *dt1-t2* (R62S) | *dt2* | *e1-as* | *E2* | *e3* | Determinate | (Geden shirazu) | *G. max* | Landrace | Other | Akita, Japan | I | Valliyodan et al. (2021) |
| PI 594880 | *dt1-t2* (R62S) | *dt2* |  |  |  | Determinate | Song zi dou | *G. max* | Landrace | Other | Yunnan Sheng, China | V | Valliyodan et al. (2021) |
| PI 567604 A | *dt1-t2* (R62S) | *dt2* |  |  |  | Determinate | Xin huang dou | *G. max* | Landrace | Other | Shandong Sheng, China | IV | Valliyodan et al. (2021) |
| PI 592952 | *dt1-t2* (R62S) | *dt2* |  |  |  | Determinate | ZDD 10095 | *G. max* | Landrace | Other | China | III | Valliyodan et al. (2021) |
| PI 612730 | *dt1-t2* (R62S) | *dt2* |  |  |  | Determinate | Zhong huong No. 10 | *G. max* | Landrace | Other | China | II | Valliyodan et al. (2021) |
| PI 507354 | *dt1-t2* (R62S) | *dt2* |  |  |  | Determinate | Tokei 421 | *G. max* | Landrace | Other | Hokkaidô, Japan | I | Valliyodan et al. (2006) |

^†^ The genotype at stem termination type loci (*Dt1* and *Dt2*) were assigned based on the allele state at each of the genes from publicly available whole genome resequenced datasets.

^‡^ The allele status at the maturity gene loci (*E1*, *E2* and *E3*) were obtained from a previous work of Langewisch et al. (2014).

^§^ The detail information of soybean accessions used in the analysis were downloaded from the USDA Germplasm Resources Information Network (GRIN).

^¶^ Two missense mutations, both P113L and L67Q, at the *Dt1* locus were found.

**Supplementary Table 2.** Detail information of field experiments for evaluating morphological characteristics depending on genotype combination in three different latitudinal environments in 2019 and 2020

|  | **Field Experiments** | | |
| --- | --- | --- | --- |
|  | **19GA** | **20TN** | **20MO** |
| **Year** | 2019 | 2020 | 2020 |
| **Location** | University of Georgia Iron Horse Plant Science Farm, Watkinsville, Georgia | East Tennessee Agriculture and Education Center, Knoxville, Tennessee | South Farm Research Center, Columbia, Missouri |
| **Latitude** | 33.72˚N, -83.30˚W | 35.96˚N, -83.86˚W | 38.91˚N, -92.29˚W |
| **Experimental design** | Completely randomized design | Randomized complete block design | Randomized complete block design |
| Number of lines | 21 | 20 | 14 |
| Number of genotypes | 7 | 7 | 5 |
| Boarder rows | No | No | Yes |
| Number of rows per plot | 2 | 2 | 3 |
| Number of replications | none | 3 | 3 |
| Planting density | 48 seeds per row | 200 seeds per row | 50 seeds per row |
| Row length | 6 ft (1.83 m) | 16 ft (4.88 m) | 7 ft (2.13 m) |
| Row spacing | 30 in (76.2 cm) | 30 in (76.2 cm) | 30 in (76.2 cm) |
| **Planting date** | 5^th^ June, 2019 | 27^th^ May, 2020 | 2^nd^ June, 2020 |
| **Number of plants per plot** | 5 | 10 | 5 |
| Plant height | Measured | Measured | Measured |
| Number of nodes | Measured | Measured | Measured |
| Stem diameter (SD; mm) | Measured | Measured | Measured |
| Lodging | Measured | Measured | Measured |
| Days to maturity (DTM) |  | Measured | Measured |
| Days to flowering (DTF) |  |  | Measured |
| Number of pods at stem tip (Pod; ea) | Measured | Measured | Measured |
| Raceme length (RL; cm) |  | Measured | Measured |
| Number of branches (Branch; ea) |  |  | Measured |

**Supplementary Table 3**. Summary of recombinant inbred line (RIL) population development with plans per year for the developing experimental lines of the four RIL populations used in this study

|  | **Population** | | | |
| --- | --- | --- | --- | --- |
|  | **KB17-16** | **KB17-17** | **KB17-7** | **KB17-8** |
| **Pedigree** | L91-8052^†^ x Jake^‡^ | L91-8060^†^ x Jake^‡^ | LG90-2550^†^ x Jake^‡^ | KG90-2550^†^ x Ellis^‡^ |
| **Target genotype** | dt1-t1_E1 | dt1-t2_E1 | Dt2_E1 | Dt2_E1 |
| **Population development history** | | | | |
| In summer 2017 | Crosses made at the South Farm Research Center, Columbia, MO | Crosses made at the South Farm Research Center, Columbia, MO | Crosses made at the South Farm Research Center, Columbia, MO | Crosses made at the South Farm Research Center, Columbia, MO |
| In winter 2017 | Planted F_1_ seeds in Costa Rica | Planted F_1_ seeds in Costa Rica | Planted F_1_ seeds in Costa Rica | Planted F_1_ seeds in Costa Rica |
|  | Allele at *Dt1* locus was confirmed from DNA extracted from each of F1 plants | Allele at *Dt1* locus was confirmed from DNA extracted from each of F_1_ plants | Allele at *Dt2* locus was confirmed from DNA extracted from each of F_1_ plants | Allele at *Dt2* locus was confirmed from DNA extracted from each of F_1_ plants |
| In spring 2018 | Bulk harvested F_2_ seeds in Costa Rica | Bulk harvested F_2_ seeds in Costa Rica | Bulk harvested F_2_ seeds in Costa Rica | Bulk harvested F_2_ seeds in Costa Rica |
|  |  |  | Planted F_2_ seeds again in Costa Rica | Planted F_2_ seeds again in Costa Rica |
|  |  |  | Allele at *Dt1* locus was confirmed from DNA extracted from each of F_2_ plants | Allele at *Dt1* locus was confirmed from DNA extracted from each of F_2_ plants |
|  |  |  | Harvested F_2:3_ seeds from the three selected F_2_ plants by single seed thresh | Harvested F_2:3_ seeds from the four selected F_2_ plants by single seed thresh |
| In summer 2018 | Planted F_2_ seeds at the South Farm Research Center, Columbia, MO | Planted F_2_ seeds at the South Farm Research Center, Columbia, MO | Planted F_2:3_ seeds the South Farm Research Center, Columbia, MO | Planted F_2:3_ seeds at the South Farm Research Center, Columbia, MO |
|  |  |  | Allele at *E1* locus was confirmed from DNAs extracted from F_2:3_ plants | Allele at *E1* locus was confirmed from DNAs extracted from F_2:3_ plants |
| In winter 2018 | Harvested F_2:3_ seeds by single plant thresh | Harvested F_2:3_ seeds by single plant thresh | Harvested F_2:4_ seeds from the selected by pod pick from 10 plants per plot (total 30 pods were harvested) | Harvested F_2:4_ seeds from the selected by pod pick from 10 plants per plot (total 40 pods were harvested) |
|  | Alleles at *dt1* and *E1* loci were confirmed from DNAs extracted from three F_2:3_ seeds | Alleles at *dt1* and *E1* loci were confirmed from DNAs extracted from three F_2:3_ seeds^§^ |  |  |
|  | Planted F_2:3_ seeds in Costa Rica | Planted F_2:3_ seeds in Costa Rica | Planted F_2:4_ seeds in Costa Rica | Planted F_2:4_ seeds in Costa Rica |
| In spring 2019 | Harvested F_3:4_ seeds by single plant thresh | Harvested F_3:4_ seeds by single plant thresh | Harvested F_4:5_ seeds by single seed thresh | Harvested F_4:5_ seeds by single seed thresh |
|  |  | Homozygosity of *dt1-t2* (R62S) allele was confirmed from DNAs extracted from three F_3:4_ seeds |  |  |
| In summer 2019 | Planted F_3:4_ seeds in progeny rows for field experiment 19GA | Planted F_3:4_ seeds in progeny rows for field experiment 19GA | Planted F_4:5_ seeds in progeny rows for field experiment 19GA | Planted F_4:5_ seeds in progeny rows for field experiment 19GA |
|  | Morphological characteristics were measured from five randomly selected plants per experimental line | Morphological characteristics were measured from five randomly selected plants per experimental line | Morphological characteristics were measured from five randomly selected plants per experimental line | Morphological characteristics were measured from five randomly selected plants per experimental line |
| In winter 2019 | Bulk harvested F_3:5_ seeds | Bulk harvested F_3:5_ seeds | Bulk harvested F_4:6_ seeds | Bulk harvested F_4:6_ seeds |
| In summer 2020 | Planted F_3:5_ seeds for the field experiment 20TN | Planted F_3:5_ seeds for the field experiment 20TN | Planted F_4:6_ seeds for the field experiment 20TN | Planted F_4:6_ seeds for the field experiment 20TN |
|  | Morphological characteristics were measured from ten randomly selected plants per plot | Morphological characteristics were measured from ten randomly selected plants per plot | Morphological characteristics were measured from ten randomly selected plants per plot | Morphological characteristics were measured from ten randomly selected plants per plot |

^†^ Donor parents of each of target alleles on stem termination type for each population

^‡^ Donor parents of the functional *E1* allele for each population

^§^ Genotypes having heterozygous *dt1-t2* (R62S) with homozygous *E1* were selected, since there were no plants having homozygous *dt1-t2* (R62S) and *E1*
